# C-Reactive Protein: Pathophysiology, Diagnosis, False Test Results and a Novel Diagnostic Algorithm for Clinicians

**DOI:** 10.3390/diseases11040132

**Published:** 2023-09-28

**Authors:** Dimitra S. Mouliou

**Affiliations:** 38500 Volos, Magnesia, Greece; demymoole@gmail.com

**Keywords:** C-Reactive Protein, CRP, biochemical properties, forms, pathophysiology, diagnosis, biomarker, diagnostic assays, false results, algorithm, systemic inflammation

## Abstract

The current literature provides a body of evidence on C-Reactive Protein (CRP) and its potential role in inflammation. However, most pieces of evidence are sparse and controversial. This critical state-of-the-art monography provides all the crucial data on the potential biochemical properties of the protein, along with further evidence on its potential pathobiology, both for its pentameric and monomeric forms, including information for its ligands as well as the possible function of autoantibodies against the protein. Furthermore, the current evidence on its potential utility as a biomarker of various diseases is presented, of all cardiovascular, respiratory, hepatobiliary, gastrointestinal, pancreatic, renal, gynecological, andrological, dental, oral, otorhinolaryngological, ophthalmological, dermatological, musculoskeletal, neurological, mental, splenic, thyroid conditions, as well as infections, autoimmune-supposed conditions and neoplasms, including other possible factors that have been linked with elevated concentrations of that protein. Moreover, data on molecular diagnostics on CRP are discussed, and possible etiologies of false test results are highlighted. Additionally, this review evaluates all current pieces of evidence on CRP and systemic inflammation, and highlights future goals. Finally, a novel diagnostic algorithm to carefully assess the CRP level for a precise diagnosis of a medical condition is illustrated.

## 1. Introduction

Over the last few years, multifarious conventional and point-of-care molecular diagnostic assays have shaped the accuracy of medical diagnosis to a great extent. Nowadays, numerous hematological, biochemical and serological diagnostic tests are widely performed on various clinical specimens in order to estimate the functional capacity of several critical organs and systems, identify the presence of foreign agents, and monitor the course of various (auto)immune processes and the overall immune status of a case [1,2,3,4,5,6,7,8,9,10].

C-Reactive Protein (CRP) is supposed to be an accredited benchmark for physicians, to reveal or rule out inflammation, and multifarious scientific endeavors have been made so as to detect the direct pleiotropic functions of this protein. The use of CRP as the most important and critical immunochemical marker of several medical conditions, including infections such as sepsis, physiological organ diseases, various autoimmune disorders, malignancies and other health conditions, has become widely popular [11,12,13,14,15,16,17,18,19,20]. Hitherto, a plethora of molecular diagnostic assays have been developed for the detection of CRP [21,22].

The aim of this critical state-of-the-art review is to summarize the potential biochemical, pathophysiological and diagnostic spectrum of CRP, Moreover, the current CRP diagnostic assays and reasons of potential misdiagnoses and possible false test results are thoroughly presented. Furthermore, the author expresses her critical expert opinion, and, finally, a novel diagnostic algorithm to carefully assess the CRP level is thoroughly illustrated.

## 2. Current Evidence on Potential Biochemical Properties and Genetics of C-Reactive Protein

### 2.1. Forms of C-Reactive Protein

CRP was discovered by Tillett and Francis of Rockefeller University in 1930; they reported a non-protein somatic fraction called “fraction c” that precipitated in high titers after isolation from the serum of patients infected with *pneumococcus*, which was biochemically distinct from previously known capsular polysaccharide and nucleoprotein fractions detectable by a specific antibody response [23]. About a decade later, Avery and McCarty reported a substance elevated in the serum of cases with a pathogenic spectrum of inflammatory stimuli [24]. The name “*C-Reactive-Protein*” arose by virtue of further research by Volanakis and Kaplan, who identified the precise ligand for CRP in the pneumococcal “c” polysaccharide as phosphocholine, which is derived from the teichoic acid of the pneumococcal cell wall [25].

Phylogenetically, CRP is highly conserved with homologues in various vertebrates and invertebrates. Various physicochemical and immunological research studies on the tertiary and quaternary structure of CRP have concluded that the microenvironment can modify its architecture. Heretofore, with the exception of genetic variations, it has been revealed that CRP can exist in at least three main distinct forms, including a monomeric CRP form, often called “modified CRP”, that consists of a unique subunit; a “native” pentameric form; and a multimeric form composed of ten or more subunits [26]. Additionally, some other dissociated forms of CRP have been reported, such as dimers, trimers, tetramers, and even other non-native pentameric configurations that have been formed again due to alterations of the microenvironment [27,28]. Apart from the pentameric ring-like form that was discovered mostly on ligand-containing membranes in a calcium-dependent manner, a study on the combination of size-exclusion chromatography and electron microscopy revealed the small globulin-like form and the fibril-like structures [29]. It was suggested that the CRP can switch between these various forms under certain conditions, and this fact serves as evidence for the structural basis of multiple functions of CRP [29].

Moreover, even though CRP was known to be a non-glycosylated protein, differentially glycosylated forms of CRP have been reported in various pathological conditions [28]. The structural integrity of CRP can also be altered because of biotinylation and denaturation [30]. Generally, several post-translational CRP modifications may lead to different protein stability and structure. Laboratory research on CRP has also revealed new forms, and the pentameric protein was found to express neo-CRP antigenicity upon various treatments, resulting in different microenvironments, but these data suggest that ligands—especially phosphocholine and antibodies—are not enough to induce neoantigenic expressions [31]. CRP multimers have been reported in vitro, along with pentamers, and it was estimated that their concentration would increase after the removal of calcium ions [32]. Crystallographic research on calcium-depleted CRP has attributed the decamer to interactions between two CRP A faces of two independent pentamers [33]. The native pentamer along with the modified monomer form are estimated to prevail; thus, this review focuses on these two forms.

### 2.2. Structure of C-Reactive Protein

#### 2.2.1. The Monomeric or “Modified” CRP

X-ray crystallography has revealed that each monomer is a non-glycosylated globular subunit of 206 amino acid residues and has a molecular weight of ~23 kDa (minimum 20,946 kDa) [28,34]. It has an isoelectric point of 5.4 in contrast to the pentamer, which has an isoelectric point of 6.4. The monomer is folded into two antiparallel β-sheets with a flattened jelly roll topology similar to lectins, especially concanavalin, as well as a recognition face with a phosphocholine binding site that consists of two coordinated calcium ions adjacent to a hydrophobic pocket [35,36]. The calcium ions are bound 4 Å apart by protein sidechains deriving from long loops collected at the concave face, designated as face B, of that sheet, which is the area of ligand binding [37]. The -NH_2_ terminal residue of CRP is pyrrolidonecarboxylic acid, while the -COOH terminus is Pro. Furthermore, cysteine residues that form the intrachain disulfide bond are 61 residues apart in CRP primary sequence (36 and 97 residues) [34,36].

The other site is designated as face A, and carries a single α helix, thus the pentameric disc shows five helices on one face and ten calcium ions on the other [36]. Additionally, each subunit is rotated by 22° toward the fivefold axis in a way that the helices of face A are 5 Å closer to the axis, while the calcium sites of face B move out by an equivalent amount [36]. The A face includes also includes a furrow accentuated by CRP because of the substitution of few smaller sidechains and by the reorientation of some others, and defines a region that is 24 Å long, 7.5 Å deep and 12.4 Å wide [34]. The side walls consist of Ser5, Arg6, Gln203, Pro206, Trp187, Arg188, Asn160, Gly177, Leu176, Tyr175, His95, and Asp112, whereas the bottom is lined with Asn158, His38, Leu37, Val94, and Asp112 [34]. The furrows follow the monomers’ curvature and edge together closely as they enter the central pentameric pore. Also, the furrow’s outer part is positively charged, but its inner part terminates halfway through the pore at residue Asp112, resulting in a negatively charged ring lining the pore [34]. Mutagenesis research has revealed Asp112 to be a crucial residue for the recognition of C1q by CRP [38].

CRP is a calcium-dependent protein; regarding the calcium-binding cites of CRP, the first region includes Asp60, Asn61, Glu138, Asp140, and the main chain carbonyl oxygen of residue 139, yet Asp60 provides only one oxygen to the calcium ion (total of five), whereas the second equivalent cite residues contain Gln138, Asp140, and Gln150 [34]. Other data from CRP synthetic peptides show a direct binding of these two ions to a specific peptide of residues 134–148 [28]. When both calcium sites are vacant in CRP, residues 140–150 form a large loop away from the body of the molecule, exposing an otherwise hidden site of proteolysis [34]. X-ray crystallography has revealed also that these calcium ions are coordinated by Asp60, Asn61, and by residues Glu138, Gln139, Asp140, Glu147, and Gln150 in the loop; on the contrary, the past primary literature data suggest that in the first structure of CRP, the sidechain of Glu147 is not positioned to coordinate the calcium ion [28,39,40].

Primary difference maps calculated from reflection data sets accumulated from the crystals grown in the existence of phosphocholine revealed very good density for one phosphocholine molecule in each of the five CRP monomers, while the principal interaction takes place between the phosphate group of phosphocholine and the bound calcium ions [34]. Two oxygens interact directly with each calcium, leading the third oxygen away from the binding site in vitro. This orientation allows for CRP and phosphocholine interactions when the phosphate moiety is in ester linkage with other molecules, whereas the remaining phosphocholine part extends from this area and runs along the CRP surface, which is packed against Phe66, approaching the sidechain of residue Glu81 [34]. The interval between the positively charged quaternary nitrogen of phosphocholine and the acidic sidechain of Glu81 is 3.8 Å, indicating that this interaction is a critical determinant of phosphocholine binding [34]. Phe-66 and Glu-81 are the two key residues mediating the binding of phosphocholine to CRP [28]. Phe-66 accomplishes hydrophobic interactions with three methyl groups of phosphocholine, while Glu-81 is located on the opposite end of the pocket where it interacts with the nitrogen atom of choline, and the significance of both residues has been verified by mutagenesis studies [28,39]. Additionally, the Thr76 residue is a determinant of the phosphocholine-binding site as it creates the appropriately sized pocket to harbor phosphocholine [28,40]. The small sidechain of Thr76 leaves a hydrophobic cavity (8.7 × 7 × 3.5 Å) on the outer area of CRP that is lined with atoms from Glu81, Gly79, Asn61, and Thr76. This pocket encourages the creation of branched phosphocholine analogues with bulky substituents at the second position that could be bound with a higher affinity than phosphocholine [34]. Moreover, Trp67, Lys57, and Arg58 do not directly contact phosphocholine but seem to be required for the proper conformation of the binding site [40].

A small peptide at the N-terminus and another one near the C-terminus are absent in glycosylated human CRP, and their cleavage exposes two potential glycosylation sites, which are located on the opposite face from the phosphocholine-binding face of CRP [28]. In a study, the loss of these peptides exposed two possible glycosylation sites on a cleft floor, thereby keeping the protein–protein interactions in pentamers and calcium-dependent phosphocholine-binding qualitatively unaffected [41].

Furthermore, the literature data highlight that the mutagenesis of Glu42 or Pro115 due to hydrogen peroxide, which are residues in the intersubunit contact region in the pentamer, to Gln42 and Ala115, respectively, also converts CRP into biomolecules that can bind to a variety of immobilized, denatured, and aggregated proteins, thus resulting in a different final pentameric form of CRP [42]. Another study found that Thr173 and Asn186 residues are important for the binding of CRP to FcγRIIa and FcγRI [43]. Lys114, like Leu176, was found to be implicated in proteins binding to FcγRI but not FcγRIIa, whereas single mutations at amino acid positions Lys114, Asp169, Thr173, Tyr175, and Leu176 affected C1q binding to CRP, and all these results indicate a possible overlapping of these sites [43]. It is estimated that more literature data on the structure of the monomer of CRP will be evident in the near future.

#### 2.2.2. The Pentameric or “Native” CRP

**Figure 1:**
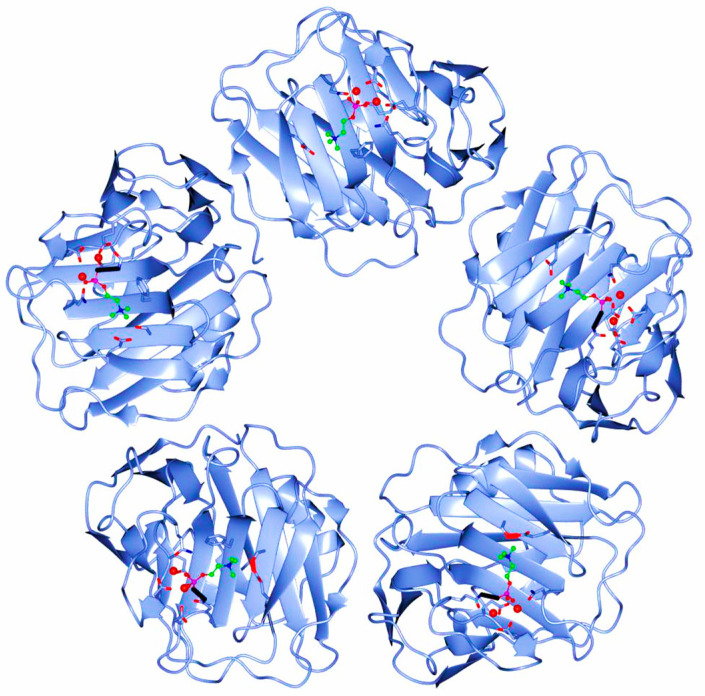
Pentameric structure of C-Reactive Protein (ridge helix highlighted in red).

The human CRP is a pentameric member of the short pentraxin family, also known as pentraxin 1. The term “pentraxin” is derived from the Greek word for five (penta) and berry (ragos) and is related to the radial symmetry of five monomers forming a ring. It has also been used to illustrate the family of related proteins with this specific structure. Pentraxins are some highly conserved proteins—according to evolution evidence—and are supposed to precede the development of the adaptive immune response. The pentameric native form of CRP is the arrangement of five non-covalently associated monomers into a symmetric cyclic pattern around a central pore, thereby creating a discoidal and planar configuration, as seen in Figure 1.

It must be highlighted that all CRP forms are “native” as they are produced by human cells, but since the pentameric form is supposed to be the initially synthesized form, this is specifically referred to as native in current literature.

The binding of CRP to a phosphocholine-containing ligand activates the classical complement pathway up to the stage of C3 convertase, and Asp112 and Tyr175, which are residues along the boundaries of a cleft extended from each protomer’s center to the central pore of the pentamer, play critical roles in the formation of the C1q-binding site [28,34,35]. The opposite face of this pentraxin is the effector face, where complement C1q binding occurs and also Fcγ receptors are supposed to bind. A three-dimensional model for CRP with C1q binding has proposed that the acme of the predominantly positively charged C1q head domain interacts with the principally negatively charged central cavity of the CRP pentamer, and that its globular head spans the pore and interacts with two of the five protomers [44]. The strict steric requirements for this interaction imply that the ideal binding is accompanied by various slight conformational CRP changes based on each CRP ligand [44].

It was previously discussed that under certain circumstances, such as in acidic pH in vitro, CRP adopts a different pentameric configuration that exposes a hidden ligand binding site for non-phosphocholine ligands, which also enables CRP to bind to immobilized, denatured, and aggregated proteins, regardless of the identity of the native biomolecule [42]. Moreover, the literature data suggest that the fibril-like structures, which have been previously reported, are formed by the face-to-face stacking of pentamers in a number from several to hundreds, whereas the freshly purified CRPs created short single-strand fibrils that are stored for at least several days, resulting in long and bundled fibrils [29].

### 2.3. Genetics of C-Reactive Protein

CRP genetic locus has been mapped to the proximal long arm of chromosome 1 in the 1q23.2 region [45]. The CRP gene sequence was simultaneously discovered in 1985 by two different research teams, both reporting that it consists of 1 intron separating 2 exons [46,47]. Nucleotide sequence analysis has revealed that after coding for a signal peptide of 18 amino acids and the first two amino acids of the mature CRP, there is a long-length intron of 278 base pairs followed by the nucleotide sequence for the remaining 204 amino acids, which is the second exon, followed by a stop codon [45,47]. This unusual intron contains a poly(A) stretch that is 16 nucleotides long and a poly(GT) region that is 30 nucleotides long, which could adopt the Z-form of DNA, on the positive strand [47]. The long intron includes a GT repeat sequence, the stretch of which is polymorphic in length [45]. The mRNA cap site has been reported to be located 104 nucleotides from the beginning of the signal peptide, and there is a 3′ noncoding region with a length of 1.2 kb pairs [47]. Additionally, the gene has a typical promoter that contains the sequences TATAAAT and CAAT 29, and is 81 base pairs upstream of the cap site [47].

Despite some polymorphisms, no allelic variations or other genetic deficiencies are identified for the CRP gene. Individuals with specific allele combinations have two-fold lower baseline CRP levels, possibly due to subsequent DNA structural changes that have an impact on transcription [48]. Single Nucleotide Polymorphisms (SNPs) across the CRP gene have highlighted a significant variation in CRP levels among CRP-divergent haplotypes. CRP has also shown both decreased and/or elevated levels in various promoters [49]. Within the promoter, multiple polymorphisms have been identified in transcription factor binding E-box sites, all of which have resulted in various baseline circulating CRP titers and responses by other genes that encode cytokines that influence its synthesis, such as IL-6, IL-1, and TNF-α [45]. A systematic resequencing of the CRP gene showed as many as 40 SNPs, resulting in as many as 29 different haplotypes, with by far the highest nucleotide variance observed in African Americans, thus highlighting that the CRP gene is polymorphic [50]. Generally, multifarious CRP genetic polymorphisms have been identified in different genetic loci, which can alter CRP blood concentrations, including common CRP or new variants as well as promoter polymorphisms; these variants have been associated with an increased risk for lung cancer, coronary heart disease, and other conditions [51,52,53,54,55,56]. Nevertheless, such studies establishing associations between genetic variants and a disease risk need to be re-evaluated since potential direct molecular changes in CRP functions after genetic alterations have not yet been precisely recorded. Moreover, CRP genetic polymorphisms can affect other nearby genes; in humans, the serum amyloid P component gene and CRP gene map to 1q23.2 within an interval linked to Systemic Lupus Eruthrematosis (SLE) as well as a polymorphism related to decreased basal CRP, was also associated with the development of SLE [57].

The induction of CRP in hepatocytes is initially regulated at the transcriptional level by the cytokine Interleukin-6 (IL-6), and this effect can be enhanced by Interleukin-1β (IL-1β) since IL-6 is not sufficient by itself [58]. Although some promoter haplotypes have been associated with elevated CRP levels, this association is not IL-6-dependent, but rather reflects a change in basal promoter activity [50]. IL-6 and IL-1β regulate thew expression of several acute phase protein genes via the activation of the transcription factors STAT3, C/EBP family members, and Rel proteins belonging to NF-κB family [50,58]. The regulation of every acute phase gene is unique because of the cytokine-induced and -determined interactions of these and other transcription factors with their promoters. As a result, STAT3 is the major factor for fibrinogen genes, NF-κB is essential for the serum amyloid A gene; for CRP, the C/EBP family members C/EBPβ and C/EBPδ are crucial for induction [39]. It is important to mention that CRP and serum amyloid A share crucial amino acids, with the second one selectively modulating platelet reactivity and also down-regulating at least one CRP biological capacity. In addition to C/EBP binding sites, the direct promoter region of the CRP gene includes binding sites for STAT3 and Rel proteins [39]. Interactions between such factors that result in the enhanced stable DNA binding of C/EBP family members cause the maximum induction of the gene [39]. Additionally, transcription is regulated through E-box elements that bind the promoter to USF1, and such elements’ SNPs affect CRP levels. It is critical to note that in vitro studies on the regulation of CRP gene expression have mostly focused on primary hepatocytes, hepatocyte cell lines, or various transfected cell lines; thus, the extrahepatic production of the protein, which can show different gene expressions, has not been thoroughly studied yet [45].

## 3. Current Evidence on Potential Pathobiology of C-Reactive Protein

### 3.1. Synthesis of C-Reactive Protein

CRP is predominantly synthesized in the right lobe of the liver mainly in response to IL-6 and, to a lesser degree, Il-1β, IL-17, and TNF-α, as well as stress signals in parallel with vascular stimulation related to tissue damage [28]. Both IL-6 and Il-1β the control expression of the CRP gene through the activation of the C/EBP family members C/EBPβ and C/EBPδ, which are crucial transcription factors for the induction of CRP production [26,58]. The products of some activated monocytes in hep 3B cells induce the synthesis of human serum amyloid A protein as well as CRP, but not by IL-1β, TNF-α, nor several other hepatocyte-stimulating factor procedures. In liver cells, the pentamer is retained in the endoplasmic reticulum as it binds to two carboxylesterases, gp60a and gp50b, and during the resting non-inflammatory condition, the protein is released from the reticulum; yet, after a slight rapid increase of certain inflammatory cytokine levels, this binding decreases, and the protein is rapidly secreted. Generally, CRP is initially synthesized in its monomeric form, and then these monomers are created in the endoplasmic reticulum. CRP was initially supposed to be solely produced by the liver, but currently, various pieces scientific evidence reveal some other extrahepatic sites for CRP production, including neurons, adipose tissue, intestines, renal cortical tubules and lung epithelial cells, coronary and other smooth muscle cells, atherosclerotic plaques (mostly by smooth muscle cells and macrophages), Kupffer cells, active peripheral blood monocytes, (alveolar) macrophages, and lymphocytes [26,35,59]. Moreover, data from Integrated Proteomics regarding CRP gene expression in normal tissues and cell lines from ProteomicsDB and MOPED highlight some CRP concentration in serum and plasma, the stomach, colon, rectum, synovial fluid, kidneys, spleen, lungs, adrenals, pancreas, islet of Langerhans, gall bladder, ovaries, testes, and liver, as well as some lower expressions in the tonsils, frontal cortex, spinal cord, retinas, heart, esophagus, vitreous tumor, uterus, cervix, placenta skin and milk; low concentrations are seen in several other tissues [60]. Bgee data report approximately 90 tissues that are able to produce CRP (including those previously mentioned) [60]. Specifically, cortical tubules and glomerular cells have been shown to locally express CRP by rejection but not in acute tubular necrosis kidneys, with inflamed kidneys possibly being an unknown site where CRP can be produced [61]. The nitric oxide-induced expression of CRP in islet cells and pancreas cells has also been reported in a study on rats [62]. Additionally, some other inflammatory cytokines—apart from the liver-related ones—have been proposed to stimulate extrahepatic CRP production.

It is believed that CRP is synthesized as a homopentameric protein that can irreversibly dissociate at sites of inflammation, and also that this conversion is mediated by activated platelets. Prior to receiving stress signals, which initiate new protein synthesis, liver cells will slowly release basal CRP titers that were pre-synthesized and stored in intracellular vesicles, and following synthesis and circulation release, serum CRP titers tend to elevate significantly 6–12 h post initial stimulation, elevating at as much as 1000-fold or more within 24–72 h as a result of both synthesis and release, with a half-life of approximately 19 h [35,63]. When the stimulus for increased CRP production completely ceases, the blood CRP concentration decreases rapidly, to almost the same level as the CRP clearance rate; however, it is important to highlight that this response by CRP to stimulus is non-specific and is triggered by several disorders [26]. Generally, CRP concentration in blood is mainly determined by its synthesis rate [35]. Yet, the mechanisms regulating production at extrahepatic sites are unknown, and it is possible that they do not have an impact on the plasma levels of CRP, although it has also been proposed that these CRP sites may underlie the lower and more sustained CRP concentrations that are important risk for other medical conditions [26,35,39].

In humans, females have higher serum CRP titers than males, whereas in mice, the human CRP expression transgene follows the opposite pattern. In healthy adults, the normal CRP concentrations vary between 0.8 mg/L and 3.0 mg/L; nevertheless, some healthy adults show increased CRP at 10 mg/L [64]. Subjects in the general population show stable CRP concentrations characteristic for each individual, CRP concentrations also increase with age, perhaps due to subclinical conditions, whereas there are no seasonal variations of CRP titers [26,64]. Despite the last seasonal independence, some studies on twins reveal a crucial heritable component in baseline CRP titers regardless of both age and body mass index. Also, interindividual varieties in blood CRP titers were found to be ~40% heritable. Moreover, even though elevated CRP levels are related to various medical conditions, liver failure and the administration of certain drugs affect CRP production. Concerning healthy individuals, the normal production rate is 1.5 μg/kg-h, whereas during underlying medical conditions, its synthesis rate in reported to vary between 43.3 μg/kg h and 103.4 μg/kg h (i.e., a 30–70-fold increase). These calculations reveal that an average individual will synthesize 2.4 mg of CRP/day, elevating up to 174 mg/day after an exacerbating event [63]. CRP’s fractional catabolic rate is unaffected by its plasma concentration, indicating that alterations in CRP serum titers during an acute inflammatory response cause an increased production rate, and not on an elevated rate at which it is utilized. Moreover, IL-1 family and IL-6 gene SNPs, and the polymorphic GT repeat of the CRP gene affect the usual CRP production and titers for individuals with no underlying medical illnesses.

### 3.2. Functions of C-Reactive Protein

CRP is considered to be a protein of the innate immune system that provides baseline protection as a pattern recognition biomolecule and also as a modulator of host defense responses, including tissue barriers, vascular activation, phagocytic responses, and amplification mechanisms. Such host defenses feed into and manage specific responses of the acquired immune system; thus, this protein has been widely analyzed as a molecule contributing to both positive and negative immune responses to essentially all disease etiologies [63]. The precise functions of both CRP forms at sites of inflammation have yet to be defined overall. Nevertheless, it has been proposed that a structural change in CRP and the resulting shift from the ligand-recognition function of CRP of its pentameric conformation to another ligand-recognition function in its non-native conformation takes place at sites of inflammation [65].

The native pentameric CRP is a substrate for the formation of less soluble monomeric CRP. When the pentamer binds to an activated lipid membrane, initially using its calcium-dependent binding specificity for ligands expressing phosphocholine, biochemical forces lead to its dissociation, inducing structural rearrangements that expose a cryptic binding site on the dissociated CRP monomers for cholesterol molecules found in lipid rafts [35,65]. Generally, membranes of apoptotic or activated cells, extracellular vesicles, and liposomes can aid in the dissociation of the pentamer into its monomeric form. Membrane phosphocholine groups are the most accessible for native CRP binding after the phospholipase A2 of the lipid bilayer cleaves an acyl chain from a phospholipid, creating the detergent-like lipid monoacyl (lyso)-phosphocholine [63]. The membrane-bound pentamer is brought into juxtaposition with apolar regions of the membrane, contributing the biochemical energy needed to dissociate the pentamer. Moreover, the structural change of each CRP monomer in parallel with membrane interactions reveals a new unique binding site in it for the cholesterol of the lipid drafts that regulates fundamental cellular signaling pathways in healthy individuals and those suffering from various diseases [66]. The monomer interacts with membrane lipids and enters into cholesterol-rich lipid drafts even though it is not freely soluble in an aqueous phase; however, they are only found in body fluids related to microvesicles that are sloughed off of activated endothelial cells as part of the activated inflammatory response [67]. The conformational activation of proteins is a globally accepted procedure for multifarious biochemical systems, such as allosteric signaling, enzyme catalysis and ion-gated channel activities. Therefore, once the pentamer is coerced into dissociating into subunits, it undergoes a non-proteolytic critical conformational change into a structurally different biomolecule with distinctive biochemical, physiological, antigenic, and immunological attributes.

Interestingly, the pentamer changes into the monomeric form after interacting with activated membranes through a procedure involving an intermediate CRP form known as “monomeric CRP_m_” or “pentameric CRP*”, a form which represents the initial stages of subunit dissociation, in which the still-pentameric protein begins expressing antigenic and functional attributes, which are characteristic of monomeric CRP [68]. This molecule rapidly detaches from the cell membrane and finally dissociates in solution into mCRP_s_, which is the final and most crucial and powerful form of mCRP. This dissociation opens a neoepitope (octapeptide Phe-Thr-Lys-Pro-Gly-Leu-Trp-Pro) on the C-terminal end of each monomeric subunit, while the monomer remains mostly anchored to the cell membranes in the lipid draft microdomains, and in few extracellular microvesicles under certain conditions [68]. Until now, only a highly charged denaturant (i.e., guanidine hydrochloride) or a strong acidic pH can lead to pentamer dissociation, regardless of the presence of calcium, possibly due to their ability to disrupt the electrostatic interactions that mediate calcium binding to CRP [31]. It has been suggested that since body fluids typically contain high calcium levels, this dissociation may be difficult in vivo; however, early clues proposing that the pentamer may dissociate in vivo were obtained from observations on lipid-monolayer-bound CRP by negative-stained electron microscopy [69]. After monomeric CRP formation, calcium and other divalent cations lead to protein aggregation and precipitation. Not only a high temperature in parallel with the loss of calcium concentrations, but also high urea concentrations can lead to CRP dissociation. It has yet to be established that pentameric CRP has weak anti-inflammatory bioactivity, while the monomeric form has strong proinflammatory bioactivity [63]. Even though the pentamer dissociated on apoptotic cell membranes, the generation of lysophosphatidylcholine was required, and relevant in vivo dissociation conditions were observed for activated platelets, necrotic cell membranes, acidic pH, oxidative stress, microparticles, amyloid plaques, and neutrophil extracellular traps [26]. Such data highlight that post-translational CRP modifications are pivotal for the modulation of its proinflammatory activity. Finally, not only the autoantibodies against the unique epitopes of monomeric CRP, but also an autoimmune epitope in lupus nephritis that is exposed only in the monomer are important evidence to support the in vivo generation of monomeric CRP [70].

CRP, in its pentameric form, is mostly found in blood, whereas the monomer is found as a naturally occurring biomolecule within a wide variety of normal tissues, particularly at the intima, media, and adventitia of healthy blood vessels and also in the fibrous tissues of the skin [71,72]. Since the pentameric CRP is a substrate for the creation of monomeric CRP, the relative level of pentameric CRP measured in the bloodstream partially depends on the rate at which pentameric CRP is converted into monomeric CRP, which depends mainly on intra-subunit disulfide bonds that determine the conversion and structural stability of CRP isoforms [73]. Whilst the pentamer is resistant to proteolysis, the monomeric CRP can be proteolyzed by a variety of neutrophil-derived peptidases, and peptides can inhibit the activation of platelets and neutrophils, thereby down-regulating the potent proinflammatory activities of the intact monomer [63]. This fact highlights a direct feedback mechanism initiated by neutrophil-derived proteolysis that can immediately reverse the proinflammatory bioactivity of the monomer.

#### 3.2.1. The Monomeric or “Modified” CRP

The context- and conformation-dependent CRP functions can raise the ever-pertinent question of how CRP, a major acute phase protein, can act as a fine modulator of inflammation, whereas the different localizations and activities of distinct CRP forms may also account for its varied phenotypes in animal models as well as its elusive causal relationship with various medical conditions. Nowadays, the monomer has gained worldwide scientific attention since it is supposed to be the major conformation that acts in inflammatory lesions. Its binding to integrins αvβ3 and α4β1 has been linked to its proinflammatory effects [74]. Even though the monomer is insoluble in plasma, it localizes in inflamed tissues and areas, and amplifies proinflammatory responses through a positive feedback loop. Likewise, monomeric CRP is also much more effective than the pentameric form at inducing chemotaxis and binding to integrin in macrophages, while current knowledge reveals that the monomer exhibits more deleterious actions and seems to be more powerful regarding the effects they share in atherosclerosis [35]. The body of evidence highlights lipid drafts as the preferential membrane microdomains for mCRP anchorage, but this depends on membrane cholesterol content and is synergistically mediated by the putative cholesterol-binding consensus sequence of CRP (aa 35–47) and the C-terminal octapeptide (aa 199–206). Conversely, disarrayed lipid rafts with methyl-beta cyclodextrin/nystatin abrogated mCRP-induced cytokine release, ROS generation, and adhesion molecule expression in endothelial cells [75]. The mCRP can also be found in the bloodstream in the form of cell exosomal microparticles.

The monomeric form of CRP is attributable to the P-selection expression, synthesis, and release of Il-8 and MCP-1, particularly in endothelial cells; additionally, it augments the respiratory burst response and delays apoptosis [35,67]. Specifically, the binding of CRP to the FcγRIIb of endothelial cells inhibits the bradykinin- and insulin-mediated activation of eNOS [35]. The monomer not only increases ICAM-1, VCAM-1, E-selection, decay-accelerating factor (CD55), and membrane cofactor protein (CD46) and protectin (CD59) expression on endothelial cells, but it also activates the classic complement pathway (in both alive and necrotic cells), inhibits alternative complement pathway stimulation, and decreases the deposition of the opsonic C3b via the lectin pathway [76,77]. Generally, mCRP binds to various pathogens and triggers the complement to boost opsonization as well as clearance even before immunoglobulins, such as IgM or IgG, have been produced. Both the initiator C1q and the inhibitor C4bp of the classic complement pathway compete to bind with the monomer, with the competition controlling the local balance of the activation and inhibition of the pathway in tissues. Specifically, the monomeric CRP binds to the C4bp inhibitor, highlighting that the monomer, rather than native CRP form, is capable of providing a high degree of control over the classic complement pathway [78]. A study showed mCRP as an inhibitor of properdin in both necrotic cells and viable renal cells, thus controlling cell surface complement activation, with the authors concluding that mCRP limits tissue injury amplification by modulating the properdin-directed complement activation of damaged tissue and cells [79].

Overall, the monomer can have marked proinflammatory properties both in vitro and in vivo as it promotes monocyte chemotaxis and their recruitment, along with the recruitment of circulating leukocytes to the inflammation areas through FcγRI and FcγRIIa signaling, whilst also causing them to form ROS [80]. The monomer also binds to low-affinity IgG FcγRIIIb (CD16), which can delay apoptosis by activating the cell survival pathway in neutrophils, even at low concentrations [80]. An old in vitro study revealed that CRP hydrolysis with neutrophil-derived lysosomal enzymes yielded soluble peptides that inhibited neutrophil superoxide production, chemotaxis, degranulation, and phagocytosis [81]. Moreover, it opsonizes bacteria with an increased uptake by human macrophages and neutrophils, it enhances NO production in neutrophils through the upregulation of eNOS, and it was discussed that the monomer initiates calcium mobilization and the activation of calmodulin and PI3K to result in NO creation in neutrophils [82]. In addition, mCRP was found to stimulate leukocyte recruitment to the vessel wall, inducing the expression of vascular cell adhesion molecule-1, intercellular adhesion molecule-1, and E-selectin, as well as the production of IL-6 and IL-8 by the endothelium [83]. With the exception of inducing IL-8 production (possibly via peroxynitrite signaling in neutrophils) and preventing neutrophil apoptosis, the monomer stimulates the polarization of macrophage and T cell to proinflammatory M1 and Th1 phenotypes, megakaryocyte proliferation, oxidized LDL uptake by macrophages, and can enhance in vivo monocyte infiltration into damaged tissues [83]. Basically, mCRP inhibits the apoptosis of neutrophils, which is partially meditated by the activation of FcγRIII through the stimulation of the PI3K/PKB and ERK/MAPK-ERK(MEK) signaling pathways, resulting in the inhibition of caspase-3. This process is partially mediated by the activation of neutrophil ERK via the Ras/Raf-1/MEK cascade that upregulates complement receptor 3 (CD11b/CD18) expression, thereby enhancing adhesion to endothelial cells. mCRP inhibits chemotaxis, similar to the pentamer, yet these biomolecules interact with and bind to different neutrophil receptors. The binding of CRP to FcγRIIIb can also lead to cytokine release in both endothelium and monocytes, LPL expression in macrophages, as well as the binding of platelets to neutrophils; however, the exact form is unknown [35].

The monomer plays a critical role in atherothrombosis by promoting megacaryocytopoiesis in mice; human platelet activation, adhesion (through the activation of GP IIb/IIIa receptors) and aggregation; surface P-selectin and CD63 exposure; and glycoprotein IIb-IIIa activation. Additionally, it can alter clot dynamics as well as fibrin formation and architecture by enhancing tissue factor on the endothelial cell surface. It is also possible that elevated CRP levels lead to fibrinolytic resistance and endothelial dysfunction by affecting fibrin clot structure, highlighting its prothrombogenic effects on injured vessel walls [84,85]. Evidence for glycoprotein IIb/IIIa activation is controversial since it was also found to be required for pentameric monomerization [86]. Even if the exact CRP form is not clear in this study, both of the previous facts could be proposed as a positive feedback loop. The pentamer dissociates into its monomeric form of the surface of platelets, which also induces vasodilator-stimulated phosphoprotein dephosphorylation (Ser239), while it was found that p38 MAPK and JNK inhibitors, along with the CD36-blocking antibody, partially inhibited mCRP-induced platelet activation and aggregation [87]. Indeed, the monomer was found to aggravate post-myocardial-infarction injury by polarizing macrophages to proinflammatory phenotype through the JNK signaling pathway [88]. mCRP also stimulates platelets to release mitochondrial DNA into the anti-neutrophil cytoplasmic antibody-associated vasculitis [89]. Additionally, mCRP boosts endothelial activation and neutrophil endothelial attachment, the secretion of serotonin, and the modulation of arachidonic acid metabolism, and enhances monocyte adhesion to the collagen, fibrinogen, fibronectin, and fibronectin matrix, as well as T-lymphocyte extravasation [77,83,90]. Moreover, platelet adhesion to fibrinogen-coated plates can be enhanced in the presence of fluid phase CRP, but only in its monomeric, recombinant form; however, of course, the monomeric CRP form demonstrates opposing or overlapping activities with the pentamer, e.g., complement activation as well as endothelial cells, neutrophils and platelets activation, and its binding to ligands, including LDL, C1q and CFH [26]. Even if the precise form is yet unknown, CRP mediates tissue fibrosis in cardiovascular disease by activating TGF-β/Smad signaling through both TGF-β1-dependent and -independent mechanisms; it also upregulates PAI-1 expression and activity [91]. Notably, mCRP deposits have been found in inflamed human striated muscles and infarcted myocardium, abdominal aortic aneurysms, intact arteries, and fibrous or calcific plaques, while the atheromatous tissue deposits were larger in patients with elevated CRP blood titers and smaller in patients treated with aspirin, ACE inhibitors, or angiotensin-receptor blockers. The monomer was mainly accumulated in the necrotic core and around macrophages, T cells, and SMC clusters, as well as neovessels in atherosclerotic plaques; yet, the data do not clarify the exact CRP form to which they refer. Also, mCRP deposits were found in the atherosclerotic lesions samples from human aorta, carotid, coronary and femoral arteries [83]. Despite the fact that the monomer is considered to be insoluble in plasma, it becomes localized in inflamed tissues and amplifies proinflammatory responses via a positive feedback loop. A study revealed that CRP was localized to the nuclei of cells within the synovium of RA cases, but the neither the cell type nor the exact form were identified at that time, while other data do not indicate significant CRP localization in other pathologies, thus proposing that CRP is possibly mostly found in the fluid phase rather than becoming deposited in tissues at sites of inflammation or injury. Heretofore, however, there has been little research conducted on the localization of CRP in inflammatory cells [80], and no data are available that reveal the precise CRP form.

The monomeric form of CRP was found to be colocalized with the angiogenetic marker endoglin (CD105) in stroke cases and found to stimulate ERK1/2 phosphorylation, resulting in cell migration and the creation of tube-like structures, regardless of the CD16 axis [92]. A novel study found that the treatment of vascular cells with anti-CRP antibodies reversed the effect of uric acid on cell proliferation and migration in human vascular smooth muscle cells and NO release in human umbilical vein endothelial cells, suggesting that CRP expression may be responsible for urea-induced vascular remodeling [93]. Generally, the literature reveals that the monomer increases proliferation, migration, and tube-like structure formation in vitro and stimulates blood vessel formation in vivo. It also induces the Notch1, Notch3, vascular VEGFR2/KDR, PDGF-BB, ID1 and N-cadherin upregulation and down-regulation of VE-cadherin gene expression, and can lead to the stabilization and maturation of CYR61/CCN1, thus exerting its role in the formation, remodeling and maturation of the blood vessels [92]. Moreover, the pentamer dissociates into mCRP and triggers angiogenesis by stimulating F3 transcription and tissue factor signaling in the membranes of microvascular endothelial cells [94]. Anti-angiogenic effects are evident too, even for a CRP form that is not precise since it can decrease the survival of and induce apoptosis in endothelial progenitor cells by impairing their differentiation via inhibiting TIE2 expression, endothelial-cell-specific lectin, and VE-cadherin, and by impairing NO-dependent angiogenesis through the reduction in endothelial NO synthase and the production of proinflammatory Il-8 and monocyte chemoattractant protein-1 by endothelial cells via the NF-kB pathway. This promoting monocyte recruitment to the plaques and inducing macrophage–platelet activation and aggregation, possibly triggering plaque erosion and thrombosis [83,91]. Yet, an in vitro study clarified that interferon-responsive genes (IFI44L, IFI44, IFI27, IFI 6, MX1, and OAS2) were among the highly upregulated genes after mCRP, but not after pCRP, treatment in endothelial progenitor cells [95]. Generally, the monomer activates neutrophils, monocytes, and platelets.

Furthermore, a relatively recent study showed that in obesity, the monomeric form of CRP binds to leptin receptor [96]. Increased adiposity and plasma leptin have been correlated with CRP, thereby proposing a possible mechanism that aids in leptin resistance by which circulating CRP binds to leptin and attenuates its physiological functions [78]. However, a close relationship between leptin and CRP highlights that maybe the former is functionally involved in inflammation and atherothrombosis, apart from the pathophysiology of obesity [78]. A study investigating the effects of CRP on the production of adiponectin in 3T3-L1 adipocytes found that CRP treatment inhibited adiponectin mRNA expression and secretion in a dose- and time-dependent manner; however, the exact CRP form that resulted in such effects remained unknown [97]. Recent data have commented on the CRP-binding capacity of lectin-like oxidized low-density lipoprotein receptor-1 (LOX-1), which induces complement activation, leukocyte infiltration, and the modification of vascular response to vasodilators, mimicking a shared pathway for CRP and oxidized LDL in endothelial dysfunction; however, it is evident that mCRP reduces the uptake of acetylated LDL by the endothelium [35,98]. CRP also stimulates the release of a soluble isoform of LOX-1, both classically activated and derived from peripheral blood mononuclear cells macrophages, in people with acute coronary syndrome and possibly smokers in a process involving Fc*γ*RIIa, TNF, and ROS synthesis [99].

It was previously discussed that monomeric CRP mRNA exists in numerous extrahepatic tissues, including adipocytes, smooth muscle cells, and some inflammatory cells within atherosclerotic plaques, and recent in vitro studies have reported the detection of mCRP mRNA, particularly in U937 macrophages of atherosclerotic lesions [100]. Interestingly, an amino acid sequence called “cholesterol-binding sequence” is considered responsible for mCRP–ligand bindings, and it also has the potential to inhibit the binding of the monomer to various ligands, including lipoprotein component ApoB, cholesterol, C1q, fibronectin, collagen, and fibrinogen, thus decreasing its proinflammatory effects on leukocytes and endothelial cells, with mutations in this motif impairing such interactions [100]. Apart from binding to SARS-CoV-2 spike RBD and thus blocking ACE2R interaction, this cholesterol-binding sequence has been shown to potentiate factor H cofactor activity and modulate monomeric CRP-dependent osteoclast differentiation; these data suggest that the cholesterol-binding sequence is an effective monomeric CRP inhibitor both in vitro and in vivo [101,102]. Concerning rheumatoid arthritis (RA), mCRP was found to modulate the differentiation of osteoclasts in a conformational RANKL-dependent manner; it actively controls osteoclast differentiation via NF-κB and phospholipase C signaling, and can bind to RANKL, the major driver of osteoclast differentiation, and abrogate its activities through its cholesterol-binding sequence [102]. Yet, the SARS-CoV-2 spike protein can have multifarious interactions with various types of human proteins, and specifically, the RBD motif may be an allergen that generates toxicity [103]. A study on mCRP showed that it induced proinflammatory cytokine release, including IL-6/8, MMP13, and NOS2 in both human and murine chondrocytes, thereby proposing a possible mechanism of boosted cartilage catabolism in osteoarthritis [104].

Even if the literature data on CRP in parallel with neuroinflammation are sparse, an in vitro study demonstrated that CRP dissociated to mCRP in the presence of non-aggregated amyloid beta(42) peptide, which is supposed to cause such an effect in amyloid plaques. It was also identified in the frontal cortex of decayed AD cases in cortical Aβ plaques, whereas other data revealed its capacity to colocalize not only with CD68, NF-κB, and Il-1 in AD post-mortem cases, but also with both phosphorylated Tau (p-Tau) and Aβ(42) in mice [105]. A novel study provided additional evidence on the ApoE4-mCRP-CD31 pathway for the cross talk in peripheral inflammation and cerebrovasculature, which results in AD risk [106]. Finally, the in vitro capability of mCRP to cause Tau phosphorylation and stimulate the production of other AD precursors, including presenilin enhancer protein-2 and phosphorylated amyloid precursor protein, was demonstrated, respectively [105]. Another in vitro study examining both patient brain samples and excised mouse brain tissue in a model of dementia previously injected with mCRP found that microvessels close to the injection location (hippocampus) were strongly stained with mCRP only in the mice that had been injected with mCRP, with the authors concluding that this small blood vessel can spread it throughout the brain [107]. On the contrary, it is a fact that mCRP deposits can be found in damaged tissues, and it was strongly expressed in the brain parenchyma (neuronal nucleus as well as cytoplasm and angiogenic microvessels colocalized with CD105) of cases after ischemic stroke in the damaged core and penumbral regions, with the mCRP remaining significantly visible several months after the event [83,108]. Additionally, mCRP is expressed by stroke neovessels and possibly triggers angiogenesis, in vitro [109]. When NCAM becomes over-activated, it is related to pathological, aberrant angiogenesis. Also, mCRP can increase monolayer permeability, sprouting angiogenesis, and gap junction spacing between cells, while dorsal matrigel implants containing the monomer can produce hemorrhagic lesions [108]. It is possible that mCRP increases vascular permeability and aberrant angiogenesis, resulting in post-stroke vessel structural instability and hemorrhagic conversion. Furthermore, there exists a correlation between CRP localization in neutrophil infiltrates, especially in lesions of vasculitis and allergic encephalomyelitis, but the exact form seems doubtable [80].

**Figure 2:**
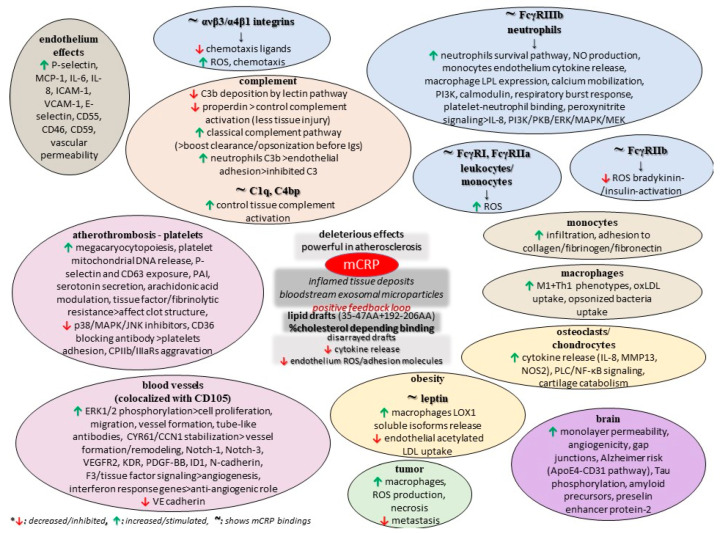
Potential functions of mCRP.

The monomer also impairs retinal pigment epithelium functionality by elevating paracellular permeability and disrupting the tight junction proteins ZO-1 and occludin, while another study found that mCRP upregulates IL-8 and CCL2 gene expression and protein secretion, and also binds to complement factor H, which dampens its proinflammatory activity in these cells while increasing phagocytosis [110,111]. Finally, few early studies comparing pCRP and mCRP in animal models of cancer revealed that the conformation of CRP is a critical factor in eliciting the anti-metastatic effect. Also, these data indicated that the anti-tumor mCRP effect is specifically localized to the tissue-based pathology and does adversely affect other organs and tissues [76]. CRP was found to be non-toxic to normal cells but to have vast potential as a non-specific agent against various tumors, some of which are known to metastasize to various organs, but such analyses need to be repeated with certified and distinctively separated pCRP and mCRP reagents so that the actual CRP form as a biological modifier in cancer can be advanced [76]. Moreover, xenograft animal models of cancer (breast adenocarcinoma, melanoma, and others) have provided evidence of elevated necrosis and limited metastasis in the presence of the monomer, and this tumoricidal activity is ascribed to the promotion of the innate immune response, particularly macrophage activation; the promotion of ROS production and enhanced cytotoxic capacity could support tumor progression [102]. Nevertheless, such findings are contrasted to previous data, thus suggesting the differential modulation of these leukocyte subpopulations at the level of signaling. Figure 2 summarizes some potential roles of mCRP, as discussed in the current literature.

#### 3.2.2. The Pentameric or “Native” CRP

Regarding the bioactivity and the intensity of the anti-inflammatory activities of the pentameric form of CRP, the evidence in the current literature seems to be at odds. It was demonstrated that pCRP is the major form when the pH = 6.8 [112]. Nevertheless, its reported bioactivities include pathogen and necrotic cell opsonization possibly through their binding to complement factor H-related protein 4, the activation of the classical complement pathway and he regulation of the alternative pathway via their binding to factor H, the inhibition of Membrane Attack Complex (MAC) formation, the stimulation of leukocyte phagocytosis and oxidative metabolism, and also the neutralization of PAF-induced neutrophil degranulation and ROS production possibly via altering actin polymerization by elevating F-actin and reducing G-actin [76,113]. Yet, data on pCRP and the complement seem contradictory since other sources highlight that the pentamer appears to have no interaction with the complement or the regulatory complement factor H [114]. The pentamer scavenges for and facilitates the removal of nucleic acid and chromatin cell debris, and stimulates leukocyte phagocytosis and oxidative metabolism [76]. In addition, (native) CRP can affect IL-6-mediated inflammatory events by enabling the formation of the sIL-6R/IL-6 complex [115]. Moreover, an in vitro study demonstrated that human CRP directly contributes to adaptive immunity, with its native form specifically binding to human Jurkat T cells and to mouse naive CD4^+^ T cells, modulating their Th1 and Th2 responses and thus alleviating experimental autoimmune encephalomyelitis [116].

According to another in vitro study, compared to mCRP, the pentamer can possibly increase the levels and function of circulating endothelial progenitor cells, leading to their decreased viability and the induction of apoptosis. Also, it directly impairs endothelial tube formation, with the authors concluding that it exhibited a primarily non-inflammatory gene response [95]. The study also revealed a decreased number of acetylated LDL and ulex lectin double-positive cells after pCRP treatment [95]. Furthermore, the pentamer was found to suppress endothelium-dependent NO-mediated dilation by stimulating the p38 MAPK pathway and NADPH oxidase [117]. Old data demonstrated that pCRP, and not the monomer, enhanced the adhesive activity of neutrophil in a dose-dependent manner, but on the contrary, another in vitro study showed that the adhesion of neutrophils to endothelial cells was prevented through the shedding of L-selectin by CRP and its derived peptides. In this study, however, the exact form is absent [118,119]. Additionally, native CRP has been shown to inhibit the respiratory burst of neutrophils as demonstrated by the extracellular release of reactive O_2_ intermediates in response to a variety of agonists, such as fMLP, PAF, and PMA [26].

The literature data on atherosclerosis highlight that native CRP and native LDL interact with each other only if either one is immobilized, modified, or aggregated, thus raising the possibility that CRP and LDL can interact with each other under certain pathological conditions, while several studies suggest that pCRP binds to oxidized LDL through its phosphocholine moiety or via its amyloid-like structures, and sometimes when it is complexed with 2 glycoprotein I [120]. Additionally, interactions between native/aggregated CRP, LDL, and macrophages regarding their transformation to foam cells have been thoroughly investigated with no clear-cut overall conclusion, and similarly, it remains unclear whether both macrophages’ CD32 and LDL receptor CD36 participate if there is an effect of CRP on the uptake of LDL [120]. Apart from their possible interactions, a study showed that the treatment of human macrophages with native CRP increased LPL protein expression and secretion [121]. Moreover, the inflammatory properties of minimally modified LDL, which by itself induces macrophage spreading and impairs phagocytosis of apoptotic cells, have been found to be attenuated by pCRP, which inhibits monocyte and modified LDL interactions [122,123]. However, by binding to the phosphocholine of oxidized LDL, CRP induces the classical complement pathway, and activates and amplifies the innate immunity. Furthermore, a study showed that pentameric CRP significantly upregulated Il-8 at higher concentrations than those of mCRP, with PAI-1 levels being notably increased with native compared to mCRP, and while both decreased PGF1-α and inhibited eNOS activity, pCRP had such effects at lower concentrations. Therefore, the authors concluded that pCRP exerts more potent atherogenic effects in human aortic endothelial cells [124]. In atheroma, a CRP increase stimulates the induction of IL-6 by macrophages, suggesting that CRP may have a direct impact on IL-6 release; yet, an atherosclerosis model found that a combination of oxLDL along with the monomer and the pentamer decreases TNF-α and IL-6 production. Therefore, it is possible that the native form of CRP down-regulates the release of IL-6 by macrophages that were activated by both oxLDL and the monomer [125].

pCRP suppresses the adherence of platelets to neutrophils, whereas the monomer boosts these interactions [80]. This functional difference possibly occurs due to the two forms binding to different FcγRs involved in the signaling process; the monomer binds to the low-affinity immune complex binding IgG receptor called FcγRIIIb (CD16b) on neutrophils and FcγRIIIa (CD16a) on monocytes, whereas the pentameric CRP binds to the low-affinity IgG receptor FcγRIIa (CD32) and high-affinity IgG FcγRI (CD64), after which it can lead to the production of inflammatory cytokines. Concerning the apoptosis process, the pentamer binds to low-affinity CD32 and CD64, resulting in suppressed functional activities, degranulation, and the creation of superoxide by inducible respiratory burst, whereas the removal of pentamer-bound apoptotic monocytes and macrophages may be through FcγR-mediated phagocytosis. Although the literature data on the anti-atherogenic activities of CRP is conflicting, some studies conclude that when bound to platelets, native CRP can possibly eliminate the effects of physiological platelet agonists, inhibit platelet secretion (both platelet dense bodies and alpha granule constituents), reduce platelet aggregation, and potentially boost the anti-platelet effect of acetylsalicylic acid [26]. The pentamer exerts modulatory effects on monocytes by activating as well as limiting diapedesis in its early stages. Research has revealed that the pentameric CRP form down-regulates endothelial nitric oxide synthase (eNOS) in cardiovascular endothelial cells, thereby inhibiting NO production and angiogenesis and promoting atherogenesis while also upregulating the expression of adhesion molecules and inhibiting eNOS expression (CRP generally inhibits eNOS activation via insulin through blunting Ser1179 phosphorylation) [80].

**Figure 3:**
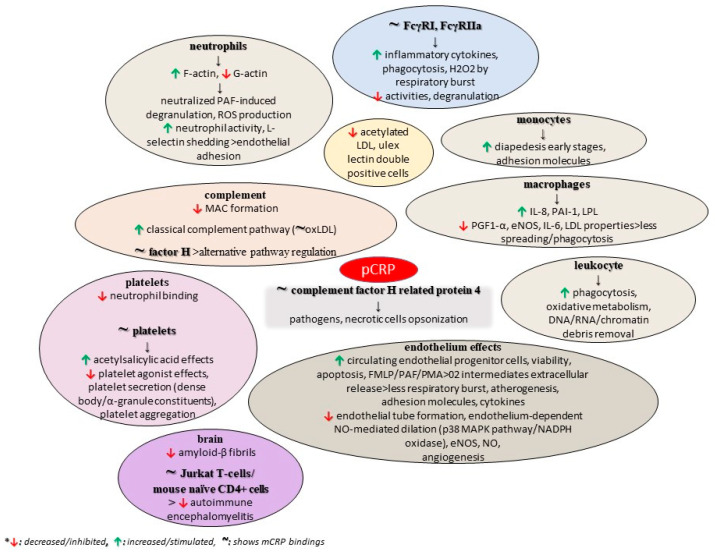
Potential functions of pCRP.

A study also found that structurally altered, and not wild-type, pentameric CRP inhibits the formation of amyloid-β fibrils, while the interaction between CRP mutants and Aβ prevented the formation of Aβ fibrils. These mutants were biochemically analyzed, revealing an altered topology of the Ca^2+^-binding site, thus proposing the role of this region of CRP in its binding to Aβ, with the authors ultimately concluding that CRP is a dual pattern recognition molecule and an antiamyloidogenic protein as well [126]. As previously discussed, the conformation of CRP is a critical factor for tumorigenesis, and mCRP is supposed to show anti-tumorigenic activities in contrast to the native form [76]. Figure 3 summarizes some potential roles of pCRP, as discussed in the current literature.

#### 3.2.3. No Precise Form of CRP

It seems difficult for one to draw clear conclusions regarding the definite functions of each CRP form since the current literature includes several published papers on CRP that do not distinguish between mCRP, pCRP, or other CRP forms. For example, apart from inhibiting podocyte autophagy through C3a/C3aR axis signaling, CRP bound to a multivalent ligand or in aggregated forms activates the classical C3 convertase pathway, which leads to the presentation of a ligand with opsonic complement fragments [67]. Nevertheless, the protein does not favor the formation of a C5 convertase; thus, CRP-initiated complement activation does not mediate acute inflammatory reactions and membrane damage [75]. CRP is stronger during the early classical pathway activation of C1, C4, and C2 because the ligand-bound interaction with C1q leads to C3 convertase formation (a molecule that can trigger apoptosis in human coronary vascular smooth muscle cells), resulting in the activation of C1–C4 convertases but with little stimulation of C5–C9 convertases [80,127]. CRP inhibits the alternative complement pathway by reducing C3 and C5 convertase actions and by inhibiting the complement amplification loop. This occurs through recruiting factor H to the cell surface and by preventing C5 convertase cleaving C5 to recruit neutrophils, and mostly through the prevention of MAC formation [80]. This limitation in the early components of the complement system mainly suggests the induction of phagocytic activity through deposition of C3b and iC3b. Also, the limited activation of the terminal MAC and C5a would be biologically favorable since such components can damage the host membrane [128]. After CRP titers elevate, the binding of C3b and C5b-9 to liposomes decreases, also possibly explaining C5–C9 sustenance by CRP during classical pathway activation [80]. Factor H-related protein 1, which promotes complement activation by the competitive inhibition of factor H binding to specific surfaces and immune proteins, was found at high concentrations to compete with factor H for CRP binding, revealing possible complement deregulation on this ligand [129]. CRP aids in the opsonization of apoptotic cells, but on the contrary, it was demonstrated in vitro that CRP can boost IgG-mediated cellular destruction via FcRs [80]. Importantly, CRP colocalized with the GADD153 gene product in atherosclerotic lesions, highlighting that CRP leads to the caspase cascade and apoptosis by promoting GADD153 gene expression [128,129]. Complexed CRP was previously shown to be an effective activator of the complement through C1q binding, and it may modulate pathophysiologic actions mediated via cryoglobulins in vivo since a previous case report described the formation of a complex between a monoclonal IgA with cryolabile properties and CRP. This is further supported by a study that provided the first evidence for CRP’s frequent occurrence in cryoglobulins (Cg) of all three types—according to Brouet’s classification [130]. On the contrary, CRP was found to upregulate factors inhibiting the complement in endothelial cells, thus proposing its potential protective atherogenic role again.

Apart from the complement pathways, the anti-inflammatory properties of CRP may be linked to its ability to enhance the expression of Il-1 receptor antagonist in human peripheral blood mononuclear cells [26]. Not only can CRP induce the expression of Il-1 receptor antagonist in peripheral mononuclear cells (to a greater extent than stimulating Il-1 production) and the release of Il-18, but it can also down-regulate IFN-γ production [36]. Furthermore, some literature data have revealed a correlation between CRP and IL-6 increase with IL-6 inducing the CRP gene. Additionally, CRP can alter the cytokine profile in mouse macrophages by enhancing the secretion of the anti-inflammatory cytokine Il-10 and down-regulating the production of Il-12 [26]. An in vitro study showed that CRP significantly upregulated the surface expression of FcγRs, mainly CD32, and also CD64 on human aortic endothelial cells, while-it-its-binding-to-and-internalization-of-biotinylated CRP induced an increase in Il-8, intercellular adhesion molecule 1, and VECAM-1 and a decrease in eNOS and prostacyclin [131]. Furthermore, CRP can enhance Il-8 production and increase Il-8 mRNA expression in a CRP dose-dependent manner. Furthermore, it promotes Il-8 production through stimulating ERK, p38 MAPK, and JNK pathways, but a study showed that Il-8 results in CRP production by hepatocytes, providing a potential feedback loop [132,133]. The activation of focal FAK, paxillin, and ERKs can be mediated via the binding of CRP with both FcγRs and integrin a2 as well. Moreover, in hepatocytes, TNF-α induces a dose-dependent CRP secretion that corresponds to CRP mRNA elevation, and conversely, increased CRP levels in atheroma induces Il-1β, IL-6, and TNF-α production by macrophages [80]. Yet, CRP can inhibit TNF-α production with a possible negative feedback mechanism, while high CRP titers suppress further CRP activation by decreasing the production of TNF-α [134]. In a prospective study on multiple trauma patients, IL-1β plasma titers negatively correlated with preceding CRP levels, while inflammasome-independent cytokines IL-6, IL-18, and TNF-α showed a positive correlation. Finally, the authors concluded that phosphocholine-laden CRP is an unconventional nicotinic agonist that can possibly inhibit ATP-induced inflammasome stimulation and could protect against trauma-associated sterile inflammation [135]. CRP triggers metabotropic functions at nAChRs containing subunits α7, α9, and α10, and down-regulates the function of ATP-sensitive P2X7 receptors in monocytic cells. Importantly, CRP does not activate ion currents at conventional nAChRs, which suggests that CRP is a possible nicotinic agonist regulating innate immunity without entailing the risk of adverse events in the nervous system [135]. It has also been shown that the infusion of recombinant human CRP into healthy volunteers leads to a substantial increase in serum IL-6 and IL-8, serum amyloid A, serum phospholipase A2, prothrombin 1 and 2, D-dimer, and PAI-1, leading to an overall activation of inflammation and coagulation, as well [136]. Also, it can interfere with Activated Partial Thromboplastin Time (APTT), leading to prolonged clotting times. Yet, the possibility of the injected recombinant CRP dissociating in situ cannot be excluded; thus, the interpretation of such data seems ambiguous. On the contrary, a study concluded that the modified forms of CRP inhibit chemotaxis, a function similar to native CRP, but that the monomer and the native molecule interact with and bind to different neutrophil receptors [137]. Moreover, CRP 77–82, 83–90, and 201–206 residues can act additively to affect degranulation and inhibit superoxide production from activated neutrophils at 50 μΜ, with the latter two inhibiting neutrophil chemotaxis [26]. An interesting study demonstrated that CRP bound FcαRI on neutrophils and macrophages through its effector face, a region overlapping with complement C1q/FcγR binding sites, while its cross-linking of FcαRI resulted in ERK phosphorylation, cytokine production, and FcαRI-transfected RBL cell degranulation, and also CRP-induced FcαRI surface expression, phagocytosis, and TNF-α secretion in neutrophils [138]. Furthermore, heat-aggregated CRP activates platelet aggregation, secretion, and thromboxane A2 generation, similar to heat-aggregated IgG [26]. CRP can act as a substrate for membrane-associated neutrophil serine protease, which cannot be upregulated [75].

CRP can negatively modulate NO production and can lead to some other procoagulant effects, such as reducing PGI2 release, diminishing fibrinolysis, the release of tissue factor, and the increase in thrombocyte adhesiveness; however, the exact form for such effects is unknown based on the data presented in [139]. CRP has also been found to inhibit both the stimulation of nitric oxide release through the down-regulation of endothelial nitric oxide synthase and its insulin activation via the immunoreceptor tyrosine-based inhibition motif of FcγRIIB and SHIP-1. In contrast, it upregulates angiotensin receptor-1 protein expression and increases its number on vascular smooth muscle cells, and promotes vascular smooth muscle migration and proliferation in vitro [75,140]. An in vitro study in neonatal rat cardiomyocytes showed that CRP interferes with the desensitization of agonistic stimulated GPCRs and must be considered as a novel regulator of adrenergic, angiotensin I, and endothelin receptors, although it is not known if it directly interacts with the respective GPCRs or other receptors on the cardiomyocytes [141]. Nevertheless, another in vitro study in rat vascular smooth muscle cells revealed that CRP increased mRNA levels and the protein expressions of VEGF-A and inducible nitric oxide synthase, and boosted NO secretion in the medium, but it also hindered the nuclear translocation of glucocorticoid receptor and diminished its mRNA level and protein phosphorylation in these cells [142]. In this study, TLR4 small-interfering RNA significantly reversed CRP’s effects, suggesting that CRP can induce inflammatory responses via TLR4; yet, the exact form of CRP that can lead to such effects was not reported (or not studied). CRP can result in time- and dose-dependent increases in PAI-1 concentration and activity along with elevated intracellular PAI-1 mRNA and proteinic concentrations, and can also enhance the proinflammatory effects promoted by angiotensin II as well as inhibit the release of a natural anticoagulant—a tissue factor pathway inhibitor—from human endothelial cells, thus indicating its possible pro-thrombotic role [26]. Tissue factor is also stimulated by CRP on peripheral blood monocytes. CRP can affect plaque remodeling by activating matrix metalloproteinases and inhibiting their inhibitor, and can result in oxidative stress by increased ROS synthesis in the vascular wall. It can also impair vasodilatation, damage the glycocalyx, and increase endothelin-1 and vWb, thus resulting in general endothelial dysfunction [140]. In addition, another in vitro study showed that CRP significantly attenuated the flow-mediated activation of Akt, which mostly controls endothelial cell survival pathways, and in human mononuclear cells, CRP resulted in the production of TNF-α, IL-1β, and matrix metalloproteinase-9 in a concentration-dependent manner that was significantly inhibited by function-blocking antibodies to TNF-alpha, IL-1beta, and FcgammaRIIA [143]. CRP can also increase blood–brain barrier paracellular permeability and enter the brain parenchyma in mice with adult-onset obesity in a dose-dependent manner [144]. Studies on LDL/CRP demonstrate that native LDL coincubated with CRP was taken up by macrophages via micropinocytosis, while CRP/LDL coincubate uptake was mediated by the CRP receptor CD32 [145]. The authors concluded that foam cell formation in human atherogenesis can partially be caused by the uptake of CRP-opsonized native LDL.

Another important fact for CRP—even when its precise form is not known—is that it can be rapidly and actively transported into the cell nuclei due to the presence of a nuclear localization sequence, and as it binds strongly to snRNPs both in vitro and in vivo, it is possibly involved in the clearance of these critical autoantigens; however, CRP was found to be protective against the formation of autoimmunity in mice, and such data suggest that CRP is perhaps capable of monitoring the expression of certain genes [128]. However, pCRP was related to its binding to nuclear structures at physiological ionic strength, such as nucleosome core particles and extrachromosomal constituents, including snRNPs. Furthermore, CRP upregulates p53 in monocytes and influences their cycle kinetics through CD32, inducing apoptosis by G2/M through the upregulation of B-cell translocation gene 2 expression; CD32 receptors are expressed in a subset of monocytes that polarize to proinflammatory macrophages and trigger apoptotic signals, suggesting that CRP can dampen macrophage-driven proinflammatory responses by inducing apoptosis [80,146,147]. Nevertheless, CRP does not opsonize early apoptotic neutrophils, but rather binds only intracellular structures in membrane-permeable late apoptotic cells and has no impact on their phagocytosis by macrophages [148]. An in vitro study demonstrated that CRP upregulates M-CSF release from human aortic endothelial cells and increases macrophage proliferation, and these effects appeared to be mediated through the activation of NF-κB via CD32 and CD64, thus providing further evidence for its proatherogenic role [149]. Additional in vitro studies on cardiomyocytes suggest that CRP notably upregulated NCX1 expression and elevated intracellular calcium concentration through the NF-κB pathway, highlighting its potential role in arrhythmias. Another in vitro study revealed that both the phosphorylation and translocation of PKC-β2 to the membrane were inhibited by a certain CRP dose, that the translocation to the membrane and the serine-phosphorylation of the major cytosolic p47-*phox* component of the NADPH oxidase complex was inhibited by CRP, and also that CRP inhibited the membrane localization of activated Rac2, the small G protein modulator of the assembly of the oxidase components in stimulated neutrophils, and the cytoskeleton during chemotactic movement [150]. These findings imply that CRP could play a crucial protective role during the early phases of the inflammatory reaction [151]. Additionally, CRP down-regulates TRAIL expression in peripheral monocytes via an Egr-1-dependent pathway, whereas it upregulates the whole blood expression of the major cell surface inhibitor of MAC, as well as the Receptor for Advanced Glycation End Products (RAGE) expression while modifying antioxidant defenses in rat endothelial progenitor cells [152,153].

Moreover, it was established that CRP exacerbates acute kidney injury in mice, and this was associated with the heightened renal accumulation of myeloid-derived cells with suppressor functions, with a research study revealing CRP’s ability to expand and trigger the increased FcγRIIb-independent production of iROS in these cells, as well as its ability to FcγRIIb-dependently enhance the T cell-suppressive action [154]. CRP also can promote the osteo-/chondrogenic transdifferentiation of vascular smooth muscle cells via mechanisms involving the Fc fragment of FcγRIIa (this can blunt CRP’s procalcific effects) and the dependent stimulation of oxidative stress. Another study showed that CRP can suppress the development, maturation, and function of dendritic cells, implicating this protein in the maintenance of peripheral T cell tolerance [155]. Even if previous data suggest the tumoricidal activity of mCRP and its potential induction in leukemia cell lines in vitro, there exists a study elucidating the critical link between CRP, integrin α2, and FcγRI pathways in MCF10A breast cells and MDA-MB-231 triple-negative human breast cancer cells, thus providing useful evidence on the CRP-induced aggressiveness of breast cells in inflammatory microenvironments [156].

It should be highlighted that CRP is differentially glycosylated in multifarious pathological conditions, and such glycosylated CRP variants can regulate their binding activity with various ligands (and even protect the clearance of damaged erythrocytes in various diseases) [157]. Finally, concerning the other forms of CRP, the current literature does not directly reveal the precise functions of the pentamer’s peptide or subunits (in forms of 2–4 subunits complexes). Furthermore, decameric CRP is not known for any specific role despite its potential action in host defense or apoptotic cell clearance; the rapid equilibrium amongst CRP pentamers and decamers provides some way to eliminate the non-specific binding of other proteins to CRP, and in this way, the integrity of CRP—when abundant in plasma—is sustained [158]. This fact seems functionally critical since plasma contains dozens of various proteins, but more research is still required to reveal if pentamer–decamer exchanges take place in blood. In this way, it could be assumed that decamers can affect interactions between CRP and its ligands, the ones that are supposed to bind to the A face of the protein, and, conversely, the interactions amongst pentameric CRP and its ligands, i.e., factor H could also lessen decamer formation [158].

### 3.3. Function of Autoantibodies against C-Reactive Protein

In 1985, Frank A. Robey and colleagues reported anti-CRP antibodies in one out of eight Systemic Lupus Erythematosus (SLE) cases and a reduced CRP ability to solubilize chromatin in certain SLE cases, whereas other scientists reported a high frequency of antibodies to cryptic epitopes of CRP in patients suffering from toxic oil syndrome—a condition resembling SLE. Afterwards, significant anti-mCRP IgG titers were reported in SLE cases, along with a lower prevalence of Subacute Cutaneous Lupus Erythematosus (SCLE) and Primary Biliary Cirrhosis (PBC) [159]. Anti-mCRP titers were also observed in the sera of patients with various rheumatological conditions (including systemic rheumatic diseases), and primary antiphospholipid syndrome, in which there were no correlations with CRP titers [159]. Interestingly, a study trying to reveal such antibodies in some other systemic autoimmune diseases revealed that anti-CRP antibodies were evident in more than half of SLE and in less than half of SCLE patients, whereas Systemic Scleroderma (SS) cases were positive. The authors concluded that chronic inflammatory tissue injury in systemic autoimmune disease can possibly increase the presentation of cryptic CRP epitopes to the threshold required for the activation of T cells [160]. However, it has conceivably been proposed that SLE should be regarded as a disease with dysregulated apoptosis and/or defective apoptotic material clearance, yielding elevated circulating autoantigen titers and an autoantigenic overload, thereby resulting in a ‘mission impossible’ for the body’s waste disposal system. Structurally modified autoantigens on apoptotic blebs could ultimately be presented to T lymphocytes, causing B cell activation and the creation of autoantibodies, and this, along with subsequent IC formation/deposition, stimulates inflammatory tissue destruction along with apoptosis, thus giving rise to a vicious pathogenic cycle [161].

Antibodies against the monomeric form of CRP, which are supposed to target the autoantigens expressed both in kidney tubules and uveal cells, were found to ameliorate arthritis and nephritis in mice, whereas a case report on Tubulointerstitial Nephritis and Uveitis (TINU) syndrome discloses their higher titers during flares (that were found to be colocalized in renal and ocular tissues in another study), with the authors concluding its pathogenic role on the disease [161,162,163]. One could argue that such controversial data maybe indicate the substantial differences present between in vitro and in vivo research or a potential misdiagnosis. Another case report concludes that IgG4-related autoimmune disease should be considered for diagnosing cases with tubulointerstitial nephritis and multisystem involvement, and that mCRP autoantibodies could be related to IgG4-related tubulointerstitial nephritis and might be useful as an indicator of this condition [164]. However, 35–47 and 199–206 are predominant epitopes in mCRP recognized by autoantibodies, with the former being critical for lupus nephritis; anti-35–47 mCRP IgG inhibit the interactions between mCRP and complement factor H and are associated with renal injury and the prognosis of the disease [70]. A study on three lupus nephritis patients demonstrated that anti-mCRP antibodies purified from IgG fractions by affinity chromatography could significantly inhibit the binding of mCRP with C1q or factor H, and eliminate late apoptotic cell clearance enhanced by mCRP, suggesting that anti-mCRP antibodies from lupus nephritis patients might be pathogenic in SLE and lupus nephritis via interfering with the biofunctions of mCRP [165]. Significant anti-mCRP IgG titers were also demonstrated to be present in another autoinflammatory disorder with unknown etiology, referred to Periodic Fever, Aphthous Stomatitis, Pharyngitis, Cervical Adenitis (PFAPA) syndrome [166].

Generally, autoantibodies against acute-phase proteins like CRP might be generated by different mechanisms, including molecular mimicry; these immunoglobulins can also just be innocent bystanders. CRP transitional conformations resulting in neoepitopes can include proteolytic cleavage following activation or following its binding to another protein. Therefore, it is possible that anti-CRP antibodies are created as a part of the autoantibody response to CRP’s structurally modified forms, maybe evolving from binding to cells, apoptotic structures, proteins, or immune complexes [167,168]. If hidden epitopes on conformationally changed antigens or post-translationally modified autoantigens’ neoepitopes (such as glycosylation or citrullination) are exposed, this can stimulate the production of various autoantibodies. Also, the elevated immunogenicity of modified autoantigens is highly supported by data from mice experiments [159]. It is evident that mCRP, when bound to cellular surfaces/liposomes, could be a target for anti-CRP autoantibodies. The monomers’ expression on human peripheral blood lymphocytes in parallel with the increased lymphocyte apoptosis in SLE patients shows an inverse relation between high anti-CRP IgG titers and lymphopenia, which possibly results from the opsonization of lymphocytes expressing mCRP on their cell surfaces, thus increasing the elimination of circulating lymphocytes through the reticuloendothelial system [159]. Speculatively, anti-CRP antibodies may interfere with the physiological mCRP-mediated removal of immune complexes and/or various nuclear constituents, and additionally, C1q-binding CRP has complemented activating functions that also promote the clearance of immune complexes [159]. Furthermore, possible post-translational glycosylation could be related to CRP’s circulating clearance as well as the generation of anti-CRP antibodies. It has been shown that CRP molecules in various conditions—including SLE—have differences in carbohydrate content and amino acid sequences [169,170]. Some further potential anti-CRP pathogenic functions may include their interaction with surface-bound CRP on cells or liposomes and tissue surfaces; the exposed monomer on surfaces of apoptotic residues, i.e., in the renal glomeruli, might be a target for circulating anti-CRP antibodies in situ, which could set or amplify inflammation in target organs [159]. Other data indicate the binding of CRP and other opsonins to the surface of secondary necrotic cells, which interact with and are sensitized by anti-dsDNA and anti-CRP autoantibodies. The complexes of such cells and immunoglobulins were cleared by macrophages in vitro and induced a pro-inflammatory cytokine cascade, suggesting that anti-CRP and CRP, along with these cells, create a ternary pyrogen endowed with strong proinflammatory capabilities that aid in the maintenance of chronic inflammation [171].

High anti-CRP IgG levels were found also in all hepatitis C patients, whereas only a few autoimmune hepatitis and Non-alcoholic Fatty Liver Disease (NAFLD) sera were positive. Also, anti-CRP was not associated with a response to interferon-based administration or cirrhosis development nor related to liver-related mortality [172]. Apart from the anti-mCRP autoantibodies found in Periodic Fever, Aphthous Stomatitis, Pharyngitis and Cervical Adenopathy (PFAPA) syndrome, the significantly lower anti-mCRP antibody titers found in acute coronary syndrome (ACS) patients compared to healthy controls. This led the authors to suppose that it is plausible that ruptured plaques and inflamed tissue are likely to be more prone to opsonization by mCRP, thus leading to anti-CRP consumption, and they further hypothesize that surface-bound anti-CRP could therefore boost local plaques’ inflammation [173,174]. Importantly, it was stated that most autologous ligands recognized by CRP overlaps with those of antiphospholipid autoantibodies that are related to premature cardiovascular disease in autoimmune syndromes [65]. Yet, they could simply be lower due to immune complexes with their target or because of the previously discussed reasons. Thus, related studies need to re-evaluate such data and further discuss their evidence.

### 3.4. Receptors and Ligands of C-Reactive Protein

It was discussed that each protomer has a unique ligand binding site, and all five binding sites are on the same face of pCRP. Due to the pentameric arrangement of its binding sites, a high repeat number of any ligand in a large array, as exist on a pathogen’s surface, can bind to CRP with high avidity. The binding of CRP to cellular FcγRs that occurs in five C1q/FcγR binding sites on the other face of CRP is supposed to account for its opsonizing properties, resulting in a response from phagocytic cells [156]. Old scientific data reveal that the major receptor for CRP on leukocytes is FcγII; generally, it is believed that mCRP binds to low-affinity FcγRIIIa (CD16a) on monocytes and to low-affinity IgG FcγRIIIb (CD16b) on neutrophils, whereas pCRP binds to the stimulatory low-affinity IgG receptor FcγRIIa (CD32) and to the inhibitory receptor FcγRIIb, thus blocking activating signals, and to some extent to the stimulatory high-affinity IgG FcγRI (CD64). with a 3-fold higher affinity than IgG. Particularly, a recent study showed that CRP binding to FcγRIIa on human monocytes and neutrophils is allele-specific [80,159,175,176]. Indeed, a study concluded that mCRP and pCRP bind to different receptors on human neutrophils, whereas other data indicate that there is lack of specific CRP receptors on white blood cells [177,178]. Yet, very old data indicate that CRP does not bind to the IgG FcγRs of monocytes, but rather that they have other distinct receptors on their surface [179]. It is believed that the binding of CRP to FcγR is supposed to show similar effects as the binding of IgG. FcγR-containing immunoreceptor tyrosine-based activation motifs (ITAMs), including FcγRI, FcγRIIa/c, and FcγRIIIa, are stimulated by clustering on the cell surface due to ligand binding, and the subsequent phosphorylation of the two tyrosines in the ITAM by Src-related tyrosine kinases (like Lyn, Fgr, and Hck) results in the recruitment of Src homology 2-containing molecules (like Syk tyrosine kinase), which leads to the following cascade: firstly, the phosphorylation of PI3K with the generation of PI(3,4,5)P_3_, which activates downstream signaling events, including the phosphorylation of PLCγ2, thereby producing DAG, which activates PKC, subsequently activating p38 transcription factor and calcium mobilization through IP_3_; and secondly, the activation of Raf that binds Ras also phosphorylates MEK, which phosphorylates ERK, subsequently stimulating transcription from promoters regulated by c-Myc, Ets, CREB, NF-κB, and AP-1 [179]. ERK can also be stimulated by the Rho family GTPases Rac/Cdc42, which are also activated after FcγR crosslinking and are critical to the control of phagocytosis [180]. Finally, regarding its potential receptors, CRP can also bind to αIIbβ3 receptor on platelets, FcαRI in neutrophils and macrophages, lectin-like oxidized LDL receptor (LOX-1) in macrophages, and TLR4 in vascular smooth muscle cells; it also stimulates nAChRs [135,138,142,181]. Yet, the molecular mechanism of the downstream effect of the binding of CRP to Fcγs is not that clear and needs to be further elucidated. In addition, the monomeric form of CRP binds and neutralizes the receptor activator of NF-κB ligand and also binds to leptin receptor [98,102].

It is well known that in inflammatory microenvironments with acidic conditions, native CRP dissociates into CRP monomers, which are capable of binding to IgG-containing immune complexes, and generally, CRP can be recognized by IgG as well as Fc*γ* receptors, which are cell surface glycoproteins found on numerous cells, such as macrophages, mast cells, platelets, and leukocytes, of both lymphoid lineage and myeloid lineage. Following their binding, this protein can opsonize pathogenic agents and particulate self and foreign antigens [35]. Importantly, at physiological ionic strength, pCRP can bind to nuclear structures such as nucleosome core particles and extrachromosomal constituents, including snRNPs [159]. Old data reveal that CRP bound to the D protein of Sm and the 70 kDa protein of snRNPs, and that these antigens are major targets of autoantibodies in SLE cases [182]. Indeed, several nuclear antigens that bind to CRP are the same as those targeted by Antinuclear Antibodies (ANA) seen in the sera of SLE patients or others with systemic inflammatory rheumatic diseases. It is conceivable that CRP, via FcγR-mediated uptake in phagocytes, mediates the clearance of circulating nucleosomes and apoptotic blebs on which nuclear antigens are exposed, thus limiting the contact of such autoantigens with the adaptive immune system [159].

There exist multifarious data in the current literature that do not distinguish the interaction between phosphocholine (the parent compound of phosphorycholine) and CRP from the interaction between phosphorycholine and CRP, thus making it difficult for someone to determine to which ligand CRP bind more effectively. Nevertheless, not only is phosphocholine a component of various prokaryotes, but it is also a constituent of sphingomyelin and phosphatidylcholine (which again can bind to CRP) found in eukaryotic cell and extracellular membranes. However, CRP cannot have direct access to the head groups of these phospholipids in this manner; it can bind to them only if cells are damaged and apoptotic. PLA2 hydrolyzes phosphatidylcholine to biologically active lysophosphatidylcholine, which is also a pCRP ligand [83]. Phosphorycholine is identified on some Gram-positive bacteria, including Clostridium spp., *Lactococcus* spp., and *Bacillus* spp., on some Gram-negative bacteria, including *Haemophilus influenza*, *Neisseria meningitides*, and N. gonorrhoeae, and some other pathogens, including Mycoplasma and *Streptococcus pneumonia*. Notably, phosphorycholine and lyso-phosphorycholine comprise about a third of the polar lipids of the sporozoan Pneumocystis carinii carinii detected in infected rat lungs [183]. Such interactions with phosphorycholine have a broad range of pH (6–8). Moreover, further independent analyses have now established that the pentamer converts into its monomeric form when in proximity to apolar lipid membranous zones, particularly when membrane lipids are activated into lyso-lipids (i.e., monoacylglycerophosphatidyl choline), a fact which better allows for the pentamer to bind to its phosphocholine ligand [75].

CRP can also interact with and bind to phosphocholine esters, phosphorylated carbohydrates, polycations, galactans (that have lectin-like characteristics) and lecithin (phosphatidylcholine) [77]. Generally, apart from phosphocholine, CRP has the ability to interact with several other autologous ligands, such as modified and unmodified plasma lipoproteins, damaged cell membranes, different phospholipids and related compounds, apoptotic cells, and small nuclear ribonucleoprotein particles, as well as with other macromolecular ligands, including phosphoethanolamine, chromatin, histones (which have a stronger binding to H1 and H2A and relatively weaker binding to H2B, H3, and H4, but it does not bind to naked DNA), M-ficolin, integrin a2, fibronectin, laminin, and polycations [35,39,77,184]. Intriguingly, most of the spectrum of autologous ligands recognized by CRP overlaps with that of antiphospholipid autoantibodies that are related to premature cardiovascular disease in autoimmune syndromes [65]. An in vitro study revealed that Gal6P-BSA and Galbeta3GalNAc-BSA could bind to neo-CRP (mCRP) and pCRP, the former with/without calcium and the latter only in the absence of calcium, and also that phosphate-containing ligands can be bound with/without calcium, yet the binding is much stronger in the presence of calcium. Importantly, some cross-inhibition studies further proved that binding sites of phosphorycholine and sugar are contiguous, and that ligand-binding mCRP conformation seems more fragile than pCRP in acidic media (pH < 6) [185]. Another in vitro study concluded that the binding of sugar phosphates by neo-CRP (mCRP) has notably less stringent requirements compared to the pentamer; however, this study is an in vitro study, and it does not consider the overall pathogenic condition of a patient [186]. Furthermore, some extrinsic ligands that bind to CRP include several glycan, phospholipid, phosphorylated carbohydrates, and other constituents of microorganisms, such as the capsular and somatic components of bacteria, fungi, and parasites, as well as some plant products [69,187]. In acidic inflammatory microenvironments that trigger conformational modifications in CRP, the protein has two extra binding sites within its intersubunit regions, specifically in the loop containing residues 115–123. Despite this, the specific implicated amino acids are still unelucidated; the first site binds to factor H, and the second site may bind to any structurally changed protein regardless of its identity [35]. Before dissociating into the monomeric form, the pentameric CRP can bind to human complement factor H-related proteins 1 and 4 (the latter recruits pCRP) [128,188]. The release of each subunit permits the exposure of previously hidden epitopes that show distinct antigenic features and also activate platelets, polymorphonuclear leukocytes, monocytes, lipoproteins, and the complement system in vitro [189]. Another in vitro study found that an inflammatory acidic pH ranging from 4.6 to 5.2 did not monomerize CRP, but rather modified its pentameric structure; the pentamer could bind to complement factor H, oxidized LDL, complement C3b, amyloid β, and BSA immobilized on microtiter plates, whereas CRP did not bind to any of these biomolecules under neutral conditions [190]. Another in vitro study demonstrated that the modification of non-esterified cholesterol in LDL by cholesterol oxidase reduced the binding of CRP to LDL, thereby increasing the rate of CRP binding to purified non-esterified cholesterol, and the binding was calcium-dependent and could be outcompeted with phosphocholine [191]. Such evidence suggests that CRP can bind to modified lipoproteins, particularly to the non-esterified cholesterol on their surfaces. CRP can bind to cholesterol, and this binding is facilitated by the phosphorylcholine-binding site of CRP and the 3beta-hydroxyl group of cholesterol [192].

It was previously discussed that CRP may affect antigen presentation, and also that under certain circumstances, such as under an acidic pH in vitro, CRP adopts a different pentameric configuration that exposes a hidden ligand binding site for non-phosphocholine ligands, which also enables CRP to bind to immobilized, denatured, and aggregated proteins, regardless of the identity of the native biomolecule [39,83,193]. It is well-known that the ligand recognition function of CRP depends on the presence of an acidic inflammatory microenvironment, such as H_2_O_2_, according to in vitro studies. Additionally, this specific CRP ligand binding, in its acidic pH-induced pentameric state, has implications for toxic conditions involving protein misfolding in acidic environments and favors CRP conservation throughout evolution [189]. Generally, transitional changes of the CRP structure lead to the exposure of proinflammatory binding sites, as well as neoepitopes [194]. Moreover, it was revealed that conformational CRP changes can enable the protein to bind to atherogenic lipoproteins, thus reducing atherosclerosis [195]. It was also discussed that differentially glycosylated CRP variants can regulate their binding activity with various ligands in certain pathological conditions [195]. However, the current literature does not reveal if both CRP forms bind to most previously reported ligands, or if there is any specific binding for each form with targeted ligand, like the variance of each form with FcRs’ affinities. Additionally, it should be highlighted that some CRP mutants maybe not bind to commonly known ligands or/and receptors, and currently, there is a study revealing a CRP mutant that does not bind to phosphocholine nor pneumococcal c-polysaccharide [196]. In this manner and inversely, it is conceivable that CRP may be unable to interact or bind to mutated biomolecules that were its ligands or/receptors in their Mortprevious wild-type form.

**Figure 4:**
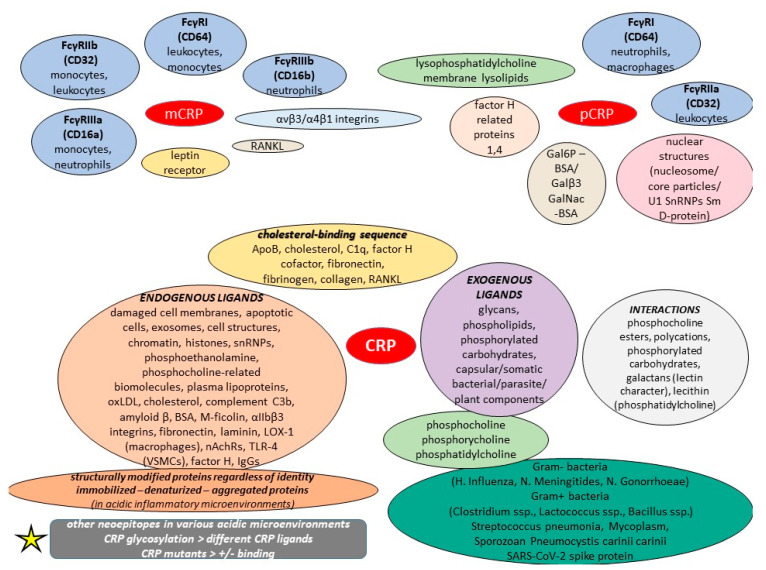
Potential CRP receptors and ligands.

Yet, it should be noted that CRP binds to such ligands or/and receptors since they are possibly present in certain microenvironments. This largely depends on their concentrations and other bound-to-bound electrochemical forces that affect such interactions and further binding. This means that CRP may bind to some ligands/receptors with no subsequent realistic consequences since the higher the concentrations, the more likely the interactions actually are. Figure 4 summarizes the potential receptors and ligands of CRP.

## 4. Current Evidence on C-Reactive Protein and Potential Conditions

### 4.1. C-Reactive Protein and Physiological Disorders

It is well established that in healthy young adult volunteer blood donors, the CRP median concentration is 0.8 mg/L, the 90th centile is 3.0 mg/L, and the 99th centile is 10 mg/L. But, following an acute-phase stimulus, values may increase from less than 50 μg/L to more than 500 mg/L, which is actually a 10,000-fold increase; generally, a CRP of more than 50 mg/dL is supposed to be a severe elevation [26,64]. In 1965, Gotschlich and Edelman reported for the first time that the CRP that was purified from serum was mainly in its pentameric form, and nowadays, most laboratories measure this form since no antibodies specific for the monomer are currently commercially available [26,58]. In this manner, only the pCRP is measured and interpreted as a diagnostic maker in blood specimens. The level of CRP is modified in various conditions, and even if the increase in CRP is non-specific, the quantum and the pattern of rise will help deduce the diagnosis of certain medical conditions [77]. Generally, a “diagnostic” biomarker either identifies or verifies the existence of an illness or a condition of interest, or it detects a person who has a certain medical condition, whereas a “monitoring” biomarker is the one that can be repeatedly evaluated to determine a disease’s progression, to look for signs/symptoms of exposure to a medical product/environmental agent, or to detect a medical product’s/biological agent’s results. The current literature reveals that CRP has been reported as both a possible diagnostic and potential monitoring biomarker in certain medical conditions. On the whole, and as it was previously discussed, normal human serum contains CRP concentrations of <10 mg/L, which can be elevated with age with no significant differences between men and women and with the upper reference limit reaching as high as 37 mg/L. Slightly higher levels can be found during late pregnancy, and mild inflammation and viral infections can produce elevations ranging among 10–40 mg/L, whereas moderate inflammation and pathogenic infections may cause increases in the range of 40–200 mg/L. Concentrations higher than 200 mg/L have been reported in severe bacterial infections, and concentrations even higher than 1000 mg/L can be seen in severe tissue injuries, such as burns [28,35,39,45,58,77].

In order to evaluate the CRP diagnostic significance, the US Department of Health and Human Services guidelines has define a distinction between “conventional CRP” values and “high sensitivity CRP (hsCRP)” values, whereas the FDA guidelines only include conventional CRP levels, defined as being >10 μg/mL, and adds that even if hsCRP levels (i.e., <10 μg/mL) are an area of great research interest, such values are non-specific and must be interpreted in combination with a full clinical evaluation since their clinical benefit is uncertain [63]. The term “high-sensitivity” or “highly sensitive” CRP, abbreviated as hsCRP, has been widely adopted in the current literature. It depicts CRP’s measurement in serum or plasma specimens with sufficiently sensitive assays to quantify CRP throughout its normal range compared to older, less sensitive commercial methods with detection limits in the range 2–10 mg/L that were more suitable for the measurement of CRP acute-phase responses rather than baseline values [197]. It is worth to highlight that the analyte designated as hsCRP is simply the already-known CRP, i.e., it is not anything new or different, or a novel analyte with any special relationship to Cardiovascular Diseases (CVDs) [197]. CRP is the equally exquisitely sensitive and non-specific for all potential systemic marker of various conditions, which has been thoroughly studied and utilized clinically for over 75 years [197]. This section discusses only the medical conditions in which CRP is somewhat elevated—as seen in various literature data.

#### 4.1.1. Cardiological Disorders

The association between CRP and cardiovascular risk is notably driven by systemic inflammation, but CRP is unlikely to directly contribute to CVDs as a pathogenic factor, and similar results were evident from recent Mendelian randomization studies [77]. Although there exists a debate regarding the precise physiologic role of CRP, hsCRP is supposed to be a prognostic marker of cardiovascular risk. By way of globally available molecular diagnostic assays, CRP levels of <1, 1 to <3 and ≥3 mg/L have been defined as low-, moderate-, and high-risk groups for future cardiovascular events, with individuals with an LDL cholesterol <130 mg/dL and a CRP level of 3 mg/L are considered a high-risk group [77]. A large meta-analysis on non-CVD data revealed that increased CRP worsened the 10-year prognosis of cardiovascular risk, and additionally, a meta-analysis demonstrated a link between increased CRP and higher cardiovascular risk [83]. An interesting meta-analysis exploring studies published from 1966 to 2007 revealed that the relative cardiovascular risk is 1.58-fold higher in cases with CRP titers of more than 3 mg/L than in those with less than 1 mg/L [198]. Nevertheless, both very low (<0.5 mg/L) and very high (>10 mg/L) hsCRP levels provide crucial prognostic information on cardiovascular risk, and hsCRP is clinically useful for risk prediction across a full range of values and across a full range of Framingham risk scores [199]. Prospective epidemiologic studies with follow-up periods (3–20 years) have demonstrated that a sole hsCRP measurement is a strong predictor of MI or coronary heart disease (CHD) mortality, congestive/systolic heart failure, congenital heart disease, atrial fibrillation and its recurrence, sudden cardiac death in people with no CVD history, and even cardiac sarcoidosis [12,114,200,201].

Low to high hsCRP levels have shown a linear trend association with systolic Heart Failure (HF), and hsCRP > 10 mg/L was independently associated with systemic HF. Even if hsCRP is increased in both systemic and diastolic HF, thus predicting survival, a study found that hsCRP independently predicted hospitalizations in cases with systolic, but not diastolic, HF, for which more prognostic studies are needed. Nevertheless, another study concluded that there was no association between plasma CRP values at admission in elderly acute HF subjects and subsequent higher 3-month mortality or readmission risks, and also that this marker can be critical in acute respiratory hypoxemic syndrome due to HF [202,203,204,205]. CRP as a marker for post-infract heart failure has also been reported; however, more prospective studies are still needed not only to explore the utility and dynamicity of CRP in heart failure with preserved ejection fraction, but also to determine if risk stratification algorithms with CRP really provide benefits in improving patient prognosis [206,207]. With the exception of Brugada syndrome, hsCRP can show symptomatic stages. Additionally, a study concluded that frailty is related to a higher hsCRP level, for which it seems to be a promising biomarker in heart failure [208]. Older data suggest that, generally, CRP, possibly with different cut-offs, should be used as a marker of risk and as a guide to manage patients hospitalized for acute coronary syndromes and in outpatients with ischemic heart disease [209].

CRP was noted as a predictor of cardiac rupture after acute MI (>200 mg/L). High hsCRP level measured at first acute MI can predict myocardial dysfunction and heart failure, and it is possible that it plays an important role in the development of heart failure post-MI. A study found that in those who died due to congestive heart failure, the highest mean serum CRP level was 226 mg/L, and in those who suffered sudden cardiac death and those who died from a new MI or non-cardiac causes, the respective values were significantly lower [210,211,212]. mCRP titers were substantially higher in deceased cases within 30 days from the onset of MI than in survivors, mCRP has been considered as potential new and specific biomarker for diagnosing acute MI. Also, people with a median mCRP level or higher were more likely to have more and larger carotid atherosclerotic plaques [83]. Moreover, circulating or microparticle-bound mCRP may be a better diagnostic index than pCRP in MI and peripheral artery disease than using homemade assays [26]. hsCRP levels may have value in the recovery and prognosis of restoration of spontaneous circulation via cardiopulmonary resuscitation after acute MI [213]. CRP has been positively correlated with ventricular arrhythmias regardless of acute MI localization and can possibly predict complications/fatal outcomes [214]. It has been shown that CRP can trigger the complement in infarcted human myocardium, and it can activate cell death in ischemic cells. It has also been associated with MI size increase after ischemia or reperfusion, and indeed, it was revealed that CRP apheresis affected MI sizes and left ventricular function [215,216,217,218].

Moreover, CRP was found to be higher among patients with chronic left ventricular dysfunction and was significantly higher for those with an ischemic origin compared to the others (15.39 mg/L vs. 6.83 mg/L). Nevertheless, previous studies have not provided conclusive data on the prognostic value of CRP for post-infarct left ventricular systolic dysfunction or HF [219,220]. Insights from epidemiological and Mendelian randomization studies (Mendelian randomization studies use measured variation in genes with known function to examine the causal effect of a modifiable exposure on disease in observational studies) highlight that elevated plasma CRP levels were positively associated with incident atrial fibrillation (AF), while the causal effects of CRP on AF were not supported. Another study concluded that increased hsCRP was significantly linked to an increased risk of AF, whereas other data note CRP as a predictor for developing postoperative AF [221,222,223,224]. However, an older Mendelian randomization study found that CRP was robustly associated with a high risk of AF but genetically increased CRP levels were not, suggesting that high CRP per se does not increase AF possibility [225]. Also, a study on patients with paroxysmal AF and no coexistent CVD found left atrial volume enlargement and left ventricular myocardium abnormalities, and that these abnormalities were associated with CRP [226]. Moreover, increased CRP is considered as an independent predictor for sudden death and a prognostic indicator for sudden cardiac death post-MI [227,228].

Additionally, hsCRP has been reported as a prognostic marker in cardiac dysfunction and remodeling in Chagas cardiomyopathy and in hypertrophic cardiomyopathy, and increased CRP has been associated with an increased mortality rate in cases with ischemic or non-ischemic cardiomyopathy. Elevated CRP levels have also been reported in Arrhythmogenic Right Ventricular Dysplasia/Cardiomyopathy (ARVD/C), while another study reported higher CRP values for cases with constructive pericarditis than those with restrictive cardiomyopathy [229,230,231,232,233,234]. Takotsubo cardiomyopathy can show higher CRP titers, and hsCRP has shown prognostic value for adverse events in mildly dilated cardiomyopathy cases but not in dilated cardiomyopathy ones; however, generally higher titers have been associated with the same outcomes, and also, higher CRP has been associated with AF in idiopathic dilated cardiomyopathy (4.59 vs. 2.81 mg/L) and has also been correlated with white blood cell count and overweight [235,236,237]. Significantly abnormal CRP levels (>200 mg/L) have been reported to predict complications and mortality in peripartum cardiomyopathy, and can persist for some months, portending a slower response or nonrecovery in those cases [238,239].

Moreover, increased hsCRP titers were highlighted as a possible predictor for the prognosis of infective endocarditis and idiopathic recurrent pericarditis, and importantly, old data indicate that CRP increases in the pericardial fluid related to an “agonal pericarditis”, which may result from an agonal myocardial necrosis [240,241,242,243]. Serum hsCRP titers can rapidly rise in acute idiopathic pericarditis, and maximal levels are associated with major complications. A normal hsCRP is rare even 2 days after the event, whereas Loeffler endocarditis has also shown elevated CRP levels [244,245]. CRP has been noted for its prognostic role in lymphocytic, eosinophilic, autoimmune, and viral myocarditis [246,247].

A meta-analysis on 83 CVD studies concluded that hsCRP could selectively be used as a prognostic marker for the condition [248]. Another meta-analysis found that elevated CRP levels were linked to Angina Pectoris (AP), particularly unstable AP, and were possibly a risk factor of major adverse events, and also, the authors noted that patients with AP syndromes may be prediagnosed by their serum CRP titers [249]. A study on AP concluded that baseline CRP was higher in women than men, but the event rate was similar in both cases, and that CRP was an independent predictor of cardiovascular risk [250]. Other data indicate that in cases with patients who underwent percutaneous coronary intervention (PCI), an increased hsCRP level was associated with an increased risk of ischemic events, and also, CRP level can an independent prognostic indicator for death or nonfatal MI occurrences following coronary angioplasty but is not linked to the need for repeat revascularization [251,252]. Moreover, a large study revealed that in Coronary Artery Disease (CAD) individuals undergoing PCI with hsCRP ≥ 2 mg/L all-cause death, myocardial infarction, ischemic stroke and revascularization were more likely to occur [253]. Summing up the controversial data from various studies, most guidelines propose hsCRP testing for both primary and secondary CVD prevention, as it has a predictive role in valve pathology and the prognosis of coronary stent thrombosis, restenosis or aortic stenosis in CAD cases, and intracranial arterial stenosis (IAS) [254,255,256].

A meta-analysis concluded that hsCRP may potentially be used as a diagnostic biomarker for abdominal aortic aneurysm (AAA) cases with medium/small aortic diameter but not for AAA patients with enlarged aortic diameter; however, a Mendelian randomization analysis concluded that such association is not causal [257,258]. hsCRP can also be a negative predictor for aneurysm sac shrinkage after endovascular aneurysm repair, and it can also predict long-term outcomes in acute aortic dissection patients [259,260]. In addition, CRP can be significantly elevated (as high as >50 mg/L) even 2 weeks after acute aortic intramural hematoma and was proposed as a useful biomarker, providing incremental prognostic information for possible ulcer-like projection [261].

Data from various studies have concluded that there is a possible correlation between hsCRP and valvular heart disease cases, while high hsCRP levels in paroxysmal and permanent AF cases with Rheumatic Mitral Stenosis (RMS) could favor the hypothesis that low-grade chronic inflammation could be attributable toto AF rather than a consequence [262,263]. hsCRP can possibly predict the progression of chronic RMS; it can be significantly elevated in subjects with mitral annulus calcification, and hsCRP titers can help identify cases with even asymptomatic, moderate, or severe mitral regurgitation, and for this reason, CRP has been proposed as a novel biomarker in patients with calcific aortic valve disease [264,265,266,267]. Moreover, CRP can be notably increased in cases with both left atrial appendage clipping and stapling [268]. Finally, CRP can be a predictor of complications and death in patients with continuous-flow left ventricular assist devices. Increased hsCRP can be seen in patients with off-pump coronary artery bypass grafting and in cases after congenital heart surgery with cardiopulmonary bypass, whereas evidence on CRO for the long-term prognosis of transcatheter aortic valve implantation is still controversial [269,270,271,272,273].

#### 4.1.2. Vascular Disorders

mCRP on endothelial microparticles can also be an unmeasured indicator of peripheral artery disease (PAD), and circulating or microparticle-bound mCRP has been demonstrated as a better diagnostic index than the pentamer in MI and PAD than using homemade assays [26,274]. A study concluded that increased hsCRP titers were notably related to cardiovascular-related and malignancy-related deaths in cases with intermittent claudication [275]. CRP also has prognostic value in (subclinical) atherosclerosis, but several studies have detected this condition in parallel with several other diseases [77,94,197]. Although the literature data regarding the utility of CRP as a biomarker in predicting Venous Thromboembolism (VTE) seem modest and conflicting, its clinical use seems improved in predicting VTE recurrence, cancer-associated thrombosis, and some other pathologies, as well as CVD and mortality in hemodialysis patients and the development of Vascular Access Thrombosis (VAT) in chronic hemodialysis individuals [276,277,278]. CRP also enhances thrombocytopenia and has been reported in other thrombocytosis-related diseases, and also, it possibly modulates the intrinsic risk of cardiovascular events in cases of myeloproliferative disorders [279,280,281].

Elevated CRP is also a feature of some angioedema cases—even in asymptomatic conditions—as well as a potential a diagnostic tool for the differential diagnosis of hypereosinophilic syndrome. Also, it can be elevated in asymptomatic afebrile neutropenia children that are at a high risk for a febrile condition [282,283,284,285]. Furthermore, hsCRP has been a hypertension marker in various studies, and interestingly, a study revealed that plasma CRP titers were substantially related to the CRP gene’s common genetic variants and could predict hypertension; however, the relationship between genotype and CRP levels was independent of any alteration in hypertension risk [77,197,286,287,288]. Generally, CRP genetic variations have been linked to heart diseases, and particularly, heart rate variability seems to be affected even in healthy male twins, possibly due to genetic issues [54,289]. Moreover, CRP has been noted as a marker of severity in Sickle Cell Disease (SCD), while another study showed that in asymptomatic steady-state HbSS individuals, elevated CRP could be protective in SCD, leading to better disease outcomes [290,291].

Finally, increased CRP titers have been reported in some lymphadenitis and lymphagioma types, but no evidence indicates that it is a direct biomarker of such conditions, or generally in lymphatic-related issues; thus, more studies are needed for the potential role of CRP in this field [292,293,294,295,296].

#### 4.1.3. Respiratory Disorders

The literature data reveal that Acute Lung Injury (ALI) survivors have higher serum CRP titers (as high as >100 mg/L in pulmonary edema (ALI) than deceased individuals, suggesting a possible defensive role of CRP that might help in patient improvement; yet, other data show a potential favorable outcome in adult patients with ALI/Acute Respiratory Distress Syndrome (ARDS) compared to young people, with other sources highlighting CRP as a predictor of higher mortality in elderly ALI patients and other teams demonstrating that increased CRP titers were linked to better outcomes in ARDS patients, altogether concluding that the current literature on CRP and ALI/ARDS is somewhat controversial [297]. With the exception of its causal genetic association to chronic airway obstruction, even if not measured at a routine clinical non-research basis, mCRP has been found to be slightly increased in Chronic Obstructive Pulmonary Disease (COPD), and particularly, higher mean CRP titers are related to a larger FEV1 decline, but both previous conditions are affected by smoking, which also leads to high CRP levels [83,297]. CRP can play a role in the pathogenesis of pulmonary-hypertension-associated COPD, and it was suggested as a systemic marker of the inflammatory process that occurs in COPD cases [77]. Additionally, increases in serum hsCRP titers can be related to airflow obstruction and airway inflammation; therefore, it can serve as a possible surrogate marker of airway inflammation in asthma [298,299]. A study concluded that CRP value was also associated with a greater risk of future severe exacerbations but not with mild/moderate exacerbations in steady-state bronchiectasis patients, while another study revealed a positive correlation between hsCRP and stable non-cystic fibrosis bronchiectasis [300,301]. Additionally, CRP titers have not been associated with pulmonary exacerbation severity, but have with specific clinical characteristics in cystic fibrosis (CF) patients. However, another study showed that stable CF cases with increased baseline hsCRP demonstrated worse clinical disease activity and quality-of-life scores at a given level of disease severity [302,303]. CRP can be notably elevated in interstitial lung diseases, and baseline titers are critical for progression to further conditions [304,305]. A recent meta-analysis review revealed that CRP/hs-CRP titers are independently linked to Obstructive Sleep Apnea (OSA), yet it is unknown whether there is a link between OSA severity and increased CRP/hs-CRP levels. Additionally, Mendelian randomization analysis showed a potential causal link between OSA and increased CRP; this recent investigation further cements CRP as a potential OSA diagnostic marker [306].

CRP can be elevated in some other chronic conditions, such as emphysema and chronic bronchitis, showing a persistent inflammatory response, while another study showed that CRP levels were not significantly higher in mild/moderate emphysema cases compared to those without emphysema, yet CRP titers were modestly correlated with FEV(1)% in people with airflow obstruction [307,308]. A study concluded that elevated CRP levels are highly and independently linked to respiratory impairment and more frequent Bronchial Hyperresponsiveness (BHR), and these results indicate that both respiratory impairment and BHR are related to a systemic inflammatory process [309]. Old data also reveal that CRP levels in eosinophilic pneumonia were lower compared to those in bacterial pneumonia, suggesting that the pathogenesis of eosinophilic pneumonia possibly involves the defective secretion of certain cytokines associated with production of acute-phase reactant proteins, like IL-6 [310]. Increased BAL CRP along with procalcitonin have been reported as predictors of ventilator-associated pneumonia [311]. Certain CRP genetic variants may be associated with pneumonia risk, yet these haplotypes are variably linked to baseline CRP levels, and in the case that CRP is a relevant component of innate immunity, the inducibility or tissue-specificity of expression may be at least as crucial as chronic circulating levels [312].

With the exception of being markedly elevated in diffuse alveolar hemorrhage, CRP was proposed as a possible new predictor for adverse outcomes in Pulmonary Arterial Hypertension (PAH) [313]. Moreover, comparing the serum hsCRP titers before and after thrombolysis in Pulmonary Embolism (PE) cases of different severities, it was revealed that hsCRP levels were linked to PE severity, especially those with large embolism areas; therefore, it was proposed as a monitoring biomarker for PE risk stratification, prognosis, and evaluation [314]. A study showed that increased immunoglobulin titers in Pulmonary Alveolar Proteinosis (PAP) patients’ lavage effluent along with abnormal serum immunoglobulin levels and serum CRP note an immune system response to the disease process and proposed that an atypical hypersensitivity reaction possibly is involved [315].

A study suggested that pleural CRP titers provide useful evidence to assess pleural exudates, with the authors highlighting that a level below 20 mg/L suggests a potential malignant origin, while a level above 45 mg/L certainly rules out this possibility [316]. Moreover, with the exception of CRP in pneumothorax and empyema, in which significant increases can be seen, CRP, pleural fluid CRP, and gradient CRP have been proposed as markers for the discrimination of Uncomplicated Parapneumonic Effusion (UCPPE) from Complicated Parapneumonic Effusion (CPPE) [317]. Finally, except for acute bronchiolitis, serum CRP has also been elevated in Bronchiolitis Obliterans Syndrome (BOS) three years post lung transplantation, but only Bronchoalveolar Lavage (BAL) CRP was an independent predictor of graft failure, suggesting that baseline BAL or plasma CRP level may be predictive of post lung transplantation long-term outcomes, and also, even if the precise role of CRP in BOS were not detected, it was demonstrated that local CRP regulated inflammatory components and allograft rejection [318,319].

#### 4.1.4. Gastrointestinal Disorders

A significant elevation in CRP serum titers over the following days post-endoscopic Zenker’s diverticulotomy may indicate esophageal leakage [320]. Furthermore, a study found that CRP was higher in diabetic versus idiopathic gastroparesis [321]. A relatively large cross-sectional study demonstrated that hsCRP could be a marker for inflammation in Irritable Bowel Syndrome (IBS), another study supported values < 10 mg/L to occur in remissions, and a meta-analysis concluded that CRP seems to be a useful biomarker for endoscopic activity in Irritable Bowel Disease (IBD) patients, but another a meta-analysis concluded that CRP essentially excludes IBD in patients with IBS-like symptoms; therewithal, increased CRP was proposed as a marker for short-term mortality in patients with percutaneous endoscopic gastrostomy [322,323,324].

Another study showed that serum CRP was notably higher in food protein-induced enterocolitis syndrome (FPIES) than in food protein-induced proctocolitis (FPIP), suggesting its utility as a marker for differentiating the pathogeneses of these conditions [325]. A rare case report showed that CRP can also be elevated in cases with constipation issues [326]. Importantly, CRP is known as a potential biomarker for acute appendicitis and its severity in children, yet a study revealed that HLX and CTSB genes are possible etiologies for appendicitis and propose a shared genetic mechanism between appendicitis and CRP levels [327,328]. Moreover, the determination of plasma CRP levels can help in diagnosing intestinal obstruction and possibly indicate its benign or malign origin in emergency services [329]. Moreover, CRP values seem to be of little help in differentiating non-specific abdominal pain and surgical conditions that require operative or non-operative interventions, and finally, a systematic review and meta-analysis noted that colorectal anastomotic leakage is associated with higher CRP titers on each postoperative day after surgery, whereas another study highlighted CRP as a prognostic marker for rebleeding in cases with nonvariceal upper gastrointestinal bleeding [330,331,332].

#### 4.1.5. Hepatobiliary Disorders

It has been proposed that inflammatory processes occurring in the liver contribute to the systemic inflammation that characterizes the metabolic syndrome. A study showed that increased liver enzymes secondary to hepatic steatosis are frequent in metabolic syndrome cases, and there exists a direct link between elevated liver enzymes and CRP levels [333]. Subjects with Acute-on-Chronic Liver Failure (ACLF) and Non-Alcoholic Fatty Liver Disease (NAFLD) have also elevated serum CRP concentrations, which has also been reported as an independent risk factor for NAFLD. CRP has been noted as a useful marker in distinguishing steatohepatitis from steatosis in NAFLD cases, and also, it was proposed as a promising prognostic biomarker for non-alcoholic steatohepatitis and the severity of its fibrosis [334,335,336,337,338,339]. Some early dampened CRP responses post-liver resection can reflect a poor hepatic reserve that could have prognostic value [340]. Importantly, a study demonstrated that increased hsCRP titers were linked to an increased risk of Metabolic-dysfunction Associated Fatty Liver Disease (MAFLD) in obese individuals, and it was also positively associated with the severity of liver steatosis and fibrosis; therefore, it may be used as a possible biomarker to monitor and estimate disease severity, while another study concluded that hsCRP can be an independent predictor of poor prognosis—even fatal outcomes—in MAFLD patients [341,342]. Interestingly, a study on rats showed that increased CRP levels can be an early sign of Alcoholic Fatty Liver (AFL), whereas another study concluded that CRP seems to be an accurate marker of alcoholic hepatitis [343,344]. Furthermore, other studies have demonstrated that hsCRP was significantly elevated in liver cirrhosis patients, and that it can predict relevant outcomes, both in cases with compensated and decompensated cirrhosis, for which there have been noted prognostic models incorporating variations in CRP that can predict 3-month mortality. Finally, it seems that CRP may allow for the prediction of early mortality in cirrhosis patients following esophageal variceal bleeding [345,346,347,348]. In addition, obstructive jaundice can be accompanied by elevated CRP levels, and it can also possess crucial clinical values in the differential diagnosis of neonatal jaundice [349,350]. Old data reveal that CRP has been notably increased in subjects with hepatic glycogen storage disease type 1 and in cases with secondary amyloidosis [351,352].

Interestingly, a study showed that increased hsCRP is an independent risk factor for new-onset gallbladder disease [353]. Moreover, CRP has been noted for its high predictive value in predicting acute gangrenous cholecystitis, and it may help in selecting cases requiring emergency cholecystectomy [354,355,356]. Despite there being few data on CRP levels being elevated in cholelithiasis, they may aid in identifying cystic structures during emergency laparoscopic cholecystectomies [357]. Interestingly, various CRP levels have been reported in gallstone ileus (8–347 mg/L) [358]. Moreover, some studies on Gillbert’s syndrome have reported significantly decreased CRP levels, while another study concluded that body mass index and IL-6 predicted 26% of the variance in CRP concentrations [359,360,361].

#### 4.1.6. Pancreatic Disorders

Apart from metabolic syndrome and hypertension, CRP has been associated with Diabetes Mellitus (DM), gestational DM, prediabetes, and diabetes and associated complications, whereas in the Women’s Health Study, a nationwide cohort of 27,628 women without DM, CVD, or cancer at baseline, 188 women developed DM over a 4-year follow-up window [362,363,364]. It was discussed that mean log hsCRP elevated as the number of components of metabolic syndrome increased, and also that CRP adds clinically important prognostic information to this condition [77]. Furthermore, CRP (>150 mg/L in the first hours) has been significantly positively correlated with Severe Acute Pancreatitis (SAP), organ failure, and pancreatic necrosis, but there was less association with mortality. However, another study concluded that the neutrophil-to-lymphocyte ratio along with serum CRP levels at 48 h could be suitable for predicting SAR, and that CRP was a good predictive marker for mortality [365,366]. CRP levels can be higher in patients with chronic pancreatitis and type 2 DM than those with isolated chronic pancreatitis, and also, an inverse correlation has been reported for CRP and subjects with alcoholic chronic pancreatitis [367,368]. Moreover, a study showed that CRP can reliably predict the risks of pancreatic fistula, pancreas-specific complications, and hospital readmission, and should be considered for such conditions post-pancreaticoduodenectomy [369].

#### 4.1.7. Renal Disorders

The emerging literature data reveal that serum CRP levels act as a risk factor for acute kidney injury (AKI), in which it can be increased, and it seems to be positively correlated with AKI severity. hsCRP serum titers have been shown to be related to the development of contrast-induced AKI in ASC cases undergoing percutaneous coronary intervention, and also, it is suggested that elevated hsCRP (>9 mg/L) at admission is an independent predictor for AKI [370]. CRP can exacerbate renal ischemia-reperfusion injury. High serum CRP titers (>3 mg/L) have been independently associated with high Chronic Kidney Disease (CKD) prevalence and debated renal function in predialytic chronic renal failure, with slight elevations per year seeming to likely predict clinical outcomes and possibly the incident hospitalization risk among stage 3–4 CKD patients. And, on the contrary, it was revealed that very low hsCRP levels were associated with higher risks of extended major CVD and mortality, yet hsCRP was unrelated to adverse kidney outcomes, and its predictive performance was not strong [371,372,373,374]. Moreover, a study involving a non-diabetic population showed that CRP seems to be a possible risk marker for renal function loss [375].

CRP can be deposited in kidney lesions in glomerular diseases, whereas significant CRP loss via urine is not as reported in children Nephrotic Syndrome (NS); therefore, serum CRP could possibly be a reliable marker of NS inflammation. And, it was also found that children with steroid-sensitive NS in relapse have increased serum hsCRP titers [376,377,378]. A meta-analysis showed that elevated CRP can influence mortality and cardiac deaths in patients undergoing hemodialysis, with another study highlighting this biomarker to be useful both for guidance in clinical practice and for risk assessment. Additionally, other data demonstrate an elevated CRP in postdiarrheal hemolytic and uremic syndrome with severe multiorgan involvement (>22 mg/L at admission) [379,380,381]. Another study noted low CRP as an independent predictor of arteriovenous fistula patency loss in hemodialysis subjects [382]. Except for hemodialysis, the literature reveals that the majority of elevated CRP concentrations in peritoneal dialysis patients occur with no obvious etiology [383]. Subjects with Goodpasture’s syndrome can have notably elevated CRP concentrations (mean 145.7 mg/L), whereas increased CRP levels (mean 60 mg/L) were evident in ¾ of cases with acute renal infarction [384,385]. Post-renal stent implantation CRP elevation in individuals with atherosclerotic renal stenosis has also been reported; periodic CRP monitoring (2 times/week) can help in the early diagnosis of graft rejection after renal transplantation, whereas mCRP was identified to be secreted in urine after acute rejection episodes [386,387,388]. Significantly elevated CRP titers (mean 47.6 mg/L in all cases, mean 139.6 mg/L in those treated with diversion) have been mentioned in cases with renal colic and may aid in deciding urgent urinary diversion [389].

A study concluded that there exists a notable relationship between serum CRP and self-reported kidney stones in youth, and another study found that significantly increased CRP is a predictive factor for spontaneous stone non-passage in cases with 4–8 mm distal ureteral stones [390,391]. Other data show that serum and urine CRP levels were found to be 20.2 mg/L and 12.8 ng/dL for obstructive stones, and 9.1 mg/L and 9.8 ng/dL for non-obstructive stones, respectively, suggesting that both are potential biomarkers in ureteral stone disease [392]. Furthermore, a relatively normal CRP elevation has also been reported in overactive bladder cases and in those with interstitial cystitis/bladder pain syndrome [393]. In addition, CRP was found to be predictive for the development of systemic inflammatory response syndrome following percutaneous nephrolithotomy [394].

#### 4.1.8. Gynecological and Andrological Disorders

Relatively old data reveal that serum CRP titers can be more than 3 mg/L in women with endometriosis and uterine fibroids, and other data indicate that it is a marker of clinical importance in determining possible reproductive failure, whereas a more recent study showed that following bilateral Uterine Artery Embolization (UAE) as a treatment for leiomyomas and adenomyosis, CRP concentrations can be increased (a median of approx. 80 mg/L) [395,396,397]. In addition, other data show CRP levels above the cutoff level of 0.71 mg/L were independently linked to former Cervical Artery Dissection (CAD) regardless of conventional risk factors [398].

CRP levels can be slightly elevated (median 0.7 mg/L, *p* = 0.026) in girls with self-reported oligomenorrhea and/or hirsutism, which are symptoms of Polycystic Ovary Syndrome (PCOS), some women can have even serum CRP titers >5 mg/L and be at a risk for CVDs, but other data indicate that PCOS by itself does not seem to be linked to increased hsCRP levels [399,400,401]. A study showed a slight CRP increase was observed in women with dysmenorrhea (mean 0.7 mg/L), but another study demonstrated some differences for those with/without pain who have the condition [402,403]. Furthermore, an interesting study noted a higher ultrasensitive CRP (mean 2.5 mg/L) in the entire group and in anovulatory cycles during the luteal phase in healthy adolescents, concluding that this marker may play a role in metabolic risks related to chronic anovulation [404]. On the contrary, other data indicate that CRP values were notably higher in the early follicular phase compared to luteal phase (approx. 2 mg/L vs. 0.7 mg/L), and another study found higher CRP titers in the follicular phase that seem to decline in the luteal phase; yet, other data indicate higher CRP during menses, which becomes lower in the follicular phase and even lower in the luteal phase—a fact that sounds more logical since menses has several features of the inflammatory process [405,406]. As a result, CRP’s variability during the menstrual cycle is evident. Additionally, it was found that high serum CRP levels (even >10 mg/L) are possibly associated with a longer cycle and longer follicular phase length [407]. Other data indicate that a ten-fold progesterone increase was associated with a 23% CRP increase, a ten-fold increase in estrogen was associated with a 29% CRP decrease, and menses was associated with a 17% CRP increase, yet no linkage between ovulation or FSH and CRP was found [408]. Having an hsCRP >3 mg/L has been significantly positively linked to premenstrual mood symptoms [409]. Moreover, CRP has been reported to be a marker of severity in ovarian hyperstimulation syndrome [410].

Regarding pregnancy, it is evident that a slight CRP elevation (median approx. 3.7 mg/L) can be a marker at 4 weeks of gestation, and it was also reported that values of more than about 1.9 mg/L can possibly predict maternal adverse outcomes during pregnancy while considering maternal age, hypertension, and GDM, whereas CRP has been associated in pregnancy complications in PCOS women [411,412,413]. Slightly elevated CRP levels can possibly indicate a sudden pregnancy loss, and importantly, a case–control study showed that CRP variants that influenced circulating CRP concentrations in chronic inflammatory conditions are also related to recurrent pregnancy loss [414,415]. Interestingly, the significantly elevated CRP levels found for women with placental abruption and the lack of CRP difference between bleeding and non-bleeding cases reveal a chronic process underlying placental abruption (PA), yet this laboratory parameter does not seem clinically important for distinguishing between women with and without PA at this point in time [416]. CRP can possibly act as a marker in fetal inflammatory response syndrome and be elevated (median 9 mg/L) in the Microbial Invasion of the Amniotic Cavity (MIAC) and histological chorioamnionitis in women with Preterm Prelabor Rupture of the Membranes (PPROM), but it was not that likely to be a marker of such conditions—and this was also concluded in other studies—whereas it can possibly predict early delivery [417,418,419,420,421]. Nevertheless, an elevated vaginal fluid CRP concentration (>10 mg/L) is possibly a risk factor for intra-amniotic inflammation or/and infection and impending preterm delivery in preterm PPROM [422]. Moreover, high serum CRP titers (>16 mg/L) in the first semester have been associated with preterm birth, whilst elevated amniotic fluid CRP levels (median 110 mg/L, range almost same with controls) can also be a marker of preterm birth [423,424]. Slightly high CRP titers at pre-/post-indicated cervical cerclage can possibly predict very preterm birth, and elevated CRP can be used as a possible indicator of premature delivery when it is linked to premature uterine contractions [425,426].

A study concluded that a high risk of increased hsCRP postpartum may help in reducing postpartum cardiovascular morbidity in women with metabolic syndrome, especially after pre-eclampsia or gestational hypertension [427]. Another study showed that hsCRP was elevated (median approx. 3 mg/L) in women with transient gestational hypertension, pre-eclampsia, or chronic hypertension with minor values’ differences amongst each group, therefore suggesting differences in the prevalence of metabolic syndrome after puerperium, whereas other data indicate that women with higher postpartum hsCRP (>3 mg/L) at 6–12 months post labor may possible have an increased CVD risk after hypersensitive pregnancy disorders [428,429]. CRP can be increased even decades post-eclamptic pregnancy, and even if ultra-high sensitive CRP (mean approx. 18 mg/L) were noted as a possible early marker for pre-eclampsia, other studies concluded that neither baseline CRP titers nor their alterations are associated with pre-eclampsia and recurrent pre-eclampsia, and importantly, a meta-analysis suggested that women with higher CRP levels may have an increased risk of developing pre-eclampsia, yet this association is likely to be affected by other parameters, such as weight [430,431,432,433].

Other studies indicate that slightly increased hsCRP levels can be due to oral contraceptive drugs in women athletes and non-athletes, and old data reveal that CRP can be increased due to intrauterine contraceptive devices [434,435,436,437]. Old data reveal also that CRP can be notably elevated even 1 week post laparoscopic and abdominal hysterectomy, and can be higher in the latter cases, as other data show (mean approx. 28 vs. 44 mg/L), 3 days after surgery, whereas CRP from both methods can have a mean of approximately 10–15 mg/L, as found in another research [438,439,440,441]. As for the healthy-term newborns during the first 48 h of life, CRP values can be slightly increased (approx. 4 mg/L), but another study showed heterogeneity, concluding that CRP values more than 30 mg/L seem to be uncommon until 72 h postpartum [442,443]. Moreover, being pre-pregnancy overweight along with excessive gestational weight gain are risk factors for increased milk CRP, with unknown consequences for the infants receiving varying concentrations; therefore, implications have been speculated for the intergenerational transmission of disease risk [444]. Also, it is yet unknown if the infant could recognize this CRP as an antigen and therefore have a possibility for autoimmunity. Indeed, increased maternal CRP during pregnancy is linked to a higher risk of eczema, and CR in cord blood to a higher possibility of wheezing and lower respiratory tract infections in the first 4 years [445].

Furthermore, a recent novel meta-analysis concluded that CRP is statistically significantly associated with erectile dysfunction and could possibly be a marker or a risk factor for the condition [446]. CRP has also been assessed on penile vascular disease where there can be a slight increase (mean approx. 2 mg/L), it was also reported that epididymitis/orchitis have a greater CRP of more than 24 mg/L, whereas males with acute scrotum can show notably elevated CRP (mean 68 mg/L) [447,448,449]. Finally, a pediatric study showed that CRP > 10 mg/L was a predictive factor for non-salvageable testis in testicular torsion cases [450].

#### 4.1.9. Dermatological Disorders

Relatively normal serum hsCRP levels have been observed in acne vulgaris (mean approx. 2.2 mg/L, values 0–28), whereas another studies highlighted serum and salivary CRP as potential markers of the condition and a marker for post-acne scarring [451,452,453]. CRP levels are elevated in allergic contact dermatitis, Atopic Dermatitis (AD) can also be accompanied by elevated CRP levels (approx. 7 mg/L), with the authors of the study concluding that it has potential use as a marker for disease severity in moderate to severe AD patients, and also, CRP may be used as a novel inflammatory marker in seborrheic dermatitis [454,455,456]. A study found slightly normal CRP levels in rosacea cases (>0.8 mg/L), and a meta-analysis revealed a significant association between rosacea and CRP [457,458]. Another meta-analysis showed an association of hsCRP with eczema and onychomycosis—rosacea was not associated with hsCRP after adjusting for weight and systemic diseases [459].

An interesting case report discusses on an 8-month-old boy with purpuric skin lesions who diagnosed with acute hemorrhagic edema of infancy, for whom a CRP of 30 mg/L was reported [460]. Data on erythema multiforme and epidermal necrolysis are limited, yet CRP can notably be elevated [461,462]. CRP may show divergent values in chronic spontaneous urticaria (median 4.5 mg/L, and approx. 36 mg/L in another study); it can be elevated in 1/3 of cases, and it can also be correlated with treatment efficacy, while other authors have demonstrated that CRP levels were higher in those with a positive autologous serum skin test, concluding that autoimmune urticaria is characterized by low-grade inflammation, while other data have found no association between CRP and the condition [463,464,465,466,467]. Moreover, a study reported a significant association between hsCRP and uremic pruritus in chronic hemodialysis patients [468]. Finally, even if not directly a dermatological issue, ingrown nail can possibly lead at least to local subclinical inflammation, resulting in slightly raised CRP concentrations.

#### 4.1.10. Musculoskeletal Disorders

The current literature includes some meta-analyses on sarcopenia components and its inverse relationship with CRP concentrations; higher CRP titers are significantly inversely associated with muscle mass in community-dwelling populations, lower handgrip and knee extension strength are also related to higher CRP, and amongst sarcopenia components, both hsCRP and CRP are inversely correlated with mass strength [469,470,471]. An interesting study revealed that CRP is increased particularly in creatine kinase responders to muscle damage exercise [472]. Furthermore, a study concluded that CRP seems to be elevated in a minority of myositis subjects—mostly linked to lung diseases and malignancies—whereas another study reported elevated CRP (mean approx. 10 mg/L) in cases with idiopathic inflammatory myopathies [473,474]. Moreover, except for CRP relating to synovitis, in which the data vary, fibromyalgia can be linked to marginally higher hsCRP levels. Its association with elevated CRP (mean approx. 6 mg/L) may be attributed to weight and other comorbidities, and indeed, another study discussed that some patients with this condition show higher serum hsCRP titers, mostly due to being overweight and physical inactive [475,476,477]. Overweight but also underweight individuals with Duchenne muscular dystrophy have been reported for increased CRP levels [478]. In addition, CRP (>3 mg/L) can independently predict greater fatigue that occurred in later months in those over 61ys and can be higher in women reporting fatigue [479]. Moreover, old data consider a CRP concentration of more than 6 mg/L as an additional criterion for the diagnosis of polymyalgia rheumatica [480].

Elderly males with osteoporosis can have higher CRP values (approx. 6.5 mg/L) than those with other conditions, and older females can have higher values than men (even >10 mg/L) [481,482]. A large study showed that Bone Mineral Density (BMD) is inversely and independently associated with CRP concentrations, another study demonstrated a negative association with youth’s BMD and hsCRP, and other data reveal that women with higher CRP were more likely to have more bone loss and had lower possibilities of osteoporotic fractures even after adjusting for weight; but, finally, CRP was not discussed as an indicator of such conditions [483,484,485]. A study found that women with bilateral knee osteoarthritis had higher baseline hsCRP (mean approx. 7 mg/L, and 2 mg/L not counting obesity), and that there was an association, but a recent systematic review concluded that correlation ranges from weak to moderate, and that the evidence is ranges from conflicting to moderate; yet, mCRP has been associated with the condition, and it was proposed as a possible indicator of disease activity [486,487,488]. Not only back pain and related CRP data, but also evidence on CRP and gout again seem conflicting in various studies, with hsCRP >9 mg/L possibly being associated with the pathogenesis of the disease [489]. Nevertheless, hsCRP (median approx. 2 mg/L, 0–44 mg/L) was independently negatively linked to the volume of tibial, but not patella, cartilage in middle-aged women with no evidence of osteoarthritis [490]. Particularly, mCRP has been reported in osteoarthritis, and it was linked to severe symptomatology [83]. Serum hsCRP > 1.0 mg/L was also proposed as an independent marker of Idiopathic Adhesive Capsulitis and as an independent risk factor for the long head of bicep tendons [491,492]. Elevated CRP levels may aid in determining the severity of acute soft-tissue injuries (anterior cruciate ligament tears (median approx. 92 mg/L), Achilles tendon tears (mean approx. 101 mg/L), and acute disc rupture (mean approx. 41 mg/L)) [493]. Finally, old data reveal the role of CRP in orthopedics, with more recent evidence describing its fluctuations in total hip arthroplasty and its serum values being higher in conventional methods, whereas hsCRP can have a predictive role in total knee arthroplasty, for which it was reported that CRP elevation after few days of total knee arthroplasty is gender-specific (men 170 mg/L, women 125 mg/L) [494,495,496,497,498].

#### 4.1.11. Oral, Otorhinolaryngological, and Ophthalmological Disorders

PFAPA syndrome was previously discussed for the presence of anti-mCRP autoantibodies, but CRP can also show significantly elevated values (mean 79 mg/L, mean 185 mg/L) [499,500]. Moreover, low to moderate hsCRP has been associated with gingivitis and chronic periodontitis, with the latter showing slightly higher values [501,502,503]. A meta-analysis provided robust evidence that periodontitis is linked to systemic inflammation as measured by serum CRP levels, and higher hsCRP titers were consistently associated with chronic and aggressive stages of the condition. Other meta-analyses reported that studies often show a CRP of >2.1 mg/L, and a meta-analysis of 10 cross-sectional studies revealed that the CRP weighted mean difference between patients and controls was 1.56 mg/L, and that therapy could show a reduction of about 0.5 mg/L [504,505]. A study concluded that elevated CRP at the 1st and 3rd day of hospitalization (mean approx. 62 mg/L and 20 mg/L, respectively) can be an accurate parameter for the prognosis of peritonsillar abscess [506]. A moderate hsCRP can be found three months’ post-orthodontic treatment and can indicate an enhancement in inflammation during treatment, as well as at least three months after third molar extraction, but another study found that neither erupted nor impacted third molars were associated with hsCRP [507,508,509]. Finally, a recent meta-analysis showed that salivary CRP is a reliable alternative for plasma CRP for the diagnosis and management of medical conditions and oral disorders, including oral lichen planus and periodontitis cases (other medical conditions with be reported in the next section) [510,511,512]. Despite the fact that some studies provide opposing data, hsCRP has been reported to be elevated due to orthodontic-related issues [513]. Importantly, several studies have evaluated salivary CRP for its potential role and possible prognostic value in oral and several other systemic diseases; dietary-related, oral-related, and psychological (stress, depression) data seem contradictory except for periodontitis and stress, malignant issues, and orthodontic appliances, but there is evidence for significant salivary CRP in certain infections, neoplasms and autoimmune conditions in acute and post-acute MI, CVD, CAD, acute respiratory illness, COPD, asthma, pneumonia, tuberculosis, obesity/overweight, pregnancy, physical activity, sepsis, fatigue, sickle cell anemia, subacute thyroiditis, infections, smoking, renal failure, diabetes, metabolic syndrome, acne bulgaris, and, generally, in several other circumstances characterized by systemic inflammation [514,515].

A study on children who underwent a tonsillectomy due to either chronic tonsillitis or adenotonsillar hypertrophy found that hsCRP decreased significantly in the first month, and values are restored a year after surgery [516]. Also, CRP can be higher for central vertigo than for peripheral vertigo (6 mg/L cutoff point) [517]. Also, a persistently elevated CRP beyond the 6th postoperative day was proposed as a potential risk factor for pharyngocutaneous fistula development after laryngectomy [518].

An interesting study showed a correlation for higher CRP levels (per 10 mg/L) and corneal arcus [519]. Other studies revealed a possible association for CRP and ocular ischemic syndrome, and CRP titers can be slightly elevated in branch retinal vein occlusion with no significant association (approx. 3.5 mg/L), but other data indicate that hsCRP > 3 mg/L can have a crucial role in the development of eye vascular disease; yet, another study concluded that despite the relationship between CRP and atherosclerosis, there was no association between elevated CRP and retinal artery occlusion [520,521,522,523]. A relatively recent meta-analysis showed that Age-Related Macular Degeneration (ARMD) is not as associated with CRP at early ages compared to older ages as it can show small-to-moderate increases (>3 mg/L), and also, CRP can show relatively similar values for choroidal thinning during ARMD. It can, however, be slightly higher during ARMD with cataract and show a persistence for even few months after operation [524,525,526,527,528]. Apart from the few contradictory data on myopia and the role of CRP, relatively similar CRP levels have been observed in those with central serous chorioretinopathy, normal tension glaucoma, retinal detachment, and exfoliation syndrome and exfoliative glaucoma, in which there still remains a controversy for the possible role of CRP as being a marker [529,530,531,532,533,534]. Even if considered a genetic disease, hsCRP can be elevated in retinitis pigmentosa, and it would be linked to the faster deterioration of central visual function, thus concluding that it could be associated with disease progression [535]. Finally, elevated CRP levels in tear fluid after continuous contact lens wear have also been reported, but until now, no study has been performed for such condition to provide further evidence for serum CRP levels [536].

#### 4.1.12. Neurological Disorders

The literature data on neurological conditions in parallel with CRP seem to vary, and the genetic parameter (SNPs) is evident in studies for several neurological conditions. hsCRP with a median value of about 17 mg/L could be a possible biomarker in venous infarction in acute/subacute Cerebral Venous Thrombosis (CVT) [537]. Not only early serial, but also elevated post-2-week CRP (approx. > 100 mg/L) may predict worse clinical outcomes in aneurysmal subarachnoid hemorrhage and even secondary cerebral vasospasm [538,539,540]. Despite baseline values (mean approx30 mg/L), an hsCRP of more than 3 mg/L is possibly an independent predictor of poor outcomes 1 year after Intracerebral Hemorrhage (ICH) [541,542]. Interestingly, preliminary evidence on adult rats have revealed that human CRP can increase cerebral infarct size after middle cerebral artery occlusion [543]. Additionally, a study concluded that elevated IL-6 and CRP (>4 mg/L) was an independent predictor for the development of white matter injury in preterm infants with a fetal inflammatory response [544].

A Mendelian randomization study found no correlation between elevated CRP and ischemic stroke prediction, yet other data reveal that increased CRP (approx. > 7 mg/L) in the very early phase can aid in stroke management, that elevated CRP (mean > 20 mg/L) in the very early phase of both ischemic and hemorrhagic stroke were prognostic markers of poor clinical outcomes (with the former possibly showing higher CRP values), and also that moderate hsCRP measured 72 h after ischemic stroke, or transient ischemic attack but not 72 h to 8 days, were linked to an increased risk of 1-year stroke recurrence [545,546,547,548,549]. Moreover, a meta-analysis demonstrated that elevated CRP is associated with the increased possibility of all-cause mortality in acute ischemic stroke subjects. Another study concluded that abnormal CRP may be linked to the stenosis of some cerebral arteries, and also, elevated hsCRP can possibly predict post-stroke disability, apathy, delirium, fatigue, cognitive impairment, and depression [550,551,552,553,554,555,556,557]. However, it was noted that the importance of CRP in ischemic stroke maybe less in older adults than in middle-aged populations, whereas the rapid post-stroke or post-MI hsCRP measurements showing an acute increase are not reflective of pre-stroke levels and may be less reliable for long-term risk stratification [77]. Moreover, elevated hsCRP has been reported to predict further adverse ischemic events and recurrences after transient ischemic attack or even lacunar stroke [558,559,560]. A study reported a moderate cutoff value of 2.9 mg/L for the diagnosis of Trousseau’s syndrome in cerebral embolism cases, and another study reported a median CRP of about 6 mg/L in cases with fusiform intracranial aneurysms [561,562]. Interestingly, cases with higher hsCRP concentrations have been associated with lacunar infarcts even after adjusting for the traditional cardiovascular risk factors, whereas the association between elevated hsCRP and cerebral microbleeds, deep white matter hyperintensity, and periventricular hyperintensity was not significant after this adjustment [563]. Data regarding headache or migraine and CRP seem controversial; despite some studies that result in no association, a possible epidemiological relation between elevated CRP titers (10–20 mg/L) and migraine has been revealed, yet a study found that the integrity of CRP seems not to be a biomarker of episodic/chronic migraine, and also, another large-scale study found that increased hsCRP was associated with headaches lasting ≥7 days/month, which was particularly evident for migraine with aura [564,565,566,567].

Elevated hsCRP has also been reported in older adults with corpus callosum alterations, whereas higher systemic inflammation levels may be linked to lower microstructural integrity in the corpus callosum of non-demented elderlies—thus raising questions on studies for dementia and CRP [568,569]. A study concluded that high hsCRP may serve as an early predictor of the development of a severe paralytic form in tick-born encephalitis. A case with acute encephalopathy as the initial presentation of CADASIL was reported with a CRP of 23 mg/L, while a study on newborns diagnosed with hypoxic-ischemic encephalopathy reported median a CRP of 15.4mg/L [570,571,572]. Furthermore, adults with chronic Spinal Cord Injury (SCI) that are tetraplegic are more likely to show higher hsCRP compared to those who are paraplegic, and the level of lesion and waist circumference have been independently associated with CRP, while higher CRP (mean approx. 9 mg/L) have been seen in the inflammatory phase of heterotopic ossification in soft tissue after SCI; yet, CRP can be elevated even in asymptomatic SCI subjects [573,574].

A recent systematic review on Amyotrophic Lateral Sclerosis (ALS) concluded that CRP seems to be a promising prognostic biomarker, but extensive cohort studies are required to evaluate its prognostic value and accuracy; and a study proved that CRP was as a prognostic biomarker of ALS (elevated CRP > 2 mg/L), yet a Mendelian randomization study concluded that genetically predicted CRP levels may not be an ALS causal risk factor [575,576,577]. Except for the relation of CRP and age-related cognitive impairment, it was previously discussed that mCRP can possibly induce the cellular pathology of Alzheimer’s Disease (AD); nevertheless, data on AD seem controversial since a large study and a Mendelian randomization revealed a causal association between genetically elevated CRP and reduced risk for AD, while another study reported that genetically predicted increased CRP may be a causal risk for AD [578,579,580]. Additionally, individuals with lower CRP appear to have a faster conversion to AD dementia. Another study found that a high baseline hsCRP was linked to the future development of vascular dementia, but not AD, and it was also concluded that in the oldest old, high hsCRP levels are associated with increased odds of all-cause dementia, especially in women [581,582,583]. Furthermore, a meta-analysis found that Parkinson’s Disease (PD) is associated with an increase in CRP, which might be a risk factor for PD, and other data demonstrate that a higher baseline hsCRP may predict early idiopathic PD and poor prognosis—thus, subclinical systemic inflammations may accelerate neurodegeneration in PD—and, indeed, systemic inflammation in Lewy body diseases, particularly in PD, dementia is recorded. Additionally, it was concluded that hsCRP may even be a possible risk factor for PD, and a positive association between serum hsCRP levels and the freezing of gait has also been reported [584,585,586,587,588,589,590,591]. Furthermore, a meta-analysis correlated increased CRP levels with epilepsy, and elevated hsCRP has been recommended as a biomarker in idiopathic epilepsy. This was noted in pediatric epilepsy patients with frequent refractory generalized motor seizures but in low to moderate concentrations [592,593,594].

Studies have concluded that higher hsCRP levels in sciatica patients are closely linked to neuropathic pain [595,596]. Other data show higher CRP as a potential risk factor for Restless Legs Syndrome (RLS), associate the possibility of elevated CRP with severe periodic sleep leg movements in RLS cases, and highlight that moderately elevated hsCRP may be linked to systemic inflammation in RLS [597,598,599]. Interestingly, a case report for acute intermittent porphyria revealed an upper CRP value of 10 mg/L for the condition [600]. Even if data on pregnancy and possible baby autism seem contradictory, the peripheral CRP is elevated in autistic individuals, and although CRP has not been associated with Attention Deficit Hyperactivity Disorder (ADHD), it was revealed that prenatal-pregnancy-related anxiety can predict ADHD in boys [601]. Finally, an interesting case report on primary empty sella syndrome misdiagnosed as recurrent sepsis included a CRP of 152 mg/L [602].

Finally, CRP has been reported in the Cerebrospinal Fluid (CSF) of individuals with different neurological-related and other medical conditions. CSF CRP was found to be correlated with motor and non-motor severity in PD cases and also with mild cognitive impairment in AD individuals, with higher concentrations have been reported for bacterial, compared to viral, meningitis. Generally, CSF CRP can be useful in discriminating between bacterial and viral CNS infections, and it has been connected to aggression in personality-disordered individuals [603,604,605,606,607]. Moreover, various CSF CRP concentrations have been noted in delirium, ALS, severe head injury, aneurysmal subarachnoid hemorrhage, CNS vasculitis and giant cell arteritis, and epilepsy, as well as in neonatal meningitis and non-meningitis systemic inflammatory responses [608,609,610,611,612,613,614].

#### 4.1.13. Mental Disorders

CRP was previously reported for its association with aggressive behaviors and disordered personalities; Mendelian randomization analyses have shown a possible causal protective role of CRP toward schizophrenia and a potential risk-increasing effect on bipolar disorder. Other data show that schizophrenia CRP levels mainly correlate with severity, and during the recrudescent phase, they seem higher when catatonic features, negative symptomatology, and aggressiveness are related; it has not been shown to be correlated to suicidal behavior and ideation [615,616,617]. Other data show that increased hsCRP may be a marker for schizophrenia onset risk and a possible risk factor for common positive symptoms, cognitive impairment, language dysfunction, hypovitaminosis D, microbiota disturbances, and the possibility of cardiovascular and metabolic syndrome in these subjects, as well as greater nicotine dependence in smokers with schizophrenia [618]. Also, maternal inflammation may play a significant role in the offspring’s schizophrenia, and increased maternal CRP has been significantly associated with the condition in their offspring [619]. Despite the fact that some studies have possibly linked CRP in Bipolar Disorder (BD) cases with underlying medical conditions or obesity, a recent study revealed that mean CRP levels were notably elevated in BD adolescents relative to those with anxiety issues and no psychiatric conditions; the mean CRP levels were lower in those with anxiety compared to those with no issues, and although CRP was significantly higher in males and younger individuals, the important between-cohort CRP differences remained after controlling for multiple confounders [620,621,622,623]. It was discussed that CRP concentrations play the role of biomarker to differentiate between major depressive disorder and bipolar disorder depression in both depressed and euthymic states in these individuals [623]. Other data show that BP outpatients with maniac episodes have elevated CRP compared to those without psychiatric disorders, and generally, hsCRP has been proposed as a potentially novel biomarker and endophenotype in individuals with mania [624,625,626]. Moreover, severe delusional symptoms or hallucinations/illusions have also been associated with elevated hsCRP in PD and schizophrenia cases [627,628].

A study revealed that significant inverse associations were found between CRP titers and delayed memory and attention, and also, data on psychosis and elevated CRP are limited [629,630]. Interestingly, a study showed that CRP may be a trait marker for suicidal vulnerability by relating CRP concentrations and a lifetime history of suicide attempts in depressed inpatients, and recently, a meta-analysis concluded that CRP was linked to higher suicidality in individuals with mental issues [631,632]. Moreover, a research study on anxiety/stress disorders and CRP > 3 mg/L demonstrated that an association was observed for panic and generalized anxiety disorders with CRP, and it was attenuated regardless of obesity, multimorbidity and depression [633]. Interestingly, there is evidence on the interaction between CRP and gut microbiome for the risks of anxiety and depression, but these data report no association between anxiety and CRP [634,635,636]. A large cohort study found that panic disorder with agoraphobia is linked to increased CRP, although the effect size of this association is relatively small [637]. Furthermore, compared to healthy subjects, mean CRP has also been shown to be significantly elevated in subjects with obsessive compulsive disorder and has shown moderate to strong associations with psychiatric symptomatology [638]. Interestingly, perfectionistic personalities have been associated with elevated levels of CRP, and these findings held after accounting for other parameters [639].

Additionally, a meta-analysis found that Post-traumatic Stress Disorder (PTSD) is underpinned by the presence of a systemic low-grade inflammation that can lead to higher CRP, whereas other authors discuss that although there are consistent data regarding the relation between CRP and the potential risk and symptomatology of PTSD, there is a paucity of evidence on how CRP could contribute to CNS inflammation and further symptomatology in PTSD cases [640,641]. Despite the fact that a study concluded on an independent association between elevated CRP and insomnia in youth, another study found no significant relationship for CRP and insomnia [642,643].

#### 4.1.14. Thyroid and Splenic Disorders

A bidirectional Mendelian randomization analysis showed that genetically elevated CRP was associated with an increase in both TSH and fT4, but there was no evidence that TSH or fT4 could have an impact on CRP; however, another study showed that TSH is not linked to CRP [644,645]. A case report shows that CRP had elevated values (approx. 7 mg/L) in thyrotoxicosis-induced anemia that had occurred in a patient with painless thyroiditis [646]. CRP has been reported as a risk factor for hypothyroidism in cases with subacute thyroiditis, with a cutoff of 97.8 mg/L [647]. An interesting study revealed that mean hsCRP was slightly higher for those with hypothyroidism, followed by euthyroid and hyperthyroidism individuals, whereas it was found to be correlated to TSH in the first cases, and even if there exist studies concluding that there is no significant association between CRP and hypothyroidism, other studies prove the opposite [648,649,650]. However, another study showed that hyperthyroidism cases have higher CRP than those with hypothyroidism or euthyroid, thus giving rise to further literature heterogeneities [651]. People with thyroid nodules can have elevated CRP levels, and a recent study revealed that a CRP increase is linked to thyroid nodules [652].

Finally, regarding the spleen, notably elevated CRP can be seen in spleen volvulus, spleen thrombosis and infarction, as well as spleen abscess (135 mg/L case-reported for abscess; mean value of a study on spleen infarction was approx. half of this value) [653,654,655,656,657,658].

### 4.2. C-Reactive Protein and Autoimmune-Related Conditions

Autoantibodies against CRP were discussed in the previous section; regarding its role in autoimmune diseases, CRP has thoroughly been discussed, with some authors believing that it possibly has a protective role in such conditions [659,660].

The literature data conclude that CRP could be of prognostic value in evaluating Chagas autoimmune myocarditis progression, and cardiac dysfunction and remodeling [661,662]. Despite its diagnostic utility, individuals with giant cell arteritis can show disparate CRP results—and there exist reports with normal values—whereas CRP can be increased in autoimmune thrombocytopenia; systemic vasculitis; leukocytoclastic vasculitis; other IgG4-related vascular diseases; polyarteritis nodosa in which it can predict poor outcomes if complications are present; and Henoch–Schönlein purpura, in which it can act as a possible biomarker for gastrointestinal involvement; and it was also reported as a marker in anti-neutrophil cytoplasmic antibody (ANCA)-associated renal vasculitis with interstitial arteritis [663,664,665,666,667]. CRP has been reported to be elevated in both antineutrophil cytoplasmic antibody-negative and -positive eosinophilic granulomatosis with polyangiitis. Also, hsCRP was found to be higher in Wegener’s granulomatosis cases that are in relapse, and serial CRP measurement fills the urgent need for an objective index of the activity of this disease [668,669,670,671]. Apart from Kawasaki disease, in which data are contrasted, some authors believe that arteriosclerosis is an autoimmune condition; hsCRP has been correlated with disease severity, and a study found that SNPs affect arterial pulse wave velocity in healthy people [672,673]. Studies on paroxysmal nocturnal hemoglobinuria and CRP are limited, and notably elevated CRP values can be seen in some case reports—possibly in relapses [674,675]. Data on acquired hemolytic anemia or hemophilia anemia (acquired factor VIII deficiency) and CRP are limited, and until now, notably elevated CRP levels have been reported during other underlying medical conditions, yet there have been patients reported with significantly increased CRP values possibly in relapses. There also exist scarce data on common variable immunodeficiency and X-linked agammaglobulinemia subjects, yet a study found that those cases have higher CRP values, predisposing them to a high CVD risk [676,677,678,679,680]. CRP, in parallel with autoimmune myelofibrosis, is not as studied [681]. Additionally, type 1 diabetes can be accompanied with increased CRP that can also be linked to underlying obesity, and a study concluded that elevated CRP may provide an extra marker for the risk of progression of this condition [682,683,684].

There is no utility of CRP in differentiating cryptogenic organizing pneumonia from community-acquired pneumonia, yet higher values can be useful in predicting possible relapses [685]. A two-sample Mendelian randomization study indicated that elevated serum CRP levels have been linked to an increased possibility of the development of Idiopathic Pulmonary Fibrosis (IPF) in European ancestry cases, and another study on IPF with various parameters showed that initial CRP diagnosis levels were associated with poor survival; however, larger studies are required to confirm such data since it was suggested that CRP may serve as an endogenous antifibrotic factor acting in lung fibrosis [686,687].

CRP can also be elevated in eosinophilic esophagitis, but further studies are needed to define its levels [688]. Studies on CRP and autoimmune gastritis as well as autoimmune hepatitis are limited, but a study found elevated CRP in elderly onset autoimmune hepatitis, and a case report showed that it can be notably higher in possible relapse state [689,690,691]. Case reports on both type 1 and type 2 autoimmune pancreatitis revealed increased CRP levels in remission and flares, but further observational data are still absent [692,693]. Furthermore, a recent study concluded that elevated CRP is associated with active Behcet’s disease, which, in reality, is not characterized by specific autoantibodies, and another study found that postoperative CRP levels in those cases with intestinal issues undergoing surgical resection were linked to postoperative outcomes [694,695]. A study revealed that CRP can be elevated in Celiac disease yet with no significant differences between complicated and uncomplicated cases [696]. Although CRP is supposed to be a reliable biomarker in Crohn’s disease, a study found that specific SNPs were linked to a restriction of CRP elevations during activity, while another study concluded that CRP is less important as a disease activity marker in cases with ileal, than those with ileocolonic or colonic, disease [697,698]. In addition, it was earlier discussed that autoantibodies against CRP are elevated in lupus nephritis, in which CRP can be elevated, and also it was found to be colocalized with IgG, C1q, C3c, and dsDNA in electron dense deposits in the glomerular basement membrane/subendothelial space of some patients [699]. A study highlighted that CRP is a strong reflection of colon-wide mucosal inflammation and allows for the reliable evaluation of inflammation throughout the colon in active ulcerative colitis [700].

Two observational studies concluded that CRP is elevated among those with alopecia areata [701,702]. VEXAS syndrome (vacuoles, E1 enzyme, X-linked, autoinflammatory, somatic syndrome) is supposed to be a new autoimmune disorder, and authors of a study reported elevated CRP in those with severe manifestations [703]. Apart from autoantibodies against CRP that were previously discussed in both antiphospholipid syndrome and SLE, hsCRP was found to have a predictive role and also to be higher in those with secondary antiphospholipid disease (compared to primary state); whether it evolves into SLE or not, the literature reveals that SLE individuals have elevated CRP, including in remission periods, yet its response during flares seems to be incomplete and not always linked to disease activity [704,705,706]. Moreover, mCRP has been reported in SLE [83]. Data on bullous pemphigoid are limited, but CRP can be notably increased, possibly in relapses, whereas evidence regarding CREST syndrome describes elevated CRP values [707,708]. Although CRP can be elevated in dermatomyositis, as few data demonstrate, it can be less than 1 mg/L during childhood dermatomyositis [709,710]. Old data reveal that increased CRP has the ability to enhance the acute inflammatory process in erythema nodosum, and some case reports reveal significantly elevated values (as high as >200 mg/L) [711,712]. The median CRP level in cases with hidradenitis suppurativa was 115 mg/L (range of 10–867 mg/L) and could be a potential marker of the disease, yet other authors discuss that CRP levels were only notably elevated in high inflammatory activity [713,714]. Data on lichen sclerosus are limited, but CRP can be elevated both in salivary and serum samples from oral lichen planus cases [715,716]. Furthermore, a study concluded that cases with moderate-to-severe plaque-type psoriasis had active systemic inflammation, which was demonstrated by increased CRP, and its values were correlated with skin disease severity, while another study highlighted that CRP may be considered as a useful marker of psoriasis severity, which could also be used to monitor psoriasis and its treatment; psoriatic arthritis can also show elevated hsCRP, as seen in various studies, yet further data about its utility as a biomarker in this condition are limited [717,718,719]. Vitiligo can also be accompanied by slightly higher hsCRP, but the local inflammation it induces has an insignificant effect on CRP levels [720]. Importantly, mCRP was detected in urticaria, eczema, and psoriasis cases, and it was higher than controls [83].

An old meta-analysis discusses that in terms of discriminative capacity for Ankylosing Spondylitis (AS), the available data on CRP are inconclusive, but other more recent studies reported increased hsCRP as a possible marker for the disease activity and development of future spinal immobility. Also, in AS cases, both baseline and post-baseline serum CRP titers may predict response to treatment, but even if various data conclude that CRP SNPs are associated with AS, some of them are independent of disease severity [721,722,723,724,725]. Some case reports reveal that CRP can be notably elevated during the diagnosis of eosinophilic fasciitis [726,727]. Apart from myasthenia Gravis, on which data on CRP are limited, and polymyositis, in which CRP can be elevated but still with no clinical utility in being a single biomarker, CRP can be an indicator of disease activity, and persistent elevated values may predict amyloidosis in juvenile chronic arthritis. However, it has been reported as a predictive biomarker of mortality and macrophage activation syndrome in adult onset Still’s disease—particularly, the monomeric form of the protein has been highlighted as a useful marker in this condition [728,729,730,731]. It seems controversial whether mixed connective tissue disease should be considered as a distinct disease entity—in which CRP values have been reported to be elevated; apart from palindromic rheumatism, in which CRP can be increased, and except for the autoantibodies against CRP in RA cases that were previously discussed, old data revealed that CRP that is not of local origin is bound to the synovium in RA cases, and also, serum values are increased and can be correlated with radiographic changes, whereas recent evidence note CRP as a promising novel inflammatory marker for assessing disease activity in RA individuals. It may also help in the prediction of CVD in those cases, but a prospective study on women concluded that CRP does not have a substantial effect in predicting incident RA [732,733,734,735,736]. Increased CRP values have been recorded for reactive arthritis, and also, old data reveal that CRP is elevated in polymyalgia rheumatica subjects and can respond to treatment, and a recent study found that increased CRP was associated with treatment-based relapses; specifically, mCRP has been reported in RA and polymyalgia rheumatica [737,738]. CRP can be notably elevated in relapsing polychondritis, but there can also exist cases with a minimal increase [739]. Moreover, apart from Raynayd’s syndrome, on which data are somewhat limited, a study found that CRP is elevated in one quarter of scleroderma patients, especially early disease, and is correlated with disease activity and severity, and another study concluded that CRP can predict poor prognosis in individuals with systemic sclerosis [740,741].

Data on Meniere’s disease and CRP are limited; a study found that CRP tends to be elevated in autoimmune inner ear disease patients, but another study reported that more than half of these cases had an elevated CRP, and studies on Cogan’s syndrome and CRP are sparse [742,743,744]. Again, data on Meniere’s disease and CRP are sparse, yet it can be notably elevated (as high as about 50 mg/L) in cases with Sjogren’s syndrome, but old data revealed that few cases with this disease were found to have minimal to moderate increases in CRP levels, and those with increased CRP did not differ clinically from those with normal values, thus concluding that this syndrome is characterized by relatively low systemic inflammation [745,746]. Even though data on uveitis as well as on ocular cicatricial pemphigoid are limited, there is evidence for systemic inflammation and possible CRP increase [747,748].

Studies on anti-N-methyl-D-aspartate receptor encephalitis potential relapses are limited and opposed, data on autoimmune autonomic ganglionopathy and myalgic encephalomyelitis/chronic fatigue syndrome are again limited, and so are data on autoimmune hypophysitis—with a case report showing high CRP possibly in relapse [749,750,751,752,753]. Moreover, CRP can be elevated in Guillain–Barré syndrome, in which it can possibly be a risk factor for disease severity, particularly in adults [754,755]. CRP was earlier discussed for its ability to suppress Th1 cell differentiation and alleviate experimental autoimmune encephalomyelitis, and data on Multiple Sclerosis (MS) show that hsCRP is slightly elevated in those individuals, yet a study found the increase is non-significant compared to controls, except for cases with relapses, in which it is notably higher. Other data concluded that CRP values were not associated with MS risk, and a systematic review toward the topic demonstrated that results regarding CRP were inconsistent, and the current literature does not favor the clinical utility of CRP as a diagnostic or prognostic biomarker in MS cases [756,757,758]. Furthermore, data on neuromyelitis optica are contrasted and limited [759,760]. In addition, there are limited and contrasted data regarding CRP in chronic inflammatory demyelinating polyneuropathy [761,762]. CRP can also be notably higher in atypical presentations of POEMS syndrome, but data are again limited [763].

There are few reports on autoimmune polyglandular syndrome types; however, elevated CRP is not studied further [764]. Moreover, elevated CRP appears to identify a subset of cases with more severe disease, but a study proposed hsCRP as a useful marker to discard sarcoidosis, and another study found that children with pediatric sarcoidosis were associated with elevated CRP values. Other data reveal that hsCRP can be a more sensitive marker for active cardiac sarcoidosis, yet old data demonstrated that active pulmonary sarcoidosis was associated either with no rise or with only a modest rise in serum CRP [765,766,767,768,769]. Moreover, a study reported immune dysfunction and increased CRP in children with Castleman’s disease, but another study did not report a CRP increase for all of the participants [770,771]. Except for Grave’s disease, on which data are few, CRP has been reported to be elevated in autoimmune thyroiditis, but it cannot be used as a sole biomarker, and hsCRP has been noted as being elevated in Hashimoto’s thyroiditis, highlighting a possible chronic systemic inflammation [772,773].

Finally, it should be highlighted that CRP can be elevated in various IgG4-related diseases, yet the current literature has shown that such conditions may coexist with other underlying issues, mainly malignancies; thus, it is somewhat difficult for CRP values to be precisely defined only for IgG4-related diseases in those cases [774].

### 4.3. C-Reactive Protein and Neoplasms

The present literature shows that CRP has been largely studied in cancer, and in a large representative cohort of consecutive solid tumor adults, the risk of death was clinically and statistically significantly greater with a high mCRP, and this was independent of some other studied parameters; when mCRP values > 10 mg/L were subcategorized, a higher mCRP was always worse, and even among cases with normal values, statistically and clinically significant shorter survival was seen at >5 mg/L [775]. A recent prospective cohort and Mendelian randomization analysis concluded that CRP was a potential biomarker to assess overall cancer risks as well as 12 site-specific cancers, while there was no association for genetically predicted CRP and cancer risks [776]. Another recent study found that CRP trajectories play a crucial role in the occurrence of cancers, particularly in the lung, breast, bladder, stomach, colorectal, liver, and gallbladder and extrahepatic bile duct cancer, and leukemia [777]. An interesting review on CRP and cancer has demonstrated that plasma CRP is not selective for any specific cancer type, CRP > 10 mg/L have been correlated with active and advanced cancer conditions or can be markers of complications. Notable CRP increases (above 50–100 mg/L) are associated with advanced stages/metastasis and poor prognosis, but the importance of hsCRP is still unknown with no proven value; however, higher CRP could possibly predict resistance to certain chemotherapies [778].

Increased baseline CRP concentrations have been linked to shorter survival and the development of second cancers in cases with chronic lymphocytic leukemia, and a study found no significant differences in CRP levels between acute lymphoid and myeloid leukemia that can be useful as an indicator for disease course, and that they were was reduced after treatment, while another study in acute myeloid leukemia and myelodysplastic syndromes found that transplant-related mortality was linked to the pre-specified threshold of CRP > 10 mg/L [779,780,781]. Elevated CRP has been reported to have an independent prognostic impact in myelodysplastic syndrome subjects, whereas elevated values can indicate clonal hematopoiesis and non-hematological comorbidities in cases with low-risk myelodysplastic syndromes [782,783]. Moreover, some other data have linked CRP elevation with myeloproliferative disorders, and other data have revealed a possible CRP SNP association with certain myeloproliferative neoplasms (primary myelofibrosis and essential thrombocythemia) [784]. Some old data seem to be contrasted since a study concluded that CRP did not reflect disease status in multiple myeloma patients, but another study highlighted its independent prognostic significance for the condition. More recent data demonstrated that a preoperative CRP increase is predictive for prognosis in myeloma bone disease post-surgery, and also, elevated values identify a high-risk subgroup in multiple myeloma individuals undergoing delayed autologous stem cell transplantation; however, generally, CRP has been reported as a predictive marker of cachexia in myeloma and lymphoma cases [785,786,787,788,789]. CRP can be a possible prognostic marker for survival in diffuse large B-cell lymphoma (DLBCL) and follicular lymphoma, while generally, notable increases have also been reported in some other non-Hodgkin’s and Hodgkin’s lymphomas as well [790,791,792,793,794,795,796]. For instance, elevated CRP was found to be an independent prognostic marker for poor outcomes in peripheral lymphomas, angioimmunoblastic T cell lymphoma, extranodal natural killer/T cell lymphoma, and most anaplastic large-cell lymphoma cases [797,798,799]. Nevertheless, interestingly, an old study on hematologic malignancies concluded that neither malignancy itself nor its treatment considerably affected CRP responses [800].

A study on CRP and risk for lung cancer concluded that there is a possibility for its incidence in those with elevated concentrations; however, a recent multiethnic bidirectional Mendelian randomization did not reveal a causal association between CRP and lung cancer, and neither did another large study that also supported that circulating CRP could aid as a prediagnostic marker of lung cancer as early as 8 years in advance for current smokers [801,802,803]. Nevertheless, a study found that notably high CRP in conjunction with at least one symptom was correlated with a greater than fourfold higher odd of lung cancer [804]. Additionally, elevated CRP is an independent poor prognostic serum marker in small cell lung cancer and also is a potential poor prognostic indicator for non-small cell lung cancer as it has been correlated with tumor size and staging, with values > 40 mg/L possibly predicting metastasis [805,806]. Apart from some reports with elevated CRP in lung squamous cell carcinoma and large cell carcinoma, CRP levels have predicted a lack of response to treatment in advanced lung adenocarcinoma individuals with or without EGFR mutations, thus serving as a prognostic marker and objective indicator for clinical practice [807]. Moreover, baseline CRP has been an independent predictor of 5-year overall survival in cases with malignant pleural mesothelioma with patients who underwent extrapleural pneumonectomy [808].

Apart from throat cancer, in which CRP was reported to be notably elevated, a meta-analysis concluded that high CRP levels were significantly associated with poor overall esophageal cancer survival, another study demonstrated that notably increased preoperative CRP predicts poor survival prognosis in subjects who have undergone curative resection for esophageal squamous cell cancer, and other data indicate that CRP can generally predict the survival rate of that condition; yet, it seems that there is insufficient evidence to support use of CRP alone to predict survival in esophageal and junctional adenocarcinoma patients [809,810,811,812]. Importantly, a SNP CRP has been reported to be a determinant of serum CRP levels after major esophagectomy for thoracic esophageal cancer [813].

There are several data proposing CRP (mean approx. > 80 mg/L) as a marker for diagnosis and prognosis in stomach cancer/gastric carcinoma patients, and that it can independently predict short-term survival for stage IV gastric cancer patients. A meta-analysis revealed that basically, increased pretreatment CRP ≥ 10 mg/L has been significantly associated with worse outcomes either in early or advanced stages; therewithal, it might be a marker for metastatic gastric cancer [814,815,816,817,818]. Generally, in gastrointestinal cancer, increased CRP is associated with progressive disease, advanced stages of metastatic cancer, and poor survival [738]. Some case reports show notably elevated CRP values (>100 mg/L) during the diagnosis of extraintestinal Gastrointestinal Stromal Tumor (GIST), and a study indicated that CRP was higher while taking into account the location of the lower digestive tract, larger tumor size, and the higher mitotic index of the specific GIST [819,820,821].

Except for pseudomyxoma peritonei, in which data revealing notable CRP increases arise mostly from case reports during the diagnosis of the condition, a study concluded that CRP kinetics and concentrations are decisive predictive markers of early and late postoperative complications after cytoreductive surgery with hyperthermic intraperitoneal chemotherapy in patients with peritoneal carcinomatosis, and another study found that elevated CRP concentrations are correlated with poor survival in patients suffering from peritoneal carcinomatosis of colorectal origin. Moreover, increased CRP was proposed as a marker for the differential diagnosis of malignant and benign ascites in both serum and ascetic fluid [822,823]. Although higher hsCRP may be linked to a higher risk for colon, but not rectal, cancer, prediagnostic CRP is associated with a higher risk of colorectal cancer, but a Mendelian randomization analysis found that that circulating CRP is unlikely to be a causal factor in colorectal cancer, and also, other data indicate that notably elevated CRP is associated with colorectal cancer mortality [778,824,825]. Even if a large study concluded that there is no significant association between CRP levels and the incidence of adenomas, advanced neoplasms, or serrated polyps, another more recent study found that preoperative CRP is correlated with the colorectal polyp histological type [826,827].

Regarding liver cancer and except for hepatoblastoma, on which data arise mostly from case reports illustrating notable CRP increases, a dismal prognosis in patients with aggressive hepatocellular carcinoma was linked to elevated CRP at diagnosis (optimal cutoff 10 mg/L), which may lead it to become a useful marker for patient selection and management [19,768,828]. CRP at the time of diagnosis (>12 mg/L) has been reported as a novel indicator for the prognosis of cases with perihilar extrahepatic cholangiocarcinoma, and elevated pre-operative CRP has been associated with poor clinical outcomes in intrahepatic cholangiocarcinoma patients who underwent hepatectomies with regional lymphadenectomies, yet another study concluded that CRP is a promising immunohistochemical marker to differentiate intrahepatic cholangiocarcinoma from other adenocarcinomas, and its expression was correlated with better prognosis [829,830,831]. In addition, increased CRP levels have been reported to be of value in predicting the outcome and prognosis of cancer-associated gallbladder resection [357]. A Mendelian randomization analysis concluded that gallstones and obesity were causally linked to gallbladder cancer [832].

A study concluded that raised serum CRP levels at the time of presentation of advanced pancreatic cancer indicates a poor prognosis regardless of biliary tract obstruction [833]. Other data have shown that CRP can predict outcomes in pancreatic neuroendocrine tumors, that preoperative elevated CRP represents a significant independent prognostic factor that predicts poor outcomes in cases undergoing curative resection for pancreatic ductal adenocarcinoma, and also that postoperative low serum CRP titers a week after resection were prognostic indicators of better survival [834,835].

CRP can predict mortality, treatment outcomes, and tumor recurrence in solid tumor renal cell carcinoma and other digestive tumors, while preoperative concentrations can predict survival after partial nephrectomy [778]. It seems that for renal cell carcinoma, postoperative serum CRP titers and kinetics hold the most predictive values, and regarding bladder cancer, CRP has been linked to disease progression in non-muscle invasive bladder cancer and to the stage of and survival for muscle invasive bladder cancer [836]. Moreover, a study found that CRP concentrations prior to systemic treatment possibly have a prognostic significance and may enable better risk stratification for cases with metastatic urothelial cancer of the bladder, and other data indicate that increased preoperative CRP is linked to worse outcomes in patients undergoing radical cystectomy for transitional cell carcinoma of the bladder [837,838].

A recent meta-analysis found that CRP can be used as a possible prognostic indicator for a variety of gynecologic malignancies, such as cervical cancer, ovarian cancer, endometrial cancer, and vulvar cancer [839]. Yet, a study found that CRP combined with other markers could be beneficial to distinguish leiomyosarcoma from especially degenerated or atypical leiomyoma [840]. Apart from fallopian tube cancer, on which there exist relatively few data, an interesting study showed that a 67% higher ovarian cancer risk was found for women with CRP > 10 mg/L compared those with <1 mg/L, while CRP > 10 mg/L was positively associated with the risk of mucinous and endometrioid carcinoma [841]. Furthermore, regarding breast cancer, increased CRP values are associated with reduced overall disease-free survival and higher mortality, yet hsCRP seems not to be predictive of post-menopausal breast cancer occurrence in apparently healthy women [778]. Some Mendelian randomization analyses have supported the causal association of CRP with prostate cancer, whereas other data show that CRP concentrations are not predictive in clinically localized prostate cancer compared to advanced stages, with high levels correlating with metastases and poor survival [778,842]. Despite the fact that a study found no association between CRP and the risk of testicular or penile cancer, other data indicate that high preoperative serum CRP titers (>15 mg/L) have been associated with poor survival in cases with penile cancer, and also, an analysis revealed that CRP > 20 mg/L is optimal for predicting lymph node metastasis [843,844,845]. Few data reveal elevated CRP values in germ cell tumors, and most case reports discuss the diagnosis of the condition; thus, evidence there is somewhat limited.

Data on non-melanoma skin cancer in parallel with CRP seem to be in relatively short supply; some case reports reveal notable CRP increases in skin squamous cell and basal cell carcinoma patients, and a study has shown moderately elevated CRP values in cases with advanced Merkel cell carcinoma [846]. In melanoma where CRP impairs adaptive immunity, a study demonstrated that compared to CRP < 10 mg/L, CRP ≥ 10 mg/L conferred poorer overall survival in subjects with any stage, stage I/II, or stage III/IV disease, and worse disease-free survival for those with stage I/II disease. Also, another study highlighted CRP combined with the lymphocyte-to-monocyte ratio as a marker for melanoma recurrence in stage III melanoma cases with microscopic sentinel lymph node metastasis [847,848,849]. Moreover, other data indicate that CRP>10 mg/L is possibly linked to resistance to IL-2 therapy, and it is possible that genetic variations underlying overweight and elevated CRP also contribute to worse melanoma patient survival [850,851]. Interestingly, higher CRP values have also been related to better metastasis-free survival in treated cases with uveal melanoma [852].

Even if some case reports reveal elevated CRP values in benign bone cancers, but data seem sparse, and data on Paget’s disease seem contrasted, there exists a study concluding that baseline CRP seems to be an independent predictor for the overall survival in cases with dedifferentiated chondrosarcoma [853]. Furthermore, CRP has been reported as a prognostic factor for children with Ewing’s sarcoma, chordoma, and osteosarcoma, yet a meta-analysis concluded that higher CRP expression indicates a poorer prognosis in cases with bone neoplasms—except for Asian populations [854,855,856,857]. Even if data on soft tissue sarcomas are somewhat sparse, a meta-analysis concluded that elevated pretreatment serum CRP level could serve as an independent risk factor for poor disease-specific survival and disease/recurrence-free survival in those patients [858].

Another meta-analysis showed that an increased pretreatment of CRP indicates poor prognosis in head and neck squamous cell carcinoma, yet other data have not supported an association between preoperative CRP values and the development of recurrence or metastases, and also, CRP increase during concurrent chemoradiotherapy is a poor predictive marker for head and neck cancer [859,860,861]. The role of CRP, as an independent prognostic marker in cases with oral and tongue squamous cell carcinoma, has also been proved [862]. Data on mouth cancer are few, yet prediagnostic concentrations of CRP have been associated with the subsequent development of oral cancer, and higher values were seen in squamous cell carcinoma, while lower values were recorded for leukoplakia, oral submucous fibrosis, and lichen planus. Nevertheless, another study concluded that CRP can be raised in oral submucous fibrosis, but there was no statistical significance compared to oral squamous cell carcinoma, in which values were significant and also showed a positive correlation with primary tumor size [863,864]. Also, evidence suggests that CPR is higher in cases with oral premalignant lesions, while its increase has been associated with advanced stages in oral squamous cell carcinoma [865]. Moreover, pre-treatment elevated CRP predicts a poor prognosis in cases with locoregionally advanced laryngeal carcinoma treated with chemoradiotherapy, yet another study found that the overall survival was independent of serum CRP levels [866,867]. Several data show an association between moderately elevated CRP and worse outcomes in primary, metastatic, and non-metastatic nasopharyngeal cancer, and the current literature reveals a slightly higher cut-off value in locoregionally advanced tumors (8 mg/L). Moreover, another study concluded that baseline CRP ≥ 3.4 mg/L and CRP kinetics can possibly be useful in predicting the prognosis of metastatic patients treated with palliative chemotherapy [868,869,870].

A shortened progression-free survival has also been reported for sporadic vestibular schwannoma patients with notably elevated baseline CRP (approx. > 31 mg/L), and hsCRP has been associated with poor cognitive function in acoustic neuroma [871,872]. Additionally, an interesting study found that cases with craniopharyngiomas and Rathke’s cleft cysts had higher CRP than those with pituitary adenomas, revealing their higher systemic inflammation [873]. A meta-analysis showed that increased CRP levels have been significantly associated with higher glioma risk, and also, CRP may serve a powerful biomarker for a worse prognosis in glioma patients, as well as an independent predictor for the overall survival in subjects with glioblastoma [874,875]. Importantly, a study showed that CRP tended to be lower in meningioma, and also, there was a CRP increase in meningioma, glioma, and brain metastatic tumors, but it was not significant [876].

Various tumors leading to spinal cord compression and spinal cord metastasis can lead to higher CRP values, and a study demonstrated that CRP > 10 mg/L was associated with significantly higher mortality, and also, only CRP increase correlated with postoperative complications rate [877]. Moreover, while previous studies had found decreased levels in hsCRP in acromegaly individuals—caused mainly by pituitary adenomas—a recent study has shown the opposite [878,879,880]. Additionally, hsCRP was found to be elevated in patients with non-functioning pituitary tumors and growth hormone deficiency, and in women with hypopituitarism, thus highlighting its possible role in the condition [881,882]. Moreover, higher CRP (>10 mg/L) has been associated with thymic carcinoma and neuroendocrine tumors, and it is seems more frequent compared to thymoma [883]. Increased hsCRP has been reported as a marker in Papillary Thyroid Carcinoma (PTC), but other data have demonstrated that CRP may not a have significant increase nor importance in chronic PTC. Additionally, a study found that higher preoperative CRP values have a robust prognostic impact on recurrence-free survival in differentiated thyroid carcinoma cases, and higher preoperative CRP levels were linked to age ≥ 55 years and T3 + T4 [884,885,886]. Moreover, hsCRP can be slightly elevated in patients with asymptomatic primary hyperparathyroidism, and the marker can be elevated in symptomatic conditions, while another study found no significant differences between patients and healthy controls [887,888,889]. It should be highlighted that several other studies have reported significantly elevated CRP in secondary metastatic cancers, and also it has been proposed as a marker for further metastases and worse outcomes.

### 4.4. C-Reactive Protein and Infections

CRP was initially identified from the serum of patients infected with pneumococcus [23,24,25]. Old literature sources reveal that in patients suspected of having been infected by a pathogenic agent, CRP levels of up to 100 mg/L are compatible with all bacterial, viral, fungal, and protozoal infections, and also, CRP response can be delayed >12 h, even in subjects with severe acute infections, with peak concentrations mostly reached at 3d post-symptomatology; Moreover, CRP is possibly reliable for the exclusion of bacterial infection, since two values <10 mg/L and 8–12 h apart can be taken to rule out bacterial infection [890]. It has also been proposed that CRP can be taken into account as an indicator of infection, alongside a body temperature >38.2 °C. Indeed, other old data conclude that a viral infection without bacterial involvement is very improbable if CRP > 40 mg/L, and that high CRP values rule out viral infection as a unique etiology of infection [891]. An interesting study found that CRP > 500 mg/L was influenced by patient characteristics, settings, the etiologies of inflammation, comorbidities and microbiology in 62 years old patients, and also that infections, mostly bacterial, accounted for 88% of episodes, while outcomes were fatal in 36% of all cases and in 61% of cases with active malignancies [892].

Endocarditis, myocarditis, and pericarditis resulting from various etiologies were previously discussed [3,240,241,242,243,244,245,246,247]. A CRP of more than 720 mg/L can predict high-risk individuals with a high in-hospital mortality rate due to infective endocarditis [893]. Except for some notably increased serum CRP titers in myocarditis, there exist reports with lower CRP (0.7 mg/L in a SARS-CoV-2 case!), highlighting the importance of reporting specific evidence on mCRP/pCRP [894,895]. Moderate elevations have been reported in the initial presentations of acute pericarditis, and CRP can reach notably higher values (180 mg/dL) [896,897].

A study concluded that moderate CRP elevations (<100 mg/L) are common in both cases with contaminated blood cultures and in those with bacteremia, and if the CRP concentration is > 100 mg/L while eliminating other causes of marked CRP increases, CRP may be a relatively specific indicator of infection; However, CRP increases are neither completely sensitive nor to detect infection in bacteremia patients, yet the CRP level in Staphylococcus aureus bacteremia cases can be affected by certain SNPs [898]. CRP can be elevated in leptospirosis, and a value > 50 mg/L can be a marker for its differentiation from Dengue fever, yet data on this seem sparse [899,900]. HIV infection can cause elevated CRP values, and CRP could potentially act as prognostic marker of Immune Reconstitution Inflammatory Syndrome (IRIS), which may occur after various related infections [901,902]. Apart from some previously reported coronaviruses, COVID-19 is mainly a vascular disease, and CRP was highlighted to predict possible mortality, but it can be increased even in paucisymptomatic cases and asymptomatic carriers, and important increases may predict post-COVID Multisystem Inflammatory Syndrome in Children (MISC) [2,3,903,904].

Old data reveal that the sensitivity and positive predictive value of CRP > 35 mg/L for the diagnosis of pneumonia was 100%, and lower values were reported for bronchitis and bronchiolitis, while more recent data demonstrate that CRP ≥ 20 mg/L were linked to radiographic pneumonia, bacterial infection, and subsequent hospitalization, yet positive predictive values were too low to be of use in clinical practice. Elevated CRP can be an independent diagnostic marker for pneumonia in children with suspected symptomatology, but low concentrations do not rule out the condition; thus, such prompt evaluation of the marker is needed in children with lower respiratory tract infections [905,906,907]. An interesting review article concluded that CRP testing is neither sufficiently sensitive to exclude nor sufficiently specific to rule in an infiltrate on chest radiograph and the bacterial cause of lower respiratory tract infection [908]. CRP > 37.1 mg/L was positively correlated with confirmed bacterial pneumonia and negatively associated with RSV pneumonia, and additionally, values > 11 mg/L have raised suspicion for bacterial co-infection in children with moderate to severe bronchiolitis, in which CRP might be a prognostic marker of disease severity, yet other data indicate that children with acute bronchiolitis can have elevated concentrations regardless of bacterial coinfection [909,910,911,912]. Large-scale epidemiological studies have supported that CRP > 10 mg/L possibly not only suggests systemic processes, but also pyogenic infections, and a meta-analysis found that it can show considerable promise as a tool to facilitate systematic screening for active tuberculosis, even among HIV cases; however, a study concluded that various host and mycobacterial factors are strongly correlated with baseline CRP responses in tuberculosis [913,914]. Moreover, CRP was noted as a predictor for worse outcomes in H1N1 infection and other related avian influenzas, but other data have shown that values > 100 mg/L may predict bacterial superinfection in influenza (*pneumococci* had the highest CRP values, alfa-hemolytic *streptococci* had the lowest CRP values), and generally, such elevated values can possibly predict bacterial infections in cases with influenza-like symptomatology [915,916,917,918]. Raised CRP concentrations have been reported in infants/children with severe pertussis, and old data have demonstrated that not all psittacosis had elevated CRP values, yet further data are limited [919,920]. Furthermore, a study concluded that CRP > 35 mg/L was highly sensitive in predicting mortality in subjects with malaria, and another study demonstrated CRP may be a biomarker for the early detection and management of malaria severity [921,922]. Additionally, CRP concentrations may be used as early predictors of worse outcomes in invasive aspergillosis after antifungal treatment [923,924].

A study concluded that substantial hsCRP increases in chronic gastritis patients could potentially indicate the severity of acute/chronic mucosal inflammation and the presence of Helicobacter Pylori (HP) infection [925]. CRP can be a useful marker for differentiating between food protein-induced enterocolitis syndrome and food protein-induced proctocolitis, whereas both stage II and III necrotizing enterocolitis complications in neonatals could possibly be predicted by persistently increased CRP after suitable medical management [926,927]. Except for campylobacter enteritis, in which CRP can be notably raised, old data suggest that CRP > 12 mg/L could be a useful tool for predicting children bacterial gastroenteritis, but a more recent study found that CRP > 95 mg/L during the first 48 h is suggestive of bacterial gastroenteritis [928,929]. Old data have revealed CRP’s protective role toward fatal *Salmonella enterica serovar typhimurium* infection in transgenic mice, and another study reported CRP ≥ 20 mg/L in most children with Salmonella infection and in about 17% of those with viral infection, concluding that the very good negative predictive of CRP < 20 mg/L may allow clinicians to reliably rule out *Salmonella* as an etiology of gastroenteritis. CRP can show notably elevated values after norovirus infection, yet the literature provides various ranges in serum titers, with the lowest values to be reported in children [930,931]. Amebiasis cases can show elevated CRP values, and also, an interesting study found that CRP was significantly increased in the blood of children infected with *Entamoeba histolytica* and *Giardia lamblia* (>50 mg/L) [932]. Moreover, a raised CRP level may facilitate the assessment of a febrile child in a typhoid-endemic area [933]. Baseline CRP > 173 mg/L may predict major acute complications in acute sigmoid diverticulitis, another study reported CRP > 200 mg/L as a strong indicator of perforation, while other data have supported its non-significance as a marker. CRP > 150 mg/L has been linked to higher rates of complicated diverticulitis, mortality, and the need for intervention, and also, a systematic review highlighted that comorbidities, non-steroid anti-inflammatory therapy, initial presentations, and baseline CRP > 175 mg/L seem to be predictive of a more severe disease process with a higher possibility for complications and resultant prolonged clinical course; nevertheless, other data have shown that low CRP does not exclude complications [934,935]. In addition, CRP > 100 mg/L can be a promising marker of severe bacterial peritonitis and poor outcomes, and it can be markedly elevated in spontaneous bacterial peritonitis correlated with chronic severe hepatitis B [936,937].

Old data have shown that CRP seems to be undetected in hepatitis C, and recent evidence has demonstrated that the pooled mean CRP level was within the normal range in cases with hepatitis C; however, it was higher in hepatitis B cases, suggesting that CRP expression correlates with hepatitis B disease progression, but not in chronic hepatitis C, and there is also a possibility that cytokine-mediated response is more pronounced in chronic hepatitis B compared to chronic hepatitis C [938,939,940]. Yet, it was previously reported that anti-CRP autoantibodies have been reported in hepatitis C cases, a fact that raises further diagnostic questions for its lower levels in that condition. Acute acalculous cholecystitis case reports reveal different CRP concentrations after different various pathogenic agents (about 5 mg/L for Epstein–Barr Virus (EBV), and more than 200 mg/L in measles infection and various other intermediate values due to other viruses) [941,942,943,944,945,946,947,948,949].

It was discussed that increased CRP (>200 mg/L) can be seen in urinary tract infections, and also, elevated values (≥30 mg/L) was seen in the majority of nonpregnant and pregnant women with acute pyelonephritis [950]. CRP can be increased with different disease entities in cases with lower urinary tract symptomatology, and a study highlighted that moderately elevated levels were noted in overactive bladder; wet, chronic prostatitis, benign prostatic hyperplasia; and acute febrile bacterial infection compared to asymptomatic cases [951]. However, such studies are quite small, and no association between CRP and complicated urinary tract infections has been proved. Data on pyelonephritis seem contrasted, but another study concluded that higher CRP is not accurate in localizing the site of urinary tract infections in young women without clinical signs of acute pyelonephritis [952,953,954]. Additionally, data on urethritis seem limited since some case reports reveal elevated CRP concentrations during diagnosis.

In pelvic inflammatory disease, various CRP cutoffs have been reported that may predict poor outcomes, and values > 11.5 mg/L have been proposed as predictors for tubo-ovarian abscess, and additionally, CRP can possibly be a good marker for diagnosing resistant vaginitis, endometritis, and puerperal infection post-cesarean section, whereas data on high CRP due to salpingitis, colpitis, and endocervicitis arise mostly from case reports [955,956,957,958,959,960]. Except for CRP levels in villitis, on which data seem scarce, even if some studies report CRP’s significance in predicting chorioamnionitis, several other studies conclude that it does not seem to be an effective independent predictor of clinical or histologic chorioamnionitis [961,962,963]. Some common CRP cutoffs have been reported for sepsis (50 mg/L), particularly for neonatal sepsis (10 mg/L) [950]. Other studies have demonstrated that notably elevated CRP may be a marker of mastitis and mastoiditis—in which it can be a predictor for mastoid surgery [964,965]. Furthermore, several studies have shown higher serum CRP titers in orchitis and epididymitis, in which it may aid in differentiation from other medical conditions [966]. A study reported that CRP is present mainly in the semen of the infertile than the fertile prostatitis cases, and another study concluded that it is a predictor of failure of the initial management of acute urinary retention in those cases [967,968]. Regarding sexually transmitted diseases, some data indicate that CRP is not suitable as a marker of persistent low-grade inflammation in *Chlamydia trachomatis*-positive women, and in both syphilis and Jarisch–Herxheimer Reaction (JHR), after its treatment, there have been conclusions drawn regarding hsCRP’s association with JHR prediction [969,970]. Moreover, studies on Human Papillomavirus (HPV) have indicated that CRP concentrations may be higher in HPV-positive individuals [971,972].

Non-diabetic osteomyelitis of the foot can lead to increased CRP levels (approx. 35 mg/L), yet it seems to be a poor marker of the condition, and on the contrary, other data highlight CRP (median approx. > 80 mg/L) as a marker for those with a diabetic foot osteomyelitis [973,974,975,976]. Some data provide evidence on CRP and discitis, and it was also proposed as a negative predictor of septic arthritis, in which a study found that with values < 10 mg/L, it is not as likely for one to have the condition, but another study provided contrasted evidence, concluding that CRP < 10 mg/L cannot exclude the diagnosis of septic arthritis [977,978,979,980,981].

A study found that CRP could not predict the recurrence of cellulitis (apart from orbital cellulitis, for which it can be a marker of idiopathic inflammation in parallel with related edema), and another study demonstrated that most cases with erysipelas had CRP > 200 mg/L; yet, even if CRP were to be notably increased in impetigo, folliculitis, and carbuncles, data are quite limited, with some low values also having been recorded [982,983]. In addition, CRP values can show various ranges in varicella–herpes zoster infected cases (and in post-herpetic infection neuralgia) as well as in leprosy patients, for whom old data have concluded that even if CRP were not useful in monitoring cases, it has limited importance in detecting erythema nodosum leprosum. And, another study found that CRP was associated with *Mycobacterium leprae* in skin lesions, but again, other data indicate that there is a wide range for CRP concentrations in leprosy individuals [984,985,986,987]. Lyme disease has shown various CRP concentrations, and a study concluded that that CRP seems highest when the concentration of spirochetes is highest in the skin and/or bloodstream, and it decreases after the organism’s dissemination to extracutaneous sites in subsequent stages of infection [988]. Additionally, it was discussed in old data that CRP estimations may aid in ascertaining active melioidosis since an increase in CRP to > 10 mg/L led to the diagnosis of the reactivation of infection in three afebrile individuals, but a following study concluded that CRP is not a sensitive marker for the presence of melioidosis, and that a normal level cannot be assessed to exclude acute, chronic, or relapsed melioidosis in febrile cases in/from endemic regions [989,990]. Moreover, an old study demonstrated that there CRP concentrations could reach 225 mg/L in acute tularemia, but there were also low CRP values); therefore, the CRP’s behavior in tularemia resembled tuberculosis, and it did not always aid in the differentiation of tularemia from viral diseases [991].

Studies on pharyngitis and CRP seems scarce; however, a study found a higher mean for individuals with streptococcus C than A infection, but another study highlighted that it may help to rule out streptococcal infection in pharyngitis adult cases [992,993]. Some data revealed elevated CRP in some diphtheria- and mumps-infected cases, yet data are sparse, and also, tonsillitis, peritonsillitis, and peritonsillar abscess cases can have notably elevated CRP values (>100 mg/L) [994,995]. CRP can have various values in sinusitis, and increased values can aid in its early diagnosis [996,997]. Moreover, CRP concentrations can vary from relatively normal to notably elevated values in uveitis cases [998]. An old study has shown various serum CRP levels in acute otitis media cases, but high values (>20 mg/L) were observed in those with bacterial infection [999].

An old study demonstrated that bacterial meningitis can show rapidly increasing CRP levels (>20 mg/L), and slight elevations can be seen in viral meningitis. Another old study reported higher values for bacterial meningitis (>100 mg/L), and recent data have also supported such evidence to a great extent, yet cutoff values among studies are divergent [1000,1001,1002,1003]. Furthermore, studies on John Cunningham virus (JCv) seem limited, but the current literature suggests that it may reactivate after immunosuppression, leading to higher ultrasensitive CRP results [1004,1005]. In addition, a study concluded that notably elevated CRP can indicate mortality in tetanus, yet a case report revealed relatively normal values; however, data are sparse. CRP can be notably elevated in cysticercosis (>500 mg/L), but again, there exist few data on this condition [1004,1005,1006].

Some other infections resulting in various pathognomonics are caused by EBV, in which CRP can be raised or have various persistent levels after initial infection, Cytomegalovirus can increase the risk for CVDs, Hantavirus, Poliovirus and the Zika virus can cause slightly elevated to higher CRP values. As for the Ebola virus, cases with relatively normal values have been recorded, yet notably elevated values can possibly predict worse outcomes [1007,1008,1009,1010]. Moreover, the current literature reveals that raised CRP seems to be significantly associated with an unfavorable course in acute brucellosis, whereas extremely increased values can be seen in trichinellosis [1011,1012]. CRP values can vary in ehrlichiosis, legionnaires’ disease, and rabies infection, whereas evidence on fungal coccidioidomycosis seems contrasted since low CRP values have been reported in immunocompetent cases—yet, such low values may have occurred due to the immunocompromised state [1013,1014,1015,1016,1017,1018,1019]. Data on toxoplasmosis, again, vary, as significantly high CRP values have been reported after infection, but a study revealed no significant difference between cases and controls [1020,1021]. Increased CRP values have also been reported in the acute phase of cholera, as seen in old literature data [1022]. Significantly raised CRP values have been linked to poor outcomes in *Crimean–Congo* hemorrhagic fever, whereas increased values have also been observed in patients with suspected rickettsioses, including acute Q fever, scrub typhus, and murine typhus [1023,1024]. Moderate CRP values have been reported in lassa patients, and notably raised levels can reveal fatal outcomes in Rocky mountain spotted fever and Colorado tick fever [1025,1026].

Finally, a meta-analysis concluded that CRP is s appropriate for detecting neonatal septicemia, and a cutoff value of 61 mg/L can be a sensitive sepsis marker that possibly promotes further inflammation via extracellular vehicles, although it is not specific [1027,1028,1029]. Moreover, several studies have added to the current literature, noting that serum CRP concentrations may predict various post-surgery infections [1030,1031].

### 4.5. C-Reactive Protein and Other Factors

Except for wounds, burns, poisoning, and tissue trauma by accident that lead to significant CRP elevations and during the illustration of physiological disorders, autoimmune conditions, neoplasms, and infections, it was noted that several medical interventions can possibly affect CRP, such as heart stents, bypasses, valve replacements, and other cardiovascular interventions; pregnancy, menses and internal contraception devices; catheters/cannulas; contact lenses, and other ocular and ear interventions; transplants and post-transplantation rejection; as well as dental and various other orthopedic implants.

A major factor that can affect CRP levels seems to be diet, and most studies analyzing CRP values in parallel with underlying medical conditions (mainly cardiovascular issues, metabolic syndrome and diabetes) consider body mass index (BMI) as a factor that possibly increases CRP values. Moreover, not only older data, but also ore recent evidence highlight that obesity is a condition characterized by higher hsCRP values, and obsess adults have increased CRP concentrations, whereas other contrasting data indicate that CRP is a potential causal factor for adult-onset obesity through chronic inflammation [1032,1033]. In other words, obesity with elevated CRP poses a risk for cardiovascular events that again show increased CRP values; therefore, it does not seem to be CRP, but rather obesity itself. Nevertheless, no significant variations for the BMI parameter during the analysis of CRP in cases with underlying medical conditions have been revealed, and this could be attributed to the type of food intake and the diet itself, since people can have normal BMI but feed themselves with foods that affect CRP values. Indeed, the current literature reveals that macrophages exposed to raised glucose levels regulate the regulation of cellular CRP expression and protein biosynthesis and secretion [1034]. Importantly, a study revealed that red meat consumption has a negative impact on inflammatory and glycose metabolism biomarkers [1035]. Moreover, it was shown that hsCRP is likely modulated by dietary fatty acid intake, and another study found that baseline CRP may modulate the diet-induced alterations in plasma lipid and lipoprotein levels; however, another study found that a high-fat diet raises CRP during weight loss [1036,1037,1038]. Except for high meat consumption (mostly red meat), which increases CRP, other studies have highlighted that elevated CRP and low to moderate grade inflammation can generally be caused by ultra-processed food consumption, and the so-called Western diet (high in processed food, trans fats, sugar, sodium, and refined grains), as well as the frequent intake of saturated and trans fatty acids, milk, butter, gluten (particularly in Celiac disease, which has a higher prevalence nowadays), eggs, and other arachidonic acid-containing foods since it causes inflammation, as well as fish. Even most studies provide evidence of anti-inflammatory benefits of fish, environmental pollution leads to the accumulation of heavy metals inside them—which can result in toxicity and inflammation after consumption [1039,1040,1041,1042,1043,1044,1045,1046,1047,1048]. In addition, apart from hormonal factors that affect CRP titers, such as leptin, paraneoplastic hormone production, and lower estrogenic activity in premenopausal women, low levels of vitamin A, K, and hormonal D have been associated with higher CRP levels—but the overdose of such vitamins, especially hormonal D, can cause increased levels [1049,1050,1051]. Moreover, not only certain dietary patterns, but also certain the overconsumption of beverages/alcohol by adults and substance abuse can lead to raised CRP concentrations [436,1052,1053,1054].

Not only can vaccines—including pneumococcus vaccine—induce higher CRP concentrations post-vaccination for some time, but elevated values and systemic inflammation have also been recorded after certain medications, including oral contraception therapy and birth control pills (estrogen), oral hormonal menopausal therapy (which can lead to blood clots), psychotropic drugs (including clozapine and risperidone), antiepileptic drugs (including phenytoin and carbamazepine), specific NSAIDS (including lumiracoxib), antibiotics, chemotherapy (which can trigger cancer/metastases), overdose medication, adverse inflammatory events post-medication, as well as multiple treatments’ failures; therefore, toxicity resulting from various therapeutic agents and their possible metabolites that trigger systemic inflammation is undoubted [1055,1056,1057,1058,1059,1060,1061,1062,1063]. Moreover, higher CRP can possibly show drug resistance in cancer cases [780]. Formerly, it was also discussed that an increase in the amount of CRP occurs after intense anaerobic exercise activity, but other data demonstrate that moderate aerobic activity and not flexibility/resistance exercise, which increases CRP, is beneficial and possibly reduces its levels—compared to a sedentary lifestyle, which increases CRP levels [1064,1065]. Moreover, it was discussed that stress can increase CRP titers, and currently, various data show that stress due to possible socioeconomic issues, poverty and crime, personal health and well-being, lifestyle, or even marriage can cause higher values [436,1066]. Excess sleep, partial sleep, insufficient sleep, and sleep deprivation, as well las frequent and infrequent napping, have all been associated with an increase in (hs)CRP concentrations [1067,1068]. Even if most studies have found that sex can modulate systemic inflammation, there also exist contrasting data highlighting that frequent sex can affect inflammatory markers [1069]. Apart from some previously discussed psychological conditions and cognitive decline, lower IQ levels in youth have been linked to low-grade systemic inflammation, whereas an interesting study demonstrated that daily discrimination, but not direct microaggressions based on sexual orientation, were linked to higher levels of CRP among young sexual-minority men, highlighting the immune vulnerability of the LGBT+ community [1070,1071]. Additionally, hypoxia and altitude as well as cold ambient temperature and increased temperatures have been associated with elevated CRP levels, and furthermore, unstable weather due to global climate change can affect hsCRP, too [1072,1073,1074,1075,1076].

Apart from body creams that contain certain chemicals, which can lead to inflammation, other studies have shown that hyaluronic fillers have immunogenicity, and that Botox could rarely cause systemic inflammation, both of which could potentially affect CRP concentrations, and generally, various natural skin care products could pose a risk for systemic inflammation [1077,1078,1079]. Tattooing can also lead to increased CRP and serious adverse events [1080]. Moreover, red hair was linked to higher CRP, but even if the possibility of hair dye causing this were inconclusive, another study on mice found that repeated exposure to hair dye induces regulatory T cells, whereas other data indicate that the removal of pubic hair can be a possible risk factor for unspecific low-grade inflammation [1081,1082,1083,1084]. Moreover, it was previously discussed that surgeries can affect post-surgery baseline CRP, and also, cosmetic liposuction can cause a transient elevation of acute inflammatory markers, including hsCRP [1085]. Except for prolonged mask use, fashion can also result into adverse inflammatory health events, possibly through the frequent wearing of skinny jeans, while some specific clothes characterized by microplastics can lead to low-grade systemic inflammation. such micro- or nanoparticles can be a constituent of environmental toxicity, which can also lead to low-grade inflammation, and except for smoking/passive smokers and smoke inhalation, which was previously discussed to increased CRP levels, air pollution has already been attributed to systemic inflammation and possibly increased CRP concentrations (i.e., exposed to PM2.5, fumes, home fireplaces/indoor pets/toxic metals, and other occupational-related exposures, such as wood dust, cotton, silica, asbestos, etc.) [1086,1087,1088,1089,1090,1091,1092,1093,1094,1095,1096,1097,1098,1099,1100,1101,1102,1103,1104,1105]. Finally, radiation from both the medical and the industrial field as well as through accidentally exposure can result into inflammation, and some data highlight an acute phase response caused by radiotherapy/radiochemotherapy, yet evidence toward CRP behavior in the extensive use of mobile phones, related devices, and other Wi-Fi wireless technologies seem sparse; nevertheless, some data on UV radiation indicate risks for possible systemic inflammation [1106,1107,1108,1109,1110,1111,1112,1113,1114].

Compared to all the formerly illustrated factors that affect systemic inflammation, most studies on diet and food intake highlight that herbs, as well as fruits, vegetables, grains, and nuts, due to their notable concentrations of multifarious antioxidants, can reduce CRP levels and eliminate subclinical inflammation, and also, the supplementation of vitamins, minerals, and other antioxidants has been associated with reduced CRP titers [1115,1116,1117,1118,1119,1120,1121,1122,1123,1124,1125,1126,1127,1128,1129,1130,1131,1132]. Such evidence suggests that a vegan diet is anti-inflammatory, and it seems it is more likely to lead to lowered CRP concentrations, whereas fasting can have similar anti-inflammatory effects since autophagy itself limits excessive inflammatory responses via preventing the activation of inflammasome, thus regulating DAMP, damaged mitochondria and inflammatory mediator clearance [1133,1134,1135]. Except for NSAIDS and other anti-inflammatory drugs that are designed to initially or partially reduce inflammation (including statins and aspirin), lipid-lowering agents, and anti-CRP drugs, one could suppose that vaccine-related induced immunity can possibly lower inflammation due to infections from pathogens, and also, immunocompromised cases may have lower CRP due to their state, thus having a deviation from certain CRP concentrations’ means of some studies [1136,1137]. Compared to the negative effects of extreme physical exercise, moderate the effects of physical aerobic activity effects on regulating CRP levels was previously discussed, and recently, a study concluded that training induces a suppressive effect responsible for low CRP in athletes (particularly in swimmers) [1138]. Not only physical condition, but also mental and psychologic state affect subclinical inflammation, and it was discussed also that bereavement in widowhood decreases CRP titers [1139]. Overall peace of mind (optimism, happiness, positivity, as well as loss of anger, envy, stress, anxiety, depression, etc.) is also attributable to reduced systemic inflammation, and this can be achieved through music (mainly positive feeling music, classical music and other moderate relaxing types, dancing, singing, painting, reading books), and generally, it has been shown that art and psychotherapies (including Pilates, yoga, etc.) help in mental health and chronic systemic inflammation decrease, and, possibly faith/religion [1140,1141,1142,1143,1144]. Finally, it should be noted that inability of producing CRP in certain individuals for various—mostly pathologic but also other—reasons (not only in immunocompromised cases) can lead to reduced CRP values, whereas immunologically over-reactive cases may produce larger amounts of CRP; also, CRP clearance is different in each case (it can be earlier or longer). Undeniably, the immunity of each individual is unique and case-specific, it cannot be predicted, but it is mostly affected by all previously discussed factors of this section.

## 5. Current Evidence on C-Reactive Protein and Potential Molecular Diagnostics

### 5.1. C-Reactive Protein and Principles of Common Diagnostic Assays

The quellung reaction, also called the Neufeld reaction, which is considered to be the gold standard method for pneumococcal capsular serotyping, can show positive results in certain non-immunoglobulin proteins, including CRP; hence, it can be supposed that initially, this was a reaction being used to detect this protein [23,1145,1146]. Nowadays, the FDA has approved multiple molecular diagnostic tests for human CRP, including assays for conventional CRP and hsCRP. Importantly, it was formerly discussed that until now, a commercially produced test kit for the accurate and sensitive measurement of serum mCRP has not been achieved, because no mCRP-specific antibodies are currently commercially available, therefore mainly pCRP is detected in most diagnostic assays [22,26,105]. Most advanced diagnostic testing formats have been established in recent decades and originated with turbidimetry-based assays detecting CRP in mg/L, followed by more sensitive Enzyme-Linked ImmunosSorbent Assays (ELISAs) as well as chemiluminescent, fluorescent, and electrochemical assays with a low detection sensitivity measured in fg/mL; in parallel with remarkable advances in microfluidics, lab-on-a-chip and fully integrated/automated bioanalytical testing platforms, various novel diagnostic assay formats have emerged, such as Lateral Flow Immunoassays (LFIAs), which has a wide dynamic range and notable sensitivity [1147]. In the last few years, nanomaterial-based signal enhancement, multi-labelling, novel biosensor concepts, and smartphone-based point-of-care detection have provided an impetus to the advancement of next-generation diagnostic assays, whereas the current trend also unravels the wider applicability of robust biorecognition molecules far from conventional antibodies, like aptamers, affirmers, etc., to boost bioanalytical performance [1147]. Apart from some highly instrumental methods, including mass spectrometry, some smartphone-based methods, and some other automated assays that provide results usually within few minutes even in very low concentrations of CRP, some other typical molecular diagnostics are widely available in market, and such test kits are routinely employed for CRP detection.

The first immunoturbidimetric CRP test kits were designed in the early 1990s [22]. Turbidimetric ImmunoAssays (TIAs) have been a heretofore routine—both laboratory and point-of-care feasible—simple, and low-cost method in medicine for measuring CRP and other immunobiochemical markers. Immunoturbidimetry can be direct (immunoturbidimetry antibodies form an immune complex by direct attachment to their target antigen, which is CRP) and microparticle-enhanced tests (the immunoturbidimetry antibodies coat microparticles, and finally, they form immunocomplexes with the corresponding antigen, which is CRP), which are particularly useful for detecting CRP presented in low sample concentrations—several TIAs are widely used for hsCRP [1148]. Most TIA kits for detecting CRP have either polyvalent antibodies, allowing for immunoprecipitation or antibodies bound on nano- or microparticles, which are frequently prepared from latex (and are covalently bioconjugated with F(ab′)2 fragments of anti-CRP Ab), but also, other materials can be used [22,1147]. CRP presence enables the agglutination of latex particles, leading to reduced absorbance at 405 nm largely within 30min (earlier in most cases) [22,1147]. Turbidimetry measures the absorbance of light caused by the sample, whereas nephelometry measures the scattered light at a fixed angle. The latter allows for the quantitative estimation of proteins by determined antigen–antibody reactions, in which agglutination affects the intensity of the transmitted light, which is measured photometrically and correlates with the levels of analyte that is present in the specimen [1148]. In previous years, TIA showed a relative sensitivity compared to nephelometry, yet nowadays, these methods have no substantial differences, and basically, TIA is easier than nephelometry as it measures light intensity in its original axis, and less sensitive and cheaper sensors and less powerful light sources are essential, yet TIA devices can frequently totally compete with nephelometric ones and provide comparable results [22]. Moreover, TIAs have worse specifications compared to those of ELISA or CLIA but are entirely applicable for the molecular diagnosis of CRP [22].

Nephelometric Assay (NMA), from the Greek “νεφέλη” (nepheli, meaning cloud or fog), is based on a dilute suspension of microparticles that will scatter light (usually a laser) passed through it rather than simply absorbing it, while the amount of scatter is determined by collecting the light at an angle. This method is slower compared to TIA, sensitive with consumable costs, and requires highly trained personnel [1149,1150]. Obviously, detection at 0° is impossible due to the transmitted beam’s high intensity, but some laser-equipped rapid analyzers with a mask to block the transmitted beam are capable of operating at quite low angles. Both the antibody and the antigen are blended in concentrations such that smaller aggregates are formed with no quick bottom settlement, the amount of light scatter is counted and compared to the amount of scatter from known mixtures, and the amount of the unknown is estimated from a standard curve. Laser NMAs can be used to identify either antigens or antibodies, yet they are usually run with antibody as the reagent and the case’s antigen as the unknown biomolecule. Due to their initial sensitivity, NMAs have been widely used especially for hsCRP. CRP quantitative nephelometry is a specific type of nephelometry performed to measure the CRP levels in a patient’s blood specimen, the test involves mixing the case’s blood with a reagent that stimulates any CRP molecule present in the blood to bind to latex particles, and these CRP-latex complexes cause light to scatter, that is counted by a nephelometer [22,1147,1148,1149,1150]. The main differences between the standard and more sensitive NMAs are the degrees of sample dilution and the calibration. NMAs have partially replaced the older Radial Immunodiffusion Assays (RIDAs) that were based on the typical precipitin reaction that antigen and antibodies react, creating precipitates in liquid/semi-fluid media.

Despite the fact that the basic principle of ELISA and radioimmunoassay RIA date back to 1941, earlier than TIA and NMA, ELISA was invented randomly by two research teams; it was developed in 1971 by modifying the radioimmunoassay, and it has been widely used in diagnostic microbiology since then [1151]. The antigen used in ELISA is bound to a solid phase, the enzyme-substrate reaction is completed in <1 h (the reaction can be stopped using alkaline media), and the results are read on a spectrophotometer [1151]. ELISAs can be homogeneous or heterogeneous methods, with the former being expensive and having low sensitivity, while in heterogeneous methods, washing to separate the bound antigen from the free antigen after the antigen–antibody interaction is required, and since heterogeneous ELISAs are more sensitive, they are frequently used. Direct antigen screening has a low sensitivity and can yield false-positives, compared to the indirect ELISAs, in which the antigen being measured is not the primary antibody, and is determined and separated by another antibody being placed in the medium, but immobilization in this situation can be non-specific [1151]. In sandwich ELISA, enzyme substrate is added to the medium, and coloration is ensured so as to reveal enzyme activity. Coloration shows a positive result, while a lack of it indicates a lack of enzymes (negative result). As the relevant protein is stuck between two antibodies, this method is known as sandwich ELISA and seems more sensitive than all other ELISAs [1151]. Finally, in competitive ELISA, the surface of the wells is coated with the antigen-specific antibody/antibody-specific antigen. The measured sample, the enzyme-tagged antigen or antibody are placed into the well randomly, and both the tagged and untagged patient antigen/antibody compete with each other to bind to the antibody/antigen in the wells. The wells are washed, and the enzyme substrate is added, and afterwards, the resulting coloration enables the quantification of the concentration that is inversely associated with the analyte concentration [1151]. The conventional and widely used sandwich antibody-based ELISA format has been critically simplified and improved recently; the leach-proof binding of capture Ab via ionic and hydrophobic interactions is attained, resulting in very highly sensitive ELISAs that are better than conventional ELISAs and covalent Ab-immobilization-based ELISAs, and the procedure detects CRP in diluted whole blood, serum, or plasma with high precision and specificity [1147].

Moreover, there exist other methods with comparable specifications, pros, and cons, such as the Chemiluminescent Immunoassays (CLIAs), and according to the different markers, CLIAs also include chemiluminescence enzyme immunoassays and electrochemiluminescence immunoassays. CLIAs can be direct (luminophore markers) or indirect (enzyme markers), and either method can be competitive or non-competitive [1152]. CLIA shows many similarities with ELISAs, such as the use of enzymes like peroxidase for antibodies labeling and the expected typical users. Nevertheless, the enzyme turns into a substrate in the chemiluminescent compound in CLIAs instead inducing coloration, which is typical in ELISAs, and additionally, increased sensitivity can be expected for CLIAs compared to ELISAs, yet market CLIA test kits have various sensitivities [22]. CLIA was designed in 1977 and based on the principle of radioimmunoassay, and was established by combining the significantly sensitive chemiluminescence with the highly specific immune response [1152].

Fluorescence immunoassays (FIAs) have the advantages of high specificity and sensitivity. The method uses fluorescein-labeled antibodies/antigens as tracers, similar to ELISA, and is the same as that of radial immunoassay. The basic difference between chemiluminescence and fluorescence is that the former results in light production, whereas the latter involves the absorption of light/electromagnetic radiation. FIAs can qualitatively and quantitatively identify antigens/antibodies in liquids and tissue sections, but due to the autofluorescence of samples and reagents and the excitation light scattering, the background fluorescence is raised, affecting the sensitivity of the assay [1153]. FIA types include the complement type, in which it is easy to produce non-specific interference and requires more controls [1153]. A specific FIA for CRP is based on an RNA aptamer, which selectively binds to mCRP but not to pCRP and forms a basis as a fluorescent anisotropic immunoassay for detecting nanomolar CRP, yet it is presumed to identify the CRP epitope located at the contact points between the protomers, thus rendering it unusable for pCRP detection [1147,1154]. Chemically modified thermoresponsive copolymers with specific ligands for CRP and fluorescein can find CRP at levels as low as 20 μg/L, and also, a two-step magnetic sandwich FIA on a multilaminar flow platform detects serum CRP in 1min with a sample of 10 μL. Moreover, generally, FIAs have a wider dynamic range compared to ELISAs [1147].

With the exception of the typical classic methods that cannot be performed in non-laboratory facilities, some other point-of-care diagnostic assays are used for CRP and various other biomolecules, and were widely performed even for self-diagnosis purposes during COVID-19 pandemic [1155]. Initially, Rapid Diagnostic Tests (RDTs) were recommended by WHO mostly in research, but nevertheless, low-cost technologies that have a high degree of accuracy, rapid turnaround times, and that can be implemented even by inexperienced laboratory staff and simple citizens, have become widely accessible for clinical practice. Most RDTs are designed on the basis of Lateral Flow Immunoassay (LFIA), and they are currently used for the qualitative and, to some extent, quantitative detection of various biomolecules (antigens, antibodies, other proteins, RNA, and DNA) in public or private non-laboratory environments [1,2,3,4,5,1132,1155]. The full quantification of LFIAs is not common, and semiquantitative assays based on a colorimeter and a lateral flow test are probably greatly improved compared to the qualitative LFIAs using the solely naked eye [22]. CRP LFIA-based RDTs have been presented as devices that consist of prefabricated strips of a carrier material with dry reagents, which are activated when applying the recommended specimen with the target biomolecule [2]. The RDT device employs a disposable test strip comprising an anti-CRP antibody bound to a nitrocellulose membrane, a sample pad, and an adsorption pad, both of which are placed onto a backing card, and the test and control lines on the immunochromatography test strip are bound to anti-CRP Ab and anti- rabbit IgG [1147]. Most of these test strips are based on the relative color appearance in the test and control lines from the competitive binding of anti-CRP capture Ab bound to a nitrocellulose membrane with the CRP-dye conjugate and free CRP in the specimen, and on the contrary, others are based on the formation of a sandwich immune complex from the anti-CRP capture Ab bound nitrocellulose membrane, CRP in the sample, and AuNP-labeled anti-CRP detection [1147]. When compared to the previously discussed methods, LFIAs depend on the subjective scaling of coloration; the colored lines are either formed or not visible at all [22]. The currently available commercial CRP kits based on LFIAs typically detect between single- and double-digit mg/L concentrations of CRP. Their sensitivity is relative, and typical standard relative errors are around 15% with an interassay precision around 20% [22]. Their major advantages are the overall simplicity (one-step assay with no further specific manipulation) with the tested samples as well as the fast procedure (finished in < 15 min).

### 5.2. C-Reactive Protein and Possible False Test Results in Common Diagnostic Assays

Even if certain assays seem to be the gold standard in molecular diagnostics, no method is completely foolproof, and in this manner, immunoassays are principally affected by antibody-related parameters. Some preanalytical errors occur due to bad sample, poor sampling, or poor preparation; contamination; as well as identification and transportation issues. Poorly trained laboratory personnel are also a risk factor for possible erroneous test results. Furthermore, some exogenous analytical errors include incorrect or degraded calibrator/reagent, lots of reagent variation, incorrect control constitution, pipetting issues, poor washing, inadequate temperature, or undetected bubbles. Type 1 endogenous analytical errors include hemolysis, icteria, and lipemia, whereas type 2 endogenous analytical errors are mainly antibody-related (interference) issues [1156].

Disorders such as Glucose-6-Phosphate Dehydrogenase Deficiency (G6PDD); CLD; diabetic ketoacidosis; alpha-thalassemia; hyposplenism/asplenia; lymphoma; and generally, anemia; are characterized by the presence Heinz bodies, which are clumps of damaged hemoglobin mainly found on the surface of Red Blood Cells (RBCs), and they have an impact on spectrophotometric measurements, falsely increasing the results—in relation to RBCs and hematocrit—yet there exist concerns for such interference scenario that have been reported for CRP diagnostics, which several CRP test kit manuals highlight in their limitations for this parameter [935]. Lipemia is caused mostly by chylomicrons > VLDL > LDL > HDL (stratified by their causal effect), and lipoproteins can interfere with antigen-antibody reaction by blocking the binding sites of antibodies even when they are bound to solid surfaces, while depending on the nature of the reaction, the interference can result in either falsely elevated or falsely decreased results [1157]. Indeed, old data reveal that serum lipoproteins and triglycerides can cause false-positive CRP test results [1158,1159]. Not only hemolysis and cholesterol/triglycerides, but also conjugated and free bilirubin can result in misdiagnoses [950]. Again, there exist an old case report on false-positive CRP test results in a case with hepatic cirrhosis, as revealed by a latex agglutination method [1160]. Since hemolysis, lipemia, and icterus can cause measurable spectrophotometric changes in clinical specimens, automated chemistry analyzers routinely detect these possibly interfering substances; thus, most clinical specimens for chemical analyses are prospectively assessed for these potential interferences [1161]. Generally, the upper values of hemolysis, lipemia, and icteria do not interfere with the assay and are reported in each test kit manual, and these values vary amongst different assays and test kits, so no comparison can be performed; in this way, a sample can show more precise results in one test kit, but interference can occur in another test kit. Even if some data highlight that the previous factors can possibly falsely raise CRP values, it has also been discussed that specimens with any sign of hemolysis are not acceptable for immunoassays of relatively labile analytes due to the release of proteolytic enzymes from RBCs that degrade such analytes (calcitonin, parathyroid hormone, and gastrin) [1162]. It was also discussed that not only collagen and lactoferrin, but also hormone binding globulins, such as albumin (because of its large concentration), sex-hormone-binding globulin, thyroid-binding globulin, and cortisol-binding globulin can alter the measurable analyte sample level either by the removal or blocking of the analyte [1,2,1162].

Generally, serologically speaking, the most profound cause of a false-positive test is a state of hyperglobulinemia in the clinical sample of an individual under consideration, which is the most common cause of a false-positive test, in other words, the “sticky serum” [1162]. A common type 2 endogenous analytical error in immunoassays seems to be the presence of Heterophilic Antibodies (HAs) in the clinical specimen, which are weak multi-specific low-affinity IgAs, IgMs, and IgGs produced against poorly defined antigens by most people and can arise naturally in the body as the result of antigen diversity; some of them may interact with self-antigens, and they can result in interferences in immunoassays by noncompetitive binding mainly to the Fc region of the assay’s antibodies [1,2,1156,1157]. It has been suggested that antibodies must be called heterophile when “there is no history of medicinal treatment with animal immunoglobulins or other well-defined immunogens and the interfering antibodies can be shown to be multi-specific (react with immunoglobulins from two or more species) or have a natural rheumatoid factor activity” [1163]. When HAs are present, it is very difficult to estimate the direction and magnitude of the interference that may be more common in competitive immunoassays. It is of importance to mention that the same antibody may react differently for different antibody combinations, thereby causing falsely elevated results in one assay but lower results in another assay, and immunoassays’ manufacturers typically add blocking agents (nonimmune globulins of various species) to their assays to saturate and reduce or eliminate the effects of HAs; however, not all HA interference can be prevented by such agents [1163]. However, generally, linearity in TIAs might still be observed even in the presence of interfering antibodies [1164]. There exists a report on false-positive CRP test results due to HAs in two children with uncomplicated renal transplantation, as found by a TIA [1165]. Heterophilic antibodies are usually absent in urine [1161,1166]. Even if some literature data define Human Anti-Animal Antibodies (HAAAs) as HAs, older data highlight the opposite, but they can also interfere with immunoassays and generally have higher avidity than HAs [1161,1167]. An example of HAAAs are Human Anti-Mouse Antibodies (HAMAs), which are much common; HAAAs can be produced due to medication with certain related monoclonal antibody drugs, frequent interaction with animals, exposure to animal products, etc. [1,2]. An ELISA test kit manual for detecting hsCRP notes in its limitations that inaccuracies can occur due to HAAAs, and such mention can be seen in various immunoassay test kit manuals [1168]. HAs can also occur due to blood transfusion, multiparous women (and pregnancy in general), maternal transfer, hemodialysis, and transplants [1169,1170,1171,1172]. CRP can be elevated post total knee arthroplasty, but if false-negatives are suspended, it may again be due to HAs post-arthroplasty or other surgeries that can yield HA production due to foreign surgery-related antigens [1173]. Other potential sources of HAs can be infections (EBV, CMV, HIV, etc.), autoimmune-supposed antibodies and other chronic inflammatory conditions, and immunization; particularly for autoimmune-related issues, Rheumatoid Factor (RF) is broadly classified as an HA for which numerous false positives have been recorded, and the current literature also reveals false-positives for CRP testing in TIAs and NMAs [1,2,1174,1175,1176,1177]. Although polyclonal immunoglobulins are more likely to cause interferences in immunoassays due to the so-called paraproteins, which are usually monoclonal but can also be polyclonal intact immunoglobulins or immunoglobulinic fragments (frequently light chains but also heavy chains) frequently produced by a malignant cone of B cells. The mechanisms for interference due to paraproteins depend on their unique properties that determine their conformational changes under particular conditions of each immunoassay; therefore, they can show antibody-like activities binding to analytes/reagents while also behaving like HAs, causing false test results, or like a cryoglobulin that induces RBCs to agglutinate, leading to incorrect hematology results [1156,1161]. It is obvious that sometimes, HAs and paraproteins are actually the same clone of immunoglobulins, but paraproteins can be present in all clinical specimens and tissues, whereas HAs are usually absent in urine [1161,1166]. Paraproteinemia has a high prevalence in the general population, and it is the most common cause of spurious or pseudohypophosphatemia. It has been reported in plasma cell dyscrasia, multiple myeloma, demyelinating (polyradiculo)neuropathy, amyloidosis, Waldenström macroglobulinemia, plasmacytoma, lymphoma (usually B-cell non-Hodgkin’s lymphoma), mediastinal mass conditions, chronic lymphocytic leukemia, and monoclonal gammopathy of either renal or undetermined significance. Undeniably, sometimes, either extreme acidic or alkaline pH conditions stimulate protein conformational changes, which promote protein aggregation, and most reported paraprotein interference cases have been reported in assays with extreme acidic conditions, like inorganic phosphorous, iron, and direct bilirubin, or extreme alkaline conditions, such as creatinine, total protein, and lithium. One could argue that acidic samples from cases with critical inflammatory medical conditions can possibly trigger such aggregation effects—even at a lower extent. In reality, few paraproteins interfere with any particular assay, and since assays on divergent platforms are not designed to be exactly the same, interference showed by one paraprotein with a specific assay on one platform may not be duplicated on another. That is, because proteins tend to precipitate at their isoelectric points, they therefore have various set points for conformational changes in response to pH and ionic strength. Paraproteinemia can also be caused due to cryoglobulinemia, in which cold-sensitive antibodies tend to precipitate in blood vessels, and except for myeloma protein, some other abnormal paraproteins can cause similar results. Generally, cryoglobulins are common, and they have been classified in into three types: Type 1 (simple) includes any of monoclonal IgG, IgM, IgA, or Bence Jones protein/monoclonal free light chains that have been associated with Waldenström ’s macroglobulinemia, multiple myeloma, monoclonal gammopathy associated with lymphoproliferative disorder, and light chain disease. Type 2 (mixed) includes any of monoclonal IgM, IgG, IgA, or polyclonal IgG that are linked to Hepatitis C, essential cryoglobulinemia, Sjogren’s syndrome, rheumatoid arthritis, and chronic lymphocytic leukemia. Type 3 (mixed) includes the polyclonal immunoglobulins of all isotypes linked to essential cryoglobulinemia, Sjogren’s syndrome, SLE, viral infections (HBV, HCV, CMV, EBV, and HIV), endocarditis, other bacterial infections, and biliary cirrhosis [1178]. Again, it is evident that some cryoglobulins can be the same HAs as well. There are several literature data examples reporting on such interferences in CRP and other tests; there also exists reports on a case with malignant lymphoma with paraproteinemia of monoclonal IgA-κ and another with chronic viral hepatitis type C with type II cryoglobulinemia composed of monoclonal IgM-κ and polyclonal IgG, which have been revealed to cause falsely elevated CRP test results [1176]. Two common interfering factors in TIAs are immunocomplexes or agglutinating immunoglobulins, which react with a chemical component like polyethylenoglycol in the reagent of the first reaction, causing remarkable turbidity in the initial phase, and as the turbidity is gradually reduced and cannot be eliminated within the first reaction, the second reaction is influenced by a continuing decrease in absorbance, leading to falsely low CRP values [1176]. Furthermore, a study reported that two different patient sera, one (chronic hepatitis C) with Waldenström’s disease and the other (purpura) with polyclonal hypergammaglobulinemia, had marked discrepancies between their CRP results, and it was found that these discrepancies were caused by milky turbidity produced by the non-specific reaction between high-molecular-weight components, referred to cryoglobulin, composed from IgM-IgG in Waldenström disease and immunocomplexes in polyclonal hypergammaglobulinemia [1179]. It has been reported in the literature that CRP NMAs showed interference by monoclonal immunoglobulin, as well as by monoclonal IgM-κ, in a case with myeloma [1180,1181]. Another study reported falsely elevated CRP values in a case with monoclonal gammopathy, and that paraprotein-induced latex particle agglutination also caused raised sample turbidity, thus leading to a false increase in CRP levels. The monoclonal gammopathy of undetermined significance resulted in falsely elevated CRP values in a case report, whereas an IgM-λ paraprotein was revealed to trigger falsely elevated CRP levels in an automated immunoassay using goat anti-CRP antibodies [1182,1183]. Of note, a known interference in a mother can be seen in her newborn [1156]. It is apparent that falsely elevated test results can be more frequent than falsely low results as most assays are taking into account the issue of high sensitivity rather than that of high specificity [1]. Also, false positive results in a sandwich assay format are more common since the interfering antibody acts as a bridge between the capture and detection antibodies [1156]. Moreover, sensitivity and specificity are inversely proportional, and as sensitivity raises, specificity decreases, and vice versa [1142].

Other type 2 endogenous analytical errors in immunoassays that can lead to inaccuracies include the existence of antibodies against revelation systems (i.e., anti-ruthenium antibodies presented in clinical specimens); immobilization system interferences (i.e., biotin and possibly anti-biotin antibodies particularly for ELISA, and other immunoglobulins, such as anti-streptavidin, anti-bovine, anti-goat, anti-rabbit, anti-complement antibodies presented in clinical specimens, etc.); and cross-reactivity due to various factors including medication and its metabolites, which can persist in the body for several hours/days, diseases, and lifestyle (i.e., overconsuming specific foods or beverages such as alcohol/certain juices or consuming them just before sampling can cause LFAs to produce a false-positive test result in some analytes, but their mechanism of action is mostly affected by acidic specimens) [1,2,1156,1161,1167,1184]. Particularly drugs prompting the most alerts have been frusemide, acetaminophen, penicillin, and hydrochlorothiazide mostly for total leukocyte count, as well as hemoglobin, potassium and glycose tests, but that does not mean that some other assay types for other biomolecules are always 100% accurate for these or other drugs [1184]. Indeed, a CRP TIA manual highlights that significantly decreased CRP values may be obtained from specimens taken from cases who have been treated with carboxypenicillins [1185]. It was also discussed that treatment with radioactive or fluorescent compounds, drugs, herbal medicines and nutritional supplements can adversely affect immunoassays. In cross-reactions, which is a problem in diagnostic immunoassays, endogenous molecules that are structurally similar to the measured analyte exist, where metabolites of the analyte have the same cross-reacting epitopes and when a structurally similar drugs is administered [1162]. A factor that can result in cross-reactivities can be medication with various monoclonal antibodies; it is evident that such therapies increase the overall serum immunoglobulin load, and such a factor should be assessed in case of possible interferences and cross-reactions [1,2,3,1042]. Also, specific factors in sputum, BAL, urine, and stool sample analysis that can possibly interfere and cause erroneous test results have already been demonstrated. for instance, inhaled toxins or chemicals in large amounts or just before sampling can affect test results [1,2]. No interference is reported in some test kit manuals for citrate, EDTA, ascorbic acid, fluoride, or heparin up to certain concentrations, but for higher levels, data are absent [1186,1187]. So, for such cross-reactions, it is obvious that not only overconsumption/overuse can trigger false results, but also normal consumption/usage just before sampling can have similar effects. Additionally, it is generally possible that fibrin generated from residual fibrinogen in the case of inadequate post-phlebotomy tube homogenization can cause interferences in some immunoassays [1156]. Moreover, inefficient mixing can also yield reduced stability in a sensitive measurand, but, on the other hand, anticoagulants do not have an impact on serum samples as they do not require any and show a good stability for most biological measurands; yet, this type of tube needs a clotting phase of 30–60 min before centrifugation to diminish fibrinogen, fibrin, and blood cells that may interfere, thus prolonging the global TAT [1156]. Importantly, it should be highlighted that in cases presenting anti-CRP antibodies, false-negative test results may be evident in case samples with poor washing, which can lead to immunocomplexes between CRP and anti-CRP antibodies, thus lowering free CRP levels in the final analyzed sample, and apart from such immunocomplexes, it was also discussed that CRP can bind to soluble receptors or exist in plasma microvehicles. Thus, poor washing, again, can lead to false test results. It was addressed that autoantibodies have been described causing interferences for various analytes, and either a positive or negative influence may be seen, depending on whether the autoantibody–analyte complex partitions exist in the free or the bound analyte fraction [1162]. Moreover, it has been stated that in reality, all human beings present autoantibodies interfering in immunoassays (specific IgG4 anti-immunoglobulin autoantibodies) [1,2]. Furthermore, in CRP carbamylation, there is a nonenzymatic post-translational modification seen during the reaction between cyanate and amino acids and/or proteins that can occur in some pathologies (i.e., CKD), and elevated cyanate concentrations can give rise to falsely decreased CRP levels, as measured by TIA [1188]. Importantly, the so-called Hook effect, also known as the prozone effect, antibody excess, or the prozone phenomenon, occurs when the target antigen/antibody is in greater concentrations than those of the capture antibodies and vice versa (i.e., when the capture antibodies are very low levels compared to the target), and it can trigger false-negative test results in several assay techniques, including one-step immunometric assays, TIAs, NMAs, ELISAs, LFIAs, and other immunochromatographic assays [1156,1160,1189,1190,1191]. Finally, CRP itself may cross-react or interfere in other assays, triggering false test results. Erroneous test results in lupus anticoagulant in antiphospholipid syndrome because of CRP have been reported in the current literature [1192].

An important issue is that each immunoassay has its own Limit of Detection (LoD) score; it is obvious that low LoD score values can possibly be the cause of false-positives, whereas higher values can result in false-negatives, and, depending on each specific case’s medical history, such value could yield erroneous test results. Some test kit manuals report that each laboratory establishes its own reference range [1193]. Further assay-specific parameters can be seen in each test kit manual, yet in TIAs and ELISAs, and some other methods, some reactions that last longer than determined may produce obvious false reactions because of a drying effect, while the strength of the agglutination does not indicate CRP concentration, as weak reactions can occur with slightly raised or markedly elevated CRP concentrations. Also, laboratory factors, such as linearity, which has a standard upper value in each kit, should be taken into account, but it was previously discussed that interfering antibodies can show linearity, too. The prozone effect can take place in significantly elevated values that vary amongst test kits (>50 mg/L in an hsCRP test kit; >250 mg/L and even >800 mg/L in some CRP test kits; etc.). Therefore, when there are suspicions for notably raised CRP values, dilution in saline before sample analysis is required so as to have more accurate results; yet, linearity can have significantly lower values [1193,1194,,1195,1196,1197]. The upper reportable value is in reality virtually unlimited, but the upper limit of some assays’ default dilution is determined by the calibration material that is supplied by the manufacturer. Furthermore, NMAs may be subject to interferences caused by serum microparticles or pigments (i.e., lipemic or icteric samples) that increase background light scatter and lead to difficult or impossible result interpretation; therefore, NMAs measuring scattered light in a more forward direction provide greater sensitivity and will be less impacted by interferences from endogenous proteins, chylomicrons, HDL, LDL, and aggregated immunoglobulins, specifically after freezing/thawing serum samples. Extra freezing and thawing cycles (a practice that can lead to sample pathogenic contamination) can give rise to erroneous test results, and in order to avoid them, specimens should be aliquoted, and also, the heat-treatment of clinical specimens can be attributable to false results. Yet, the contamination of assay reagents and disposables by particulate matter, especially dust and lint should be avoided, and increased serum lipids can interfere with nephelometric determinations; thus, a centrifuge may be required [1198]. A homogeneous CLIA CRP test kit manual highlights that abnormal clinical samples should not be further analyzed, and also, those from cases with liver disease may result in less pronounced measures, while another CLIA test kit protocol notes that insufficient sample washing can lead to falsely elevated test results [1199,1200]. Sometimes, in various test kit manuals it is highlighted that the testing of human serum used in the preparation of the standard produces a negative result for the presence of anti-HIV (1 and 2) or anti-HCV antibodies, or HBs antigen, and because of the possibility of being infectious, standards should be used cautiously. This does not necessarily mean that a positive sample for those antibodies/antigens is always safe from potential cross-reactions since several antibody tests during the pandemic revealed cross-reactions and misleading test results for such antigens/antibodies [1,2,1185,1201]. Finally, it is worth highlighting that some tests may be not able to detect CRP mutants; thus false-negatives may arise, or the opposite. Yet, even if a test detects a CRP mutant, it remains unknown if this mutant can behave like the original CRP in human body or simply exist with no real activity or in degrading states.

## 6. The Expert’s Opinion: A Critical Appraisal of the Current Literature on C-Reactive Protein and the Mysterious Systemic Inflammation

The current literature seems to be at odds; numerous data on pathobiology and the diagnostic spectrum of CRP seem to have various types of bias, and the quality of the methodologies of most diagnostic studies is generally poor, with inconsistencies and preliminary analyses leading to conflicting results. In contrast, molecular diagnostics have not always been the golden desideratum. Since molecular diagnosis for the identification of CRP precedes, it seems scientifically wise to unravel the knowledge beginning from molecular diagnostics.

Laboratory immunoassays consider serum CRP as an antigen that needs to be detected, and also, anti-CRP antibodies have been reported in some conditions. The “antigen” is derived from antibody generation, and refers to any substance that is capable of inducing an immune response (i.e., antibody production); thus, it binds with the specific immunoglobulins formed by its presence. The strength of an antigen–antibody interaction can be described by their affinity, i.e., the equilibrium constant that describes the antigen–antibody reaction, whereas within various antigenic sites, each antibody arm (the variable region) interacts via weak noncovalent forces with numerous antigenic sites, and a stronger affinity is actually a greater interaction; avidity is a more illustrative measure of the overall strength/stability of the immunoglobulin–antigen complex, and it is mainly modulated by antibody epitope affinity, the valence of antigen-antibody, and the structural arrangements of the interacting parts, which all define an antibody’s specificity, which is the likelihood of the particular antibody–antigen binding [1202,1203]. Weak blood interactions may be evident since it has been proven to interact with various ligands/receptors. Affinity maturation and the seemingly impossible task of recognizing a potentially infinite epitope repertoire with a finite antibody paratope array are fulfilled by the regulation of the conformational flexibility of the antigen binding site. An early high flexibility allows for cross-reactivity but at the expense of low affinity due to the unfavorable entropy alterations (antigen binding entails a conformational freedom restriction), and conversely, elevated rigidity abolishes cross-reactivity but entails favorable entropic changes during antigen binding, although enthalpic factors can be involved [1204]. Indeed, it was previously discussed that the Ca^2+^-binding region on the CRP surface has substantial flexibility and is possibly responsible for the allosteric effects of Ca^2+^ ions on CRP, further regulating the binding of CRP-specific monoclonal antibodies [3,63]. Moreover, low avidity or specificity of the antibody or by more distinct antigens sharing identical or very similar epitopes are attributable to cross-reactions [1202,1203]. Antigens and antibodies are multivalent, and interactions involving multivalency can result in more stabilized complexes but also in steric difficulties, which reduces binding possibility. Antigen–antibody binding obeys the basic thermodynamic principles of reversible bimolecular interactions, and the time needed to reach equilibrium is widely variable and depends on diffusion rate and affinity, which is affected by temperature, pH, and the solvent, whereas affinity constants cannot be determined for multivalent molecules due their capability of multiple bond formations. In the case of ionic strengths or pH alterations, such as in inflammatory acidic microenvironments, it is possible that antibody conformational changes (including possible partial unfolding) affect antigenic complementarity; thus, cross-reactions may occur, and various aggregated immunocomplexes may be evident. The extent of such immunocomplexes—referring to protein-based complexes—depends on the extent of the initial acidosis that has a high entropic cost since acid ionization raises the number particles, and such increase leads to chaos. This is why it is noted in Figure 4 that CRP can bind to any near proteinic structure in acidic inflammatory microenvironments.

Similar biochemical reactions occur in immunoassays; it is already known that immunoassays are affected by the overall serum Ig levels. Serologically speaking, the most profound cause of a false-positive test is a state of hyperglobulinemia in the serum or plasma of the individual under consideration, which is the most common cause of a false-positive test, in other words, the “sticky serum”. Therefore, it is obvious that contrary to the current state of research on sandwich immunoassays, falsely elevated test results can be more frequent than falsely lowered results as most assays are taking into account the issue of high sensitivity rather than that of high specificity. The current literature reveals that CRP interacts with multifarious types of ligands and receptors; thus, there may exist various sample complexes, and therefore, there is a greater possibility of falsely raised test results. For instance, most people have now been infected by SARS-CoV-2 or have been vaccinated against the disease, thus resulting in chimeric spike protein transcripts, which lead to further antibody production. Thus, perhaps the serum proteinic (antigen–antibody) load is raised, and more immunocomplexes may be present, and given the fact that CRP can bind to the spike protein RBD motif, it is possible for falsely elevated test results in immunoassays to be more common in the post-pandemic society [1,2,91,102,1042]. In this manner, even if test kits with anti-mCRP antibodies are currently not commercially available, it is unknown whether such cross-reactions and proteinocomplexes including CRP types (mCRP, pCRP, and decameric CRP and its polymers in general) are really absent. It was also discussed that certain drugs and, generally, several other substances can affect a test’s results, but referring to these interfering factors, we cannot know what exactly exists inside the patient’s organism at a microenvironmental level, so as to predict possible misdiagnoses; therefore, it may not be possible to avoid inappropriate treatment [1156]. Moreover, various CRP polymorphisms and mutants will result either in adverse or favorable responses in acidic inflammatory microenvironments. Thus, a possible positive test result may actually mean the opposite; in other words, we cannot know if the detected molecule is really active in a specific individual. Moreover, the various previously discussed CRP test results in certain conditions reveal possible antibody interference-related discrepancies; for instance, one could argue that some raised CRP levels in autoimmunity could just be antibody interferences since cases with autoimmune conditions are supposed to have higher serum immunoglobulin load. Also, certain reports on COVID-19 fulminant myocarditis presented with significantly low serum CRP levels, possibly showing that either COVID-19 or CRP test results have potentially been affected by antibody interferences, or maybe that pCRP is rapidly converted into mCRP, which is not detected; thus, a low CRP level is diagnosed. Also, in HCV/HBV infections, it was noted that CRP levels were notably decreased, but how can an infection be in parallel with low CRP, which is supposed to be an acute phase reactant? Again, could rapid dissociation into the monomeric form have led to reduced pCRP levels, could false viral positivity have occurred, or maybe could CRP immunocomplexes with anti-CRP antibodies that have been reported in HCV infection have led to such misdiagnoses?

Furthermore, most studies report various CRP test results, and there is no evidence regarding which exactly immunoassay was performed nor for which specific test kit was used. Thus, we cannot know the test’s limitations from its kit manual or the factors that can possibly trigger an erroneous test result. In this way, despite the fact that large studies vary substantially in cut-offs for normal/abnormal CRP ranges, no comparisons can be performed for CRP values obtained by different methods and different test kits with different limitations, and it is obvious that even results showing the same CRP value but that were obtained by different immunoassays/test kits can, in reality, refer to different CRP concentrations in the corresponding clinical specimens. Except for all the previously analyzed factors and the differences in assays/test kits that can affect CRP concentrations, hsCRP assays from different laboratories have notable discrepancies in reported results, underscoring the need for further standardization. Importantly, if certain clinical samples with possible elevated CRP require dilutions so as to prevent the Hook effect, it is obvious that each test kit has its method and its buffers, and subsequently, the final CRP values will depend on these specific sample manipulations, which will be based on a specific test kit method and its buffers, and as a result, these values cannot be compared—except if a comparison occurs for samples diluted with the same buffer of the same test kit. Moreover, studies report CRP concentrations in mg/dL, whereas others report in mg/L, resulting in further discrepancies. Also, most studies report CRP, while others report hsCRP, and even if actually it were the same molecule, different methods (that are more sensitive for hsCRP) can lead to different results; thus, again, no comparisons can be made. Therefore, the aim of this review is not to compare various CRP values or predict potential ranges in certain medical conditions. Apart from all previously discussed issue, to my knowledge, there are no data and there exists no accurate and thorough study comparing CRP values by different testing platforms (using the same samples) so as to provide evidence on potential falsely elevated/lowered values and their possible etiologies. Additionally, there are no studies reporting the rates of possible false test results due to specific foods or medications; thus, it cannot be estimated in daily clinical practice. Doubtlessly, such studies are a must and should be conducted in the near future.

Nevertheless, there is no point in describing precise CRP concentrations since interferences are affected by various exogenous or endogenous sample factors. It was previously discussed that sensitivity and specificity are inversely proportional, i.e., as sensitivity raises, specificity decreases, and vice versa [1042]. Various methods and test kits have different cut-off values, but the use of a higher cut-off can increase specificity, with a lower cut-off sensitivity that can also be increased. Currently, the application of CRP in the clinical assessment of any single disease has been hampered by several issues, especially its lack of specificity. Since test results are affected by multifarious factors that mainly depend on each particular individual, there is no discussion on positive and negative predictive values for each assay. One could argue that the ideal cut-off value would be unique for every case in a certain test kit. Not only the various cut-offs, but also the different upper detection limits in linearity can be confusing in reversed ways. In addition, it is impossible to identify the precise baseline normal CRP levels since there exists so many factors influencing CRP concentrations and production as well, and up to now, no published study has taken all these parameters into account so as to draw more unbiased and accurate conclusions regarding baseline CRP levels. All in all, the aim of the test result assessment should be the real presence or the absence of CRP in a patient and not its precise concentrations, which are affected by multifarious parameters—in this scenario, I am speaking about a type of test with binary results.

Moreover, even if mCRP or pCRP are detected, we cannot know if these molecules in a random clinical sample are, in reality, totally active or if they are simply proteinic fragments undergoing degradation (inflammation clearance). Thus, it is not at all possible to obtain any significant results in this case; in other words, we cannot hypothesize what actually occurs inside the patient. The supposed presence of mCRP in blood means that pCRP turns into the monomer under some previously discussed circumstances, and the real measured CRP concentration depends on the rate in which the pentamer converts to mCRP, but again, such transformations depend on various individual factors, and even if some authors propose that the ratio of these two forms could be useful, in reality, it will be more confusing because more recent data report several other CRP forms (dimers, trimers, decamers, and polymers). Additionally, it was discussed that standard clinical hsCRP immunoassays cannot detect mCRP or urea-solubilized mCRP on microparticles; therefore, hsCRP diagnostics can only measure total pentamer percentages. It is believed that in the earlier stages of tissue trauma, the conversion of pentamer into mCRP is efficient and rapid, and that the monomer has potent proinflammatory bioactivities, but after that stage, this conversion slows, leading to raised blood pCRP, and since mCRP is rapidly formed and sequestered into membranes and inflamed tissues, its solubility is reduced. It is thus difficult to be detected in body fluids compared to pCRP, which is supposed to show weak proinflammatory activities, and therefore, it is easy to be detected in blood [63]. Moreover, mCRP is supposed to be rarely found in circulation through available quantification methods, suggesting a possible predominance of local conversion [26]. Yet, it is unclear how the so-called monomeric form accumulates in tissues as it may cross the endothelial barrier after dissociation or be synthesized locally. This problem of local mCRP formation has been explored in various studies, but the contribution of local conversions to the total mCRP in tissues and bloodstream is unknown because, up to now, most research refers to CRP and does not distinguish between the various isoforms (not only mCRP or pCRP, but also monomers and polymers) in other ways in CRP degradation. It seems worthwhile to mention that human blood is slightly alkaline, the isoelectric point of antibodies can be slightly alkaline or slightly acidic, whereas the CRP isoelectric point is lower, making it a negatively charged molecule in blood—compared to its charge alterations in acidic inflamed tissues [1205]. It has also been discussed that increased synthesis and plasma CRP are more likely to be related to tissue damaging pathologies rather than the inflammation itself [63]. Chronic unamplified inflammation can persist in such a situation, exacerbating tissue-destroying processes and provoking wound healing and repair to re-establish health homeostasis [53]. It was previously discussed that various inflammatory disorders can have raised concentrations, and some bacterial infections can occur in parallel with an up to 1000-fold increase in CRP levels. On the contrary, despite the fact that elevated pCRP levels may be needed so that more mCRP can be produced in acute stages, higher pCRP titers can also be a marker for insufficient or poor pCRP conversion into mCRP. In this way, how can pCRP be elevated in acute phases and be responsible for inflammation? It is evident that adding pCRP to tissue culture cells causes pCRP to convert into mCRP within the first four hours post pCRP addition [76]. Thus, evidence suggests that pCRP degradation is more feasible in healthy tissues (as they are possibly capable of its clearance). However, such laboratory conversions may be extremely variable in vivo as they depend on genetics, the extent of acidosis, and several other individual factors. Therefore, current evidence on possible CRP conversion from polymers to monomers and its clearance is somewhat controversial, and also, test results are more likely to trigger further diagnostic inaccuracies rather than determine the actual roles of CRP. As a result, such findings are somewhat unclear and at times conflicting as it is often unspecified which exact CRP form was measured or utilized in experiments, whether responses to any polymers were in reality due its partial or full dissociation into monomers. Also, the parameter of lipopolysaccharide and other contaminations in some experiments is not taken into account. More recent studies distinguish between mCRP and pCRP, but again, other CRP forms are not distinguished or taken into account as study limitations. An old hypothesis proposed that distinct CRP forms with unique activities are created in inflammatory sites, and that conformationally altered proteolytic forms are created from pCRP due to local conditions (i.e., lowered pH, ROS, or possibly enzymes) [1206]. Taking into account the isoelectric point of CRP and that it actually is very stable, the pH of the blood, as well as the various acidic inflammatory microenvironments’ pH, it seems that the release of separate protomers requires CRP exposure to harsh denaturing conditions, but indeed, there are no compelling data for the realistic persistence of such extreme acidosis and the subsequent denaturation of CRP in vivo, and the rapid complete catabolism of such biomolecules would be expected [64]. It has also been proposed that aggregated and/or conformationally altered CRP forms initially promote inflammation, and subsequently produce peptide products either up- or down-regulate different leukocyte activities to aid in the progression of inflammation [1206]. Nevertheless, such forms would hinder the binding of preferred CRP epitopes with their ligands, and also, the presence of a specific form does not always mean it is capable of and will induce further cascades at the specific microenvironment—thus, a false positivity and the misconception of the actual role of the CRP form may occur [1207].

Generally, nowadays, CRP is attributable to some functions that seem controversial and inherently unlikely; for example, it is improbable that a plasma protein with a dynamic range that is 10,000-fold greater can act as a cytokine within several hours or be a real regulator of sophisticated cellular or physiological systems and further mechanisms; even a sole toxin may act as a multi-regulator during inflammation and acidosis [64]. It seems impossible for such forms to be studied and their exact role to be clarified since it is supposed that currently, pCRP is only detected via a positive test result by no other means, and that a such a positive test result cannot lead to further conclusions on the actual existence and role of such altered CRP forms. Therefore, one could argue that the concept of monomers and pentamers actually refers to something like simple CRP degradation in vivo and not the so-called different molecules attributable to such various and controversial mechanisms. Therefore, obviously, various contrasted data provide inaccuracies toward the real role of CRP (i.e., the tumoricidal activity of CRP compared to CRP use as a predictor of poor prognosis in cancer patients). Moreover, even if it were valuable to detect the rate of CRP degradation, it seems highly difficult to identify active and inactive/fragmental CRP molecules. Figure 2 and Figure 3 show that both mCRP and pCRP functions are not completely contrasted and different as it was thought to, and this may occur, as it is summarized even in Figure 4, that CRP can actually bind to any proteinic structure in acidic inflammatory microenvironments. This is a very important point, and it highlights that CRP can actually bind to anything and present any function or no function at all, and due to this, undeniably, no direct CRP functions can be revealed. It must be highlighted that almost all studies published prior to the early 2000s do not include any awareness or possible relevance of distinctive structural and biofunctional CRP isoforms that possibly have influenced the data generated. Indeed, for decades prior to 2000, CRP literature was full of contradictory conclusions between seemingly identical studies, and any reader that accesses a reference from these earlier dates must be made aware that that data presented may be reflective of the at-the-time unrecognized inclusion of functionally distinct molecules.

In acidic inflammatory tissue conditions, CRP’s various aggregated forms/conformational alterations may result in its linkage with various types of ligands/receptors; therefore, such conditions could resemble the various immunocomplex conditions in vivo. It has been revealed that inflammation induced by immunocomplexes in microcirculation is host-mediated, and that the kinetics of such inflammatory reactions are similar to reactions initiated by other particulates or soluble stimuli [1208]. However, almost all studies assess CRP and its role as the cause of most diseases and do not bear in mind all the factors that are were discussed in Section 4.5 that actually have a great impact on the progression of a disease, and therefore, data regarding the role of CRP as a possible marker of most diseases seem controversial. Such conflicting results reported in low-quality studies are also biased because of various limitations in study designs, like a retrospective case–control design, prior undiagnosed medical conditions, medical history and time until the occurrence of the studied event, CRP measurement with various assays of different specificity/sensitivity/LoD scores/etc., different medication strategies, history of medications, relatively small study sample sizes, etc. It is obvious that hsCRP can be slightly elevated in a sample, that is more affected by the factors discussed in Section 4.5 compared to another sample that is less affected, regardless of the disease type. Thus, the aim of this review is not to compare hsCRP values in various medical conditions, as such values are assessed at an individual level—mostly based on these illustrated factors. This is the reason why the term “potential” is preferred in most headings of this review, because currently, almost nothing can be taken for granted.

Studies on autoantibodies against CRP seems sparse; however, it is already known that by detecting antibodies, one can identify the reaction and not the real action nor the condition. By taking anti-CRP antibodies as a real fact and not as a possible cross-reaction to anything else, it seems that the organism tries to fight CRP, and that maybe this molecule is, in reality, seen as a foreign antigen. On the other hand, since SLE and antiphospholipid syndrome cases also present anti-CRP antibodies, one could argue that these may actually be immunocomplexes that were not washed enough. Moreover, no study reveals the possibility of cross-reactions between anti-CRP antibodies and other biomolecules having potential similar epitopes that could actually bind to anti-CRP antibody paratopes—mostly in acidic microenvironments, even with weak interactions. It has also been suggested that CRP is capable of recognizing both self- and foreign molecules based on pattern recognition, something that other activators, like IgG, cannot achieve as immunoglobulins recognize solely distinct antigenic epitopes [80]. So, how does autoimmunity actually occur? Is it a sole cross-reaction condition by immunoglobulins that were initially produced against foreign antigens? And how are anti-CRP antibodies produced? Is CRP actually a foreign biomolecule that just cross-reacts with both weak and stronger forces with other molecules based on each particular acidic microenvironmental entropy, leading to further aggregated complexes (mainly proteinic forms/fragmental peptides/etc.)?

When the bacteriologist Oswald Avery (who isolated DNA as the material of which genes and chromosomes are made) joined the Rockefeller Institute in 1913, he directed a considerable effort in his lab to understand pneumonia and its supposed most common etiologic agent, Streptococcus pneumoniae, generally referred to at the time as the pneumococcus, and over the course of these studies, CRP was discovered, although one could rather say they blundered into it [1209]. CRP was discovered by Tillett and Francis in 1930 with a simple procedure, according to their description, and provided a material that was comparable in reactivity and specificity to more highly purified preparations; thus, no further purification steps were carried out. However, some lots were treated by repeated precipitation, and also, their illustrated approximate figures were believed to be conservative estimates since a quantitative estimation was not made on all lots of material [23]. Since no further purification was needed, why were some samples treated by repeated precipitation? Could CRP have been detected in lower levels in certain samples? One could hypothesize that all samples ought to be have been treated with the same steps so as to have a common denominator. It is worthwhile to add that a major limitation of mass spectrometry (that has been applied to determine CRP molecular weight) is that it cannot reliably aid in tracing the real origin of the tryptic peptides in order to determine which genes really code for the proteins that were detected in the analyzed sample, and also, the initial SDS-gel electrophoresis and AA analysis for CRP was based on a CRP purification by samples of cancer cases, which actually have a higher serum load of aggregated protein-/immunocomplexes, and some of them can be also lipid-bound. Thus, purification does not seem as easy as it was supposed to since such complexes have relatively recently been revealed in the literature and not a century ago [1210]. As a result, it is possible that similar limitations could be evident in those methods, or there may exist some other limitations since, in reality, no method is completely foolproof. The CRP gene was further sequenced and analyzed in genomic libraries, but again, several factors affect such procedures, including DNA contamination in particular. Indeed, computer analyses found no significant repeating sequences within CRP, an observation that seems to rule out the possibility of gene duplication during CRP evolution; nevertheless, statistically insignificant distant homologies, have been noted to IgG CH2 domain and to C3a anaphylatoxin, but such homologies are insufficient to support a common evolutionary origin, and also, no homology region in other heavy chains was revealed [1210]. Nevertheless, some studies indicate some CRP functions far away from those of immunoglobulins; thus, it may not directly behave as an antibody. Another question arises regarding such regions showing homology: could they trigger false-positive test results? The literature reveals that Streptococcus pneumoniae produces and attaches a variety of proteins to its cell surface, including choline-binding proteins, which are attached through noncovalent interactions of conserved choline binding domains with phosphorylcholine moieties in the teichoic acids of the cell wall or lipoteichoic acids embedded in the cell membrane [1211]. One could speculate that CRP could be such a protein, and indeed, it was initially thought that CRP was of bacterial pneumococcal origin. Indeed, two distinct regions of pneumolysin, a membrane-damaging toxin produced by Streptococcus pneumoniae that is known to activate the classical complement pathway, show homology with a contiguous sequence within CRP [1212]. Could these regions share common bacterial origin? However, since a CRP genetic locus has been detected, could this gene be of bacterial origin? It has already been proven that bacterial gene transfer to somatic cells is possible, and also, human chromosome 1, where CRP gene is located, (1q23) is the largest human chromosome [1213]. In 1928, the bacteriologist Frederick Griffith described the mutation of a non-pathogenic pneumococcal bacteria into a virulent strain; he had mixed the living non-virulent bacteria with a heat-inactivated virulent form [1214]. Nowadays, it is known that systemic inflammation triggers human DNA release, a fact that could possibly help natural bacterial transformation in vivo [1215]. Not only heat, but also the lower pH in acidic environments can affect natural bacterial transformation in the case where free DNA exists in the microenvironment; for instance, it is known that bacterial transformation is an important mechanism for bacterial adaptation to the human gastric environment [1216]. Cell apoptosis and necrosis and subsequent genome release are affected by various factors, as mentioned in Section 4.5. In this manner, one could argue that human non-pathogenic bacteria may transform into pathogenic forms due to systemic inflammation; thus, infection and subsequent disease is possible.

Nowadays, it is known that Streptococcus viridans is a group of Gram-positive, alpha-hemolytic streptococci that show genetic heterogeneity, and that they are commonly found in the oropharynx; epithelial surfaces of the oral, larynx, and pharynx (upper respiratory tract); all gastrointestinal tract, genital tract, but rarely in skin surfaces; and possibly other body places (normal human flora) [1217]. One could speculate that both pseudopneumoniae and nonpneumococcal viridans group streptococci, which are actually oropharyngeal colonizers and are not supposed to cause symptomatic infections, may be mutated/transformed in vivo in certain conditions or cause false-positives in test results. Again, no method is completely foolproof, and recently, it has been discussed that the misidentification of viridans group streptococci as pneumococci could cause an overestimate of antimicrobial resistance, and also, further unreasoned antibiotic use may result into mutants that may be capable of inducing pneumonia [1218,1219]. Streptococcus pneumoniae is a common asymptomatic colonizer of the human nasopharynx, but in vulnerable hosts, it can invade other niches, causing otitis media, conjunctivitis, meningitis, pneumonia, and septicemia. The current literature reveals that pneumococci are highly competent organisms, that their genome sequences show extensive signs of horizontal transfer of genetic material, and that natural transformation in the nasopharynx is facilitated by the co-colonization of multiple pneumococcal strains [1220,1221]. Therefore, the uncontrolled use of antibiotics can lead to more pathogenic strains, thereby causing infections. Thus, a sole positive test in asymptomatic cases can lead to unreasoned prescription of antibiotics that can trigger the creation of further more virulent strains and human disease. CRP was reported to be notably elevated in bacterial infections; acidic environments (systemic inflammation) may have triggered pathogenic and more virulent bacterial transformations, that can possibly lead to elevated CRP. Could there be a possibility of certain basophil bacteria being in parallel with elevated CRP since it is possible that in acidic inflamed tissues, such bacteria are degraded? The initial assumptions were based on the hepatic origin of CRP, but in reality, not only does the liver have bacterial colonies, but also the liver is considered to be the master and servant of the serum proteome [1222]. Also, it was discussed that even if CRP levels were related to various medical conditions, liver failure and certain medications affect CRP production. Except for the role of liver in the selective uptake, levels, metabolism, and excretion of most drugs and toxins introduced into the body, its main job within gastrointestinal tract processes is to process the nutrients absorbed from the small intestine; therefore, one could argue that in case of elevated liver post-digestion toxins, this organ boosts internal acidity. Thus, it would be logical for CRP to be initially produced in the liver. On the contrary, more recent data have revealed various other extrahepatic locations of CRP production, and actually, these places seem to be in parallel with the presence of such bacterial colonies. Another important question arises: CRP is supposed to be an acute-phase reactant with human origin, and data highlight that it is elevated in systemic inflammation, but why do viral infections that are believed to be more pathogenic and harmful show relatively lower CRP levels? One could argue that a false positive may have been produced, or that either viruses or CRP does not exist! Viruses are supposed to use human cells to replicate and proliferate, thus they need alive cells, and it is not their target to kill the host as they essentially need it in order to exist. Thus, the immune system ought to be less stimulated for CRP production. It is also known that bacterial co-infections are actually the etiology of poor outcomes in viral infections, such as pneumonia. Therefore, one could say that lower CRP levels may be produced, in reality, due to bacterial co-infection.

Despite several literature claims and assertions, the possible functions of CRP in healthy individuals are unknown since no deficiency or even structural CRP polymorphism has yet been reported, and no drug or other therapeutic maneuver is available yet, which particularly inhibits or depletes human CRP in vivo, and the possible adverse effects of absence, lack of function or inhibition of CRP have thus so far not been tested [188]. On the other hand, a genetic study found that excessive CRP is deleterious, and elevated basal CRP concentrations predict increased mortality; therefore, one could understand that in healthy individuals, CRP ought to not be produced [1223]. On the contrary, there are no data to suggest that CRP is the direct cause of any disease, whereas CRP infused in relatively healthy human adults does not trigger any significant clinical, hematologic, coagulative, or biochemical alterations, or any increase in proinflammatory cytokines or acute phase proteins [113]. Apparently, there exists no healthy individual with zero acidosis, so one is needed to identify the real role of CRP in vivo, and not in vitro. It must also be highlighted that CRP expression is significantly different in humans vs. mice, thus comparisons seem not to be as accurate [659]. However, microbiologists do not study in vivo, but rather the in vitro tissue death, precluding that such death is related to the overall lab-forced treatments and conditions, which are undeniably very different from both the in vivo human pre-disease and disease toxins and acidity, all of which contribute to the final large entropic cost for a living organism.

It seems impossible for all medical conditions to be discussed in one review article, but there seems to be a common denominator: acidosis and subsequent systemic inflammation. Science has proven that chronic low-to-moderate inflammation can be a silent killer, contributing to various medical conditions. It is estimated that conditions including bronchial hyperesponsiveness are based on systemic inflammatory processes and even atypical hypersensitivity reactions, which also trigger systemic inflammation. Indeed, even if the real role CRP seemed unclear, the previously discussed evidence highlights that CRP and several pro-inflammatory cytokines simply coexist; raised CRP and IL-6 concentrations in diseases such as paroxysmal and permanent atrial fibrillation favor the hypothesis that low-grade chronic systemic inflammation is the cause and not a repercussion of these condition [263]. It is obvious that each factor discussed in Section 4.5 may affect systemic inflammation in different yet additive ways, and genetic influence is also possible, since it was discussed that differences in baseline CRP levels have also been attributed to genetic polymorphisms in the promoter of the transcribed CRP gene. Yet, systemic inflammation seems more likely to be significantly affected by internal acidosis, which is extremely common from various aspects. Food-derived acids followed by inhaled toxins which are both so common and proven risk factors for most diseases, are also mainly associated with increased CRP. These diseases are actually the result and not the causal effect, which seems to be the internal lower microenvironmental pH that leads to systemic inflammation and possibly to the bacterial transformation and genesis of more pathogenic and virulent bacterial colonies that cannot be completely fought off by the already inflamed tissues, and due to this, tissue death is probable. Moreover, it is important to highlight, that pneumococci colonies may be increased due to high choline uptake (mainly in such acidic foods, i.e., meat, fish, poultry, dairy, and eggs) since it is their nutritional requirement, and such scenario may finally aid in more frequent transformations; interestingly, CRP has been found to be increased in such nutritional standards that also have a direct negative impact on human health. Streptococci (lactic acid bacteria) prefer to consume easily metabolizable carbohydrates, like glucose, if they are available in the microenvironment so as to produce energy. They may require other nutrients that are also part of the human diet, and they are notable contributors to tooth decay, thus it is obvious that they are parasites in our body, and that there is no real asymptomatic symbiosis, but rather parasitism that can trigger even human death.

Some studies report CRP to be higher in adult elderly patients, but such increase is in reality due to age and not disease. Doubtlessly, age is considered to be the first and foremost comorbidity for all individuals, and also, the more years one lives, the more the possibilities for higher internal acidosis as well as for bacterial colonies in human body to be multiplied. Related hypotheses could explain several CRP variations in diseases; thus, it is not the association between CRP and the disease that really matters, but all these underlying logical associations. Elevated hsCRP is mainly highlighted metabolic issues that can further trigger cardiovascular risks, and one could argue that the gastrointestinal tract, which is basically the way by which the organism communicates with its external environment (along with the respiratory tract), is in charge of the digestion and production of acid metabolites, thereby obviously initiating internal acidosis. It is already known that a high percentage of the immune system is located in the gut, thus there are higher possibilities for such colonies to be transformed. Importantly, it has been demonstrated that the extracellular bacterial lymphatic metastasis of the virulent strains of certain streptococci drives systemic infection, and there have also been illustrated multifarious routes and mechanisms by which an increasing variety of bacteria are acknowledged to transit through the lymphatic system, including those that do not necessarily require internalization by host cells [1224]. Therefore, one can already have organ inflammation and possible subsequent other system inflammation (i.e., nervous system inflammation) due to systemic inflammation, and realize it after being symptomatic for any system (i.e., muscle symptoms and underlying systemic acidosis) by random or due to genetic factors. Other data have revealed that in chronic lymphatic obstruction, even during pathogenic infections, CRP is elevated [1225]. It is already known that body acidosis leads to lymphatic obstruction since this is the actual cause (it is recommended that in case of lymphedema, one needs a healthy and mostly vegan diet). Nevertheless, it should be highlighted that according to the Academy of Nutrition and Dietetics, a food is considered an “acid-ash” food based on the ash derived after the combustion of foods under laboratory conditions [1226]. Acids are produced in cells through metabolism, as they consume and eliminate their nutrition via the blood, and their discharge is waste, which ultimately reaches the lymphatic system. As a result, undeniably, BMI is not applicable, which several studies take into account when assessing CRP in parallel with CVDs or other conditions, because even if obesity were always a prognostic factor for elevated CRP and poor outcomes, some people have normal BMI but consume high percentages of foods that boost acidosis, and as a result, biases arise in these studies (that report no BMI association with the analyzed medical condition). There also exist several studies that associate CRP in cases with more than one disorder. One can obviously speculate that between two population samples, in which one is characterized by less metabolic acidosis and the other seems more likely to show slightly elevated hsCRP, which can possibly be associated with any type of further disorder(s), certain deviations are likely to be due to other factors, as mentioned in Section 4.5 and genetics. In this way, various types of bias arise and lower the quality of the existing studies on CRP and its possible role as a marker in disease, although in reality, it is not a marker but a result of systemic acidosis and following chronic low-to-moderate systemic inflammation.

Indeed, apart from the dietary parameters, almost all factors discussed in Section 4.5 have been linked to human microbiome imbalance, which is actually due to bacterial transformation. Wounds and tissue traumas, surgeries, and interventions such as catheters, antibiotics/hormonal/chemotherapy/other medications, drug abuse, vaccines, exercise, air pollution, sun exposure, temperature and climate change, electromagnetic radiation, pets, sexual preferences, sleep, religion, and generally the overall lifestyle contribute to alterations in microbiota, some of which may be more pathogenic and infect tissues, resulting in cell and further tissue necrosis, and death [1227,1228,1229,1230,1231,1232,1233,1234,1235,1236,1237,1238,1239,1240,1241,1242]. It is obvious that external factors, mostly nutrition and drugs, vaccines, weather and temperature, and radiation are some of the most important factors that can contribute to internal acidosis, bacterial transformations, and further inflammatory cascades. These factors can act like antibiotics toward bacteria and result in bacterial imbalance and the transformation of more resistant colonies that can possibly infect human cells. Such scenario can lead to cell and further tissue apoptosis and necrosis, and analogous to its magnitude, it can ultimately cause an individual’s death. One could further say that acidosis may trigger bacterial mutations in order for such microorganisms to survive in more acidic microenvironments as well.

**Figure 5:**
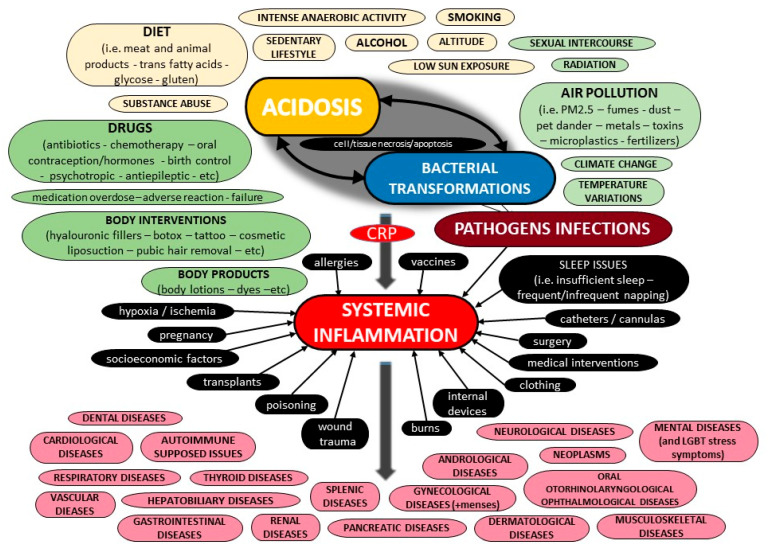
Potential factors contributing to systemic inflammation.

Figure 5 illustrates the previously discussed concept. Acidosis resulting mainly from diet—which can trigger it directly or indirectly—can trigger bacterial transformations, which in turn can boost acidosis mostly via their products and infections (positive feedback loop). Such mechanisms can lead to cell apoptosis/necrosis, further tissue damage, and the formation of various immunocomplexes. The factors in yellow basically affect acidosis, while factors depicted in green have an impact on both acidosis and bacterial transformations. Pathogenic infections can be either originate from bacterial-converted virulent colonies or from other pathogenic infection. Factors in black can also trigger systemic inflammation regardless of pre-existing acidosis and bacterial transformations. Finally, systemic inflammation, characterized by the presence of CRP, leads to further physiological disorders, autoimmune-supposed conditions, and neoplasms.

It has been discussed that CRP production precedes the generation of a specific IgG response by at least one week. Thus, it may play a crucial role in activating the immune response prior to the development of an adaptive immune response, and also, CRP as a pentraxin generally controls inflammation and autoimmunity in many ways [63,80,659]. Yet, microbial infection is a major driving force of change during evolution, and it was proposed that in case CRP is a relevant component of innate immunity, the inducibility or tissue-specificity of its expression may be at least as crucial as chronic circulating levels [269]. Could CRP not really be a part of innate immunity, but rather another a unique entity simply in charge of all further inflammatory cascades [1143]? Could certain bacterial transformations in specific conditions lead to more vulnerable bacteria, as some studies have found that CRP has a protective role? However, CRP production is significantly different in lab mice versus human organisms [660]. If it is the case that CRP has a protective role and aids in ceasing inflammation through stimulating further cascades to tackle its cause, then why are there various drugs designed against this molecule that is considered to be a part of innate immunity? For example, it was found that CRP may predict poor outcomes in cancer cases but it also may have tumoricidal activity, so why are there therapeutic strategies proposed to lower CRP levels [1144]? Could such therapies lead to further bacterial transformations and subsequent diseases in already ill patients? It is evident that data on CRP seem extremely contrasted. Could the various aggregated protein-/immunocomplexes that exist in acidic inflammatory microenvironments be directly attributable to all such controversies possibly through various lab misdiagnoses?

One should bear in mind that since vaccines and immunomodulation is believed to be the best method to tackle with pathogens, autoimmune-supposed conditions, and chronic inflammation, a large percentage of the future population may be considered to have globulinemia, a fact that may yield false results in immunoassays [1042]. In such conditions, acidic inflammatory microenvironments, which may be more prevalent, can trigger the formation of more and various aggregated protein-/immunocomplexes, which then may be followed by the transformation of more resistant and virulent bacteria, thereby contributing to these proteinocomplexes, and also to trigger further pathologic damage. It is speculated that such proteinocomplexes may boost the discovery of more CRP forms, and that it is possible for there to be some altered and degraded CRP peptide products. In such microenvironments, divergent crystal packing forces can affect the stabilization of a particular CRP arrangement, and substantial face-to-face contact between adjacent CRP molecules/fragments can result in misidentified forms of CRP.

It is evident that the sandwich-like serologic methods and particularly their targets need to be evolved soon [1042]. Since HAs, paraproteins and other proteins precipitate under specific assay conditions, whether basic, acidic, or even neutral pH, it would be very difficult to design an ideal assay that prevents all those instances, but most manufacturers have optimized their immunoassays to reduce this problem; however, other issues arise. More studies are needed so as to reveal possible reasons of CRP false test results—mostly based on interferences, cross-reactions, etc. Additionally, since future diagnostic assays are already designed, their limitations should be studied starting from now, so as to allow physicians to be able to identify and avoid them. In addition, studies reporting CRP test results must also report the name of the test kit and the method that was performed to obtain the results in order for those who study them to be able to see possible reasons for misdiagnoses. Also, the overall medical history of a case should be thoroughly reported, as various factors mentioned in previous sections can contribute to elevated CRP test results, apart from the reported medical conditions. Furthermore, the primary structure of human CRP was examined for internal homology and compared to all known proteins whose structures were published before April, 1978, by two computer programs, and distant homologies have been noted regarding the Ca2 domain of IgG and C3a anaphylotoxin [1210]. Since CRP shows homology with both pneumolysin and also with these human proteins, could it simply be the conception of a protein made of both active and inactive aggregated peptides? Could its gene be modified by both human and bacterial fragmental sequences? Undeniably, such studies are required for CRP to be monitored for potential homology with other proteins or other molecules (i.e., drugs, food-derived molecules, etc.) in order for possible false test results to be prevented. Also, potential CRP homology with other human proteins should be studied to prevent potential immunoglobulin cross-reactions and further autoimmunity. Furthermore, since anti-CRP antibodies have been detected, could CRP be considered as a self-antigen, thus leading to a novel autoimmune-like condition? Also, could such antibodies be designed for some non-CRP epitopes but be depicted as binding to CRP just because of possible homology and cross-reactions? One could hypothesize that because other factors (Section 4.5) that can affect CRP levels are increasing nowadays, due to these other factors, some (auto)antigens found in acidic inflamed microenvironments may trigger more cross-reactions between them and such antigens as well. Finally, since the real role of CRP seems to be unknown, more studies are required to reveal the real role of that molecule basically by studying these other factors, which, in reality, lead to its higher levels (or to its existence).

## 7. C-Reactive Protein: A Novel Diagnostic Algorithm Every Physician Should Know

The current literature data regarding the diagnosis of CRP seem diffused and conflicting. Nevertheless, all medical professionals, but particularly front-line physicians in Emergency Departments (EDs), as well as others in medical clinics and senior care facilities, rehabilitation centers and other centers, ought to be able to make a prompt and accurate assessment of a CRP test result. First and foremost, the precise diagnosis of a disease requires medical history, physical examination, and pathognomonics, as well as radiologic and laboratory evidence. Such parameters are needed for the overall assessment of a CRP test result, too. It is evident that the overall diagnosis of a case’s medical condition consists of both macrodiagnosis and microdiagnosis.

Medical history is of vital importance, but in reality, the overall history of a patient’s life is required in order for a physician to draw direct conclusions for the underlying systemic inflammation, as there have been various factors discussed in Section 4.5 that may contribute to this condition. The time of sampling may be considerably earlier or later than the onset of the actual medical condition that a physician is trying to diagnose. For instance, it was discussed that CRP values can fluctuate by the day, that blood samples collected in EDs may be those before elevated concentrations of CRP, and that they have been known to reach their peak between 36–50 h after the onset of infection [1243,1244,1245]. Moreover, a prolonged positivity ought to be further questioned, and also, a CRP test result is not able to reveal whether CRP is disseminated in the human body or is of local production. One could argue that cases with infections from more than one pathogen or those with more than one transformed bacterial colony may trigger false results for the direct pathogenic agent that is responsible for the infection, or in the case where the patient is vulnerable, more than one pathogen may be the cause of the final condition or the disease. Moreover, it was discussed in previous sections that CRP may not predict infections due to less virulent pathogens, and possibly in healthier individuals.

Furthermore, the time of sampling is of vital importance since the individual may not have only eaten or drunk, medications, supplements, and other products before sampling, but they may have also routinely consumed any of the specific products that were discussed in previous sections, which may cause a false test result, even if not consumed on the specific day of sampling (i.e., biotin supplements, certain drugs, etc.). Undeniably, some laboratories instruct their patients to fast only for those tests for which values will be influenced by food intake, including glucose, lipids, and calcium, but in reality, this depends on the assay rather than the target detected. It has been proposed that the use of suitable reference intervals (i.e., age and sex) and contextualization (i.e., therapy, fasting state, posture (i.e., renin)), physical activity (i.e., increased D-dimers, N-terminal pro-brain natriuretic peptide, cTn in elite athletes), circadian cycle, and stress are major factors to be taken into account before suspecting an interference [1156].

It is obvious that physicians must be capable of diagnosing a potential misdiagnosis, and predict a possible CRP false test result since several factors have been discussed in Section 5.2 that may lead to a misdiagnosis. Firstly, frontline professionals should be aware of their laboratory test kits’ limitations so as to be able to identify which case may show a false test result. Potential tricky cases that may show misleading test results should be noted to laboratory personnel so as to manage this particular clinical specimen appropriately. Of course, as previously discussed, cases with various underlying medical conditions or other factors that may result in high serologic load (that affects most current CRP assays) should be thoroughly examined, and such CRP test results should be assessed in parallel with other potential critical biomarkers for the accurate diagnosis of a disease. Further information for factors that may trigger immunoglobulin interferences (i.e., HAs, HAAAs, vaccinations, drugs, etc.) should also be recorded in the history of a patient. The type of sample (i.e., blood, serum, BAL, CSF, etc.) can possibly be alternative, analogous to the difficulties of the initial sample. Generally, retesting, resampling and alternative sampling seem to be some favorable methods for preventing misdiagnoses. Indeed, the value of resampling has been highlighted for CRP in EDs [1246]. Regarding alternative sampling, it was discussed that certain analytes also appear in urine, and because endogenous antibodies are not supposed to be frequently present in urine, a discrepancy between urine and serum concentrations may suggest interferences [1159]. Doubtlessly, CRP values obtained from different test kits, methods, and laboratories, and in different points of time cannot be compared. One should not forget also that a test result is just a random value for a random sample at a random point of time, and as a result, a test cannot tell what is causing inflammation, and also, test results vary from lab to lab. Furthermore, some laboratories may run more than one CRP test kits, so the clinical sample tests for the cases that may lead to some potential ambiguous test results should be performed with the most sensitive assays and not just with the any regular assay. It is evident that clinical doctors and laboratory professionals should cooperate in such ways in order for a case to be precisely diagnosed. Additionally, it is evident that a large percentage of the population is infected but also vaccinated against COVID-19, and even such scenarios could be the etiology of possible misdiagnoses [1,2,3,4,5]. Moreover, a case may present another undiagnosed underlying medical condition, which could be the real cause of an elevated CRP test result apart from the condition that a clinician tries to diagnose (i.e., undiagnosed autoimmune-supposed condition due to no initial relapse or no identification of it). It seems complicated to estimate whether high CRP values reflect acute inflammation or chronic inflammation [1245]. That is, because CRP is affected by several factors, as discussed in Section 4.5, an individual with fewer factors affecting their immune system may show a relatively increased CRP in case of an infection, and inversely, an individual with various factors affecting their immune system may show notably elevated CRP values, but in reality, a small percentage of its increase may be due to a possible infection. In other words, the more pre-existent the other factors (as discussed in Section 4.5), the higher the baseline CRP value for possible acute inflammation in a case, and vice versa. It should be highlighted that medications, diet, or other factors may lead to a lower baseline CRP for certain individuals, therefore an infection may yield lower CRP test results compared to others—despite the predictable trends in CPR increased values and their significance as a biomarker of a specific disease. Importantly, undeniably, age is the first and foremost comorbidity, and should also be evaluated in the accurate diagnosis of CRP. Nowadays, more and more people are extremely vulnerable; systemic inflammation is highly prevalent due to current lifestyles and other factors, and thus, misdiagnoses may be more common than in previous years. Generally, all factors discussed in Section 4.5 may yield different CRP test results, and they should be assessed additively for the final CRP test result. Regardless of such parameters, cases with various underlying issues or a possibility of the Hook effect should be noted to laboratory personnel in order for their samples to be managed appropriately. Previous sections revealed that the more medical conditions or factors presented in a case that trigger systemic inflammation, the more likely it is for the CRP test result to be even more increased due to possible interferences (i.e., HAs, paraproteins, etc.). Even if, typically, conditions like paraproteinemia were to affect ESR, it cannot be excluded that even CRP test results can be affected if its specific diagnostic assay can be affected, too.

Moreover, it is possible for a test to identify various CRP forms and trigger false test results, since there are various altered CRPs, and also, no one can know if a test identifies CRP fragments undergoing degradation, rather than initial active CRP molecules. Also, in case of CRP SNPs and other genetic parameters (i.e., SNPs in the promoter of CRP gene), a test may be negative, but in reality, CRP may exist in notable concentrations. On the contrary, a CRP test result may be positive, but in the case of mutated CRP, it was discussed that mutants may not be able to bind to ligands/receptors, but such a misdiagnosis cannot be identified by a physician. In certain cases, EDs and other facilities should be properly equipped with very sensitive quick diagnostic instruments, allowing front-line physicians to perform a rapid diagnosis of CRP on their own to avoid delays and waiting for laboratory responses for a severe or critical case that needs rapid management and treatment.

**Figure 6:**
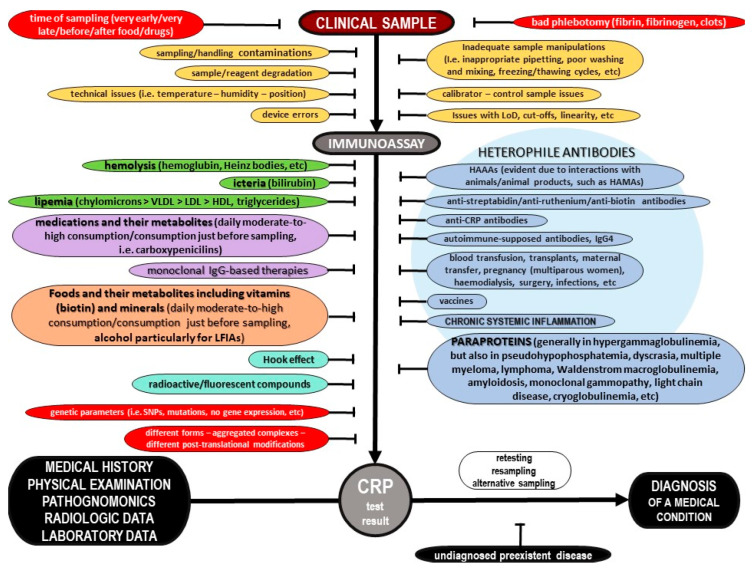
A novel diagnostic algorithm to carefully assess the CRP level of a patient.

Overall, Figure 6 illustrates a proposed novel state-of-the-art algorithm for physicians to make an accurate assessment of a CRP test result and the further accurate diagnosis of a medical condition. Some important factors that may affect the final CRP test result and further diagnosis of a medical condition are presented in red, preanalytical factors are presented in yellow, while factors possibly affecting the immunoassays for CRP and inhibit the final correct CRP test result are seen in other colors. Of course, an undiagnosed medical condition may yield misdiagnoses, whereas in case of the suspicion of a false CRP test result, retesting, resampling, and alternative sampling is recommended.

It seems that any discussion of the diagnostic value of CRP in blood must include the timeframe and disease conditions occurring when samples were collected, and of course, there may be differences in hsCRP and conventional CRP levels. It must be highlighted that such issues can have an impact on more human samples, i.e., bronchoalveolar lavage or lumbar puncture samples.

Finally, a physician must recognize that a (CRP) test result is not a substitute of the overall diagnosis (macrodiagnosis and microdiagnosis), and no diagnosis of a medical condition should be directly based on a sole CRP test result. For instance, a combination of CRP with other biomarkers, such as ESR, could be of more interest in clinical practice. Yet, it must be highlighted that it is obvious that the various CRP assays used in clinical and research labs do not give the same results in various clinical conditions, and all these various CRP testing assays and further results may not correlate with ESR or/and other biomarker in various underlying medical diseases.

## 8. Conclusions

To close the CRP topic, it seems that current literature provides no substantial and accurate evidence toward the real role, functions, nor its possible use as a predictor or a biomarker of disease. Systemic inflammation needs to be better analyzed in parallel with diet, gut microbiota, the immune system, and the lymphatic system, which obviously plays a tricky role in the pathophysiology of most diseases. Molecular diagnostics, which is our most promising tool, should be studied for all potential factors that could have a negative impact on the final test result. The assessment of a molecular diagnostic CRP test result should be based on a clever and ideal algorithm that combines all factors that could contribute to a final misleading test result, thus triggering misdiagnosis, but, on the contrary, even if a real test re-sult can be obtained, the precise role of the hypothesized CRP in parallel of diseases is currently not clear –as proven by current literature. Physicians ought to be capable of identifying the rational cause of increased CRP in a case’s sample. In the near future, scientific communities will provide more data toward all the topics analyzed in this thorough and state-of-the-art critical review article on CRP.
